# Within-species contamination of bacterial whole-genome sequence data has a greater influence on clustering analyses than between-species contamination

**DOI:** 10.1186/s13059-019-1914-x

**Published:** 2019-12-18

**Authors:** Arthur W. Pightling, James B. Pettengill, Yu Wang, Hugh Rand, Errol Strain

**Affiliations:** Center for Food Safety and Applied Nutrition, US Food and Drug Administration, College Park, MD USA

**Keywords:** *Listeria monocytogenes*, *Salmonella enterica*, *Escherichia coli*, Whole-genome sequencing, Contamination, Phylogenetics, Single-nucleotide polymorphism, Multi-locus sequence typing, Comparative genomics, Clustering analyses, SNP, MLST

## Abstract

Although it is assumed that contamination in bacterial whole-genome sequencing causes errors, the influences of contamination on clustering analyses, such as single-nucleotide polymorphism discovery, phylogenetics, and multi-locus sequencing typing, have not been quantified. By developing and analyzing 720 *Listeria monocytogenes*, *Salmonella enterica*, and *Escherichia coli* short-read datasets, we demonstrate that within-species contamination causes errors that confound clustering analyses, while between-species contamination generally does not. Contaminant reads mapping to references or becoming incorporated into chimeric sequences during assembly are the sources of those errors. Contamination sufficient to influence clustering analyses is present in public sequence databases.

## Main text

Whole-genome sequence (WGS) analysis is valuable for studying bacteria in many disciplines, including genetics, evolutionary biology, ecology, clinical microbiology, and microbial forensics [1–5]. Researchers cluster genomes with phylogenetic analyses and by counting nucleotide or allele differences. Contamination of eukaryotic data can cause misleading results [6, 7]. For prokaryotes, it is assumed that contamination causes error [8], and tools are available to detect it [9–13], but evidence supporting this assumption is lacking. To measure the influences of contamination on clustering analyses, we generated 720 sets of simulated *Listeria monocytogenes*, *Salmonella enterica*, and *Escherichia coli* Illumina MiSeq reads. These datasets include from 10 to 50% of within-species (at 0.05, 0.5, and 5% genomic distances) and between-species contamination. We also identified 24 sets of closely related bacteria (clusters) within which the contamination datasets can be analyzed. With these tools, we found that within-species contamination caused substantial errors in single-nucleotide polymorphism (SNP) and multi-locus sequence typing (MLST) pipelines, while between-species contamination resulted in fewer errors. Read mapping and assembly behavior explains this observation—reads from the same species are mapped to references or incorporated into the same contiguous sequences (contigs) as subject reads, while reads from different species usually are not.

We measured SNP and allele distances between subjects and closely related isolates (“nearest neighbors”) with the CFSAN SNP Pipeline and core-genome MLST (cgMLST) workflows [14–16] (Additional file 1: Table S1). We also performed phylogenetic analyses to provide bootstrap supports for the monophyly of subjects and their nearest neighbors. Importantly, only the subject data are simulated; all other data are real (Additional file 1: Figure S1). This approach provides as realistic a dataset as possible that produces results that apply to real-world situations.

**Fig. 1:**
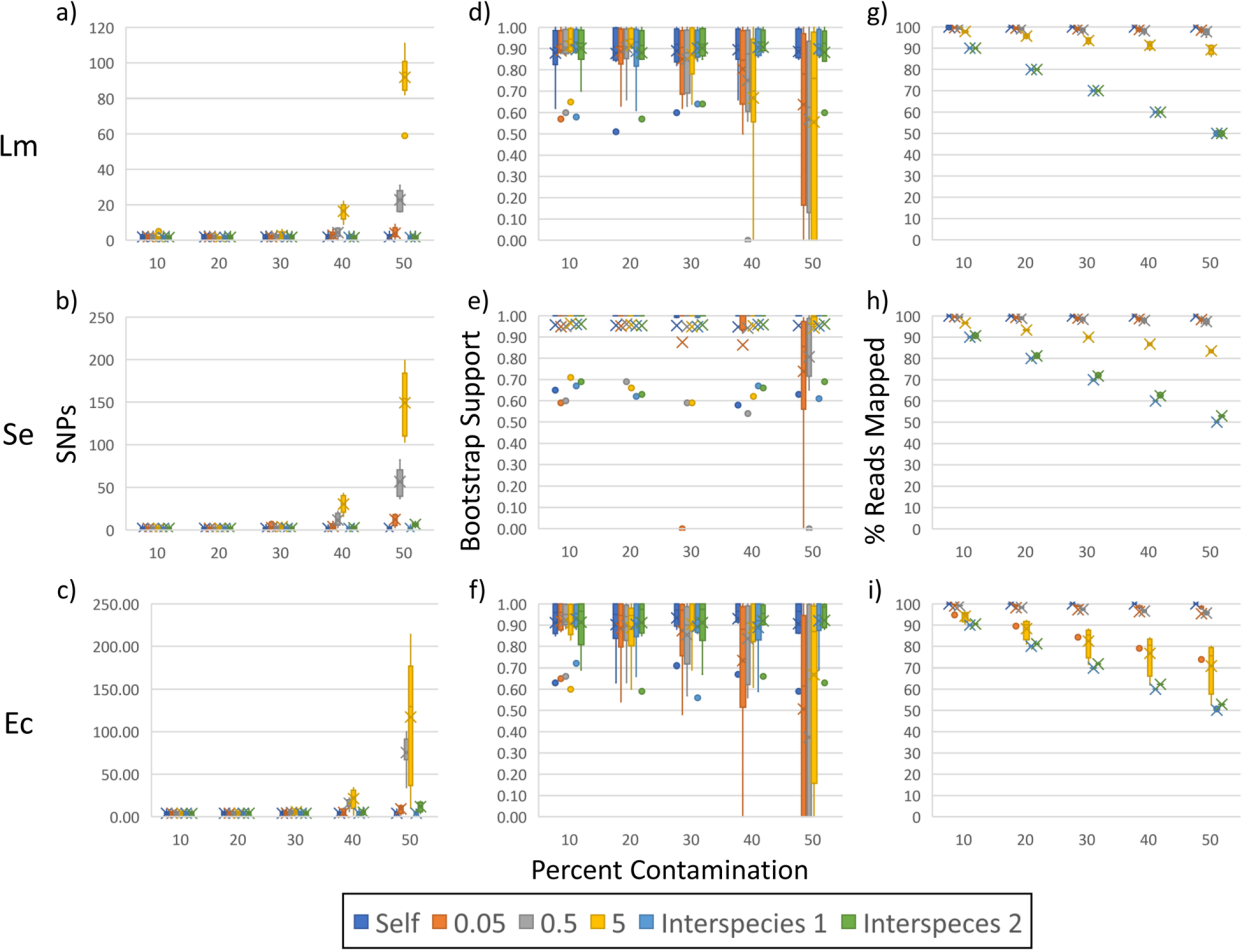
Results of SNP and phylogenetic analyses for contaminated datasets. We contaminated simulated *Listeria monocytogenes* (Lm), *Salmonella enterica* (Se), and *Escherichia coli* (Ec) MiSeq data with reads from themselves as controls (Self); genomes from the same species at 0.05, 0.5, and 5% genetic distances; and genomes from different species (e.g., we contaminated Lm with Se and Ec, and we contaminated Se with Lm and Ec) at 10–50% levels. For each contamination type at each level, results for 8 datasets are shown. Panels **a**-**c** show SNP distances, **d**-**f** bootstrap supports, and **g**-**i** percent reads mapped

**Fig. 2:**
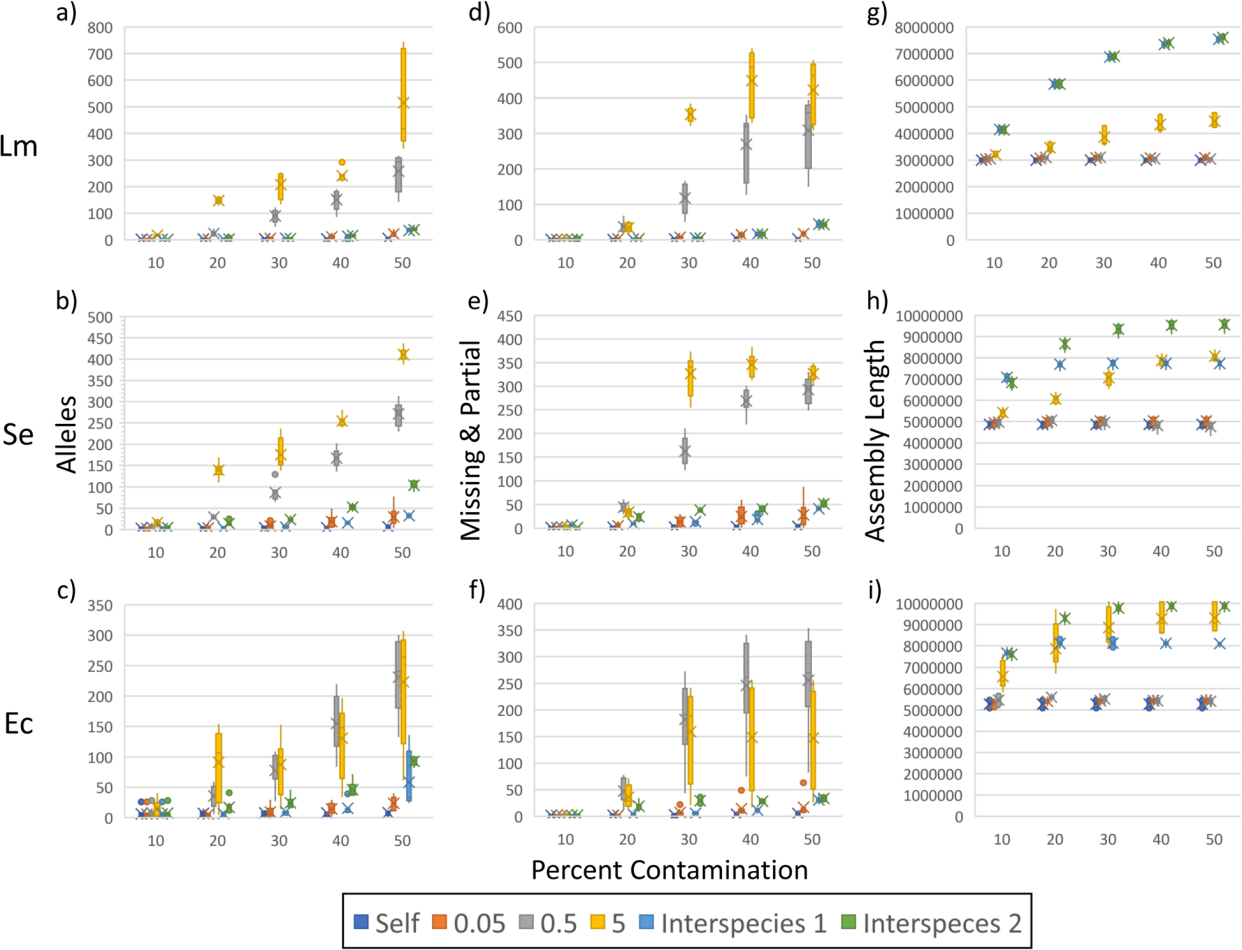
Results of MLST analyses and assembly lengths for contaminated datasets. We contaminated simulated *Listeria monocytogenes* (Lm), *Salmonella enterica* (Se), and *Escherichia coli* (Ec) MiSeq data with reads from themselves as controls (Self); genomes from the same species at 0.05, 0.5, and 5% genetic distances; and genomes from different species (e.g., we contaminated Lm with Se and Ec, and we contaminated Se with Lm and Ec) at 10–50% levels. For each contamination type at each level, results for 8 datasets are shown. Panels **a**-**c** show allele counts, **d**-**f** numbers of missing and partial alleles, and **g**-**i** assembly lengths

We observed increased SNP counts for all three species at 40 and 50% levels of contamination with 0.5 and 5% distant genomes (median 5–154) relative to controls (median 1–3; Fig. 1a–c, Additional file 1: Tables S2 and S3). For *S. enterica* and *E. coli*, there were smaller but significant increases at 50% contamination with 0.05% distant genomes (median 12–14) and for one of the two between-species contaminants (median 7–13). Bootstrap support at 40 and 50% levels of within-species contamination decreased for *L. monocytogenes* and *E. coli* (median 0.63–0.88 and 0.00–0.92, respectively) compared to controls (median 0.91–0.92 and 0.97), although not all decreases were significant (Fig. 1d–f). For *S. enterica*, we saw small decreases with 50% contamination by 0.05 (median 0.86) and 0.5% (median 0.96) distant genomes relative to controls (median 1.00 for each). For *L. monocytogenes* and *S. enterica*, between-species contamination caused no decreases in bootstrap support (median 0.92–0.93 and 1.00, respectively), and support only slightly decreased for *E. coli* (median 0.92–0.99). With the MLST workflows, each type of contamination influenced allele counts. Still, the 0.5 and 5% distant genomes had the greatest influence (median 3–294 and 14–418) when compared to controls (median 2–5; Fig. 2a–c, Additional file 1: Tables S2 and S3). The numbers of missing and partial alleles were also greatest for the 0.5 and 5% contaminants (median 1–463) relative to controls (median 0–6; Fig. 2d–f). Errors at lower levels for the MLST are likely due to the absence of filtering steps commonly found in SNP pipelines.

To gain insight into these results, we examined the percent of reads mapped to references. Median values were highest for 0.05 and 0.5% within-species contamination (median 96–100%) and lowest for between-species (median 50–91%), while 5% within-species contamination yielded intermediate results (median 76–98%; Fig. 1g–i, Additional file 1: Tables S2 and S3). For between-species contamination, there is an inverse relationship between contamination levels and the percent of reads mapped to references. For example, at 10% contamination, approximately 90% of reads mapped. It appears that the more distant mapped contaminant reads are, the higher the SNP counts. Contaminant reads that are similar enough to the reference to be mapped but distant enough from the subject to introduce variation will generate errors. In turn, these errors may reduce bootstrap support. A similar relationship exists between allele distances and assembly lengths. Median assembly lengths for 0.05 and 0.5% within-species data are similar to controls (median 3.0–5.6 and 3.0–5.3 megabases [Mb], respectively), while between-species contaminants yielded larger assemblies (median 4.1–9.9 Mb) and the 5% within-species contamination dataset yielded intermediate assemblies (median 3.1–9.1 Mb; Fig. 2g–i).

To measure contamination in public sequence databases, we used ConFindr [13] to analyze 10,000 randomly selected fastq datasets for each of *L. monocytogenes*, *S. enterica*, and *E. coli* (Additional file 2: Table S4). We detected contamination in 8.92, 6.38, and 5.47% of the data, respectively (Additional file 1: Table S5). We detected between-species contamination (1.23, 0.29, and 0.15%) less often than within-species contamination (7.69, 6.09, and 5.33%), consistent with Low et al. [13]*.* We also analyzed the simulated data with ConFindr and used that information to estimate levels of contamination in the databases that may confound SNP and MLST workflows (Additional file 1: Figure S2 and Table S5). Approximately 1.48 (*L. monocytogenes*), 2.22 (*S. enterica*), and 0.87% (*E. coli*) of the data are contaminated at levels that are likely to influence SNP analyses. Roughly 2.26 (*L. monocytogenes*), 5.06 (*S. enterica*), and 1.26% (*E. coli*) of the data are contaminated at levels that may influence MLST analyses.

In summary, we show that within-species contamination (especially by 0.5 and 5% distant genomes) causes more errors in SNP counts, allele counts, and phylogenetic analyses of bacterial genomes [17] than between-species contamination. While other workflows may not yield the exact numbers measured here, the observation that contaminant reads are mapped to references and included in contigs of the same species, resulting in errors, is likely to hold. This study also shows that contamination that may cause errors in clustering analyses is present in public sequence databases. Therefore, it is important that studies include steps to detect within-species contamination.

## Methods

We searched the National Center for Biotechnology Information’s (NCBI’s) database for closed *Listeria monocytogenes*, *Salmonella enterica*, and *Escherichia coli* genomes (e.g., “*Listeria monocytogenes*”[Organism] AND (“complete genome”[filter] AND all[filter] NOT anomalous[filter])) and downloaded all assemblies. We identified those that are 0–9 SNPs distant to other genomes (“nearest neighbors”) using the “min_dist_same” and “min_dist_opp” measurements in the NCBI metadata files [18–20]. We used the NCBI’s Isolates Browser [21] to identify closed genomes with closely related isolates that are part of NCBI SNP trees with at least 5 taxa [22]. We assembled 16,839 *L. monocytogenes*, 127,357 *S. enterica*, and 33,821 *Escherichia coli* Illumina datasets with SPAdes v3.12.0 (spades.py --careful -1 forward.fastq -2 reverse.fastq) [23]. We removed contigs that were less than 500 nucleotides. We aligned closed and draft assemblies with NUCmer v3.1 (nucmer --prefix=ref_qry closed.fna draft.fna) and estimated SNP distances with show-snps (show-snps -Clr ref_qry.delta > ref_qry.snps) [24]. We selected closed genomes for further analyses that are approximately 0.05, 0.5, and 5% from draft genomes of the same species (based upon closed assembly length estimates calculated with QUASTv4.5 [25]). For most subjects, within-species contamination represents (i) closely related genomes of the same serotype and clonal complex, with 0–2 locus differences (average 0.22; as measured with the program mlst; 0.05%) [26–28]; (ii) distantly related genomes of the same serotype but different clonal complex and 2–6 locus variants (average 4.1; 0.5%); and (iii) genomes of a different serotype and clonal complex with 7 locus variants (average 7; 5%; Additional file 1: Table S1). When unavailable, we predicted serotypes for *S. enterica* with SeqSero [29] and *E. coli* with SerotypeFinder [30]. We generated simulated reads using closed subject assemblies, within-species draft contaminant assemblies, and between-species draft contaminant assemblies, with ART_Illumina v2.5.8 (art_illumina -ss MSv1 -i assembly.fasta -p -l 230 -f 20 -m 295 -s 10 -o paired_data) [31]—all assemblies were generated from real sequencing data. Contamination fastq files were made by randomly selecting subject and contaminant reads at indicated levels (in this case 10–50% contamination) and combining them into paired read files with 20-fold depth of coverage (github.com/apightling/contamination; e.g., select_reads.pl subject_1.fq subject_2.fq 10 contaminant_1.fq contaminant_2.fq output_prefix).

We identified SNP clusters that contain subject genome sequences with the NCBI’s Isolates Browser. If SNP clusters had more than 20 taxa, counting the subjects and their nearest neighbors, we randomly selected subsets for further analyses. We also ensured that the subjects and nearest neighbors formed monophyletic groups in phylogenetic trees. We generated SNP matrices with the CFSAN SNP Pipeline v1.0, using the subject assembly as a reference to minimize errors [32]. Alignments of SNPs that were detected by mapping reads to the reference were phylogenetically analyzed with GARLI v2.01.1067 [33] (100 replicates, K80 and HKY). We reported supports for monophyly of subjects and nearest neighbors; if the they were no longer monophyletic, we recorded a support of 0.

We assembled simulated data with SPAdes v3.12.0 and measured assembly statistics with QUAST v4.5. We analyzed *Listeria monocytogenes* assemblies with the LmCGST core-genome multi-locus sequence typing (cgMLST) tool and *Salmonella enterica* assemblies with an *S. enterica* cgMLST tool described in Pettengill et al. [15]. We analyzed *E. coli* assemblies with a cgMLST developed using the same approach. Partial alleles are those loci whose lengths are less than 60% of the predicted lengths, and missing alleles are those loci that are less than 60% of predicted lengths and less than 80% identical to the reference.

## Supplementary information

**Additional file 1: Figure S1.** Phylogenetic tree of 9 *Listeria monocytogenes* genomes with study subject and nearest neighbor labeled. **Figure S2.** Results of ConFindr analysis of contamination datasets generated for this study. **Table S1.** Contextual information for genome sequences used for this study. **Table S2.** Results of SNP pipeline and core-genome multi locus sequence typing analyses. **Table S3.** *P*-values for results of clustering analyses. **Table S5.** Percent of contamination detected in data from NCBI. **Table S6.** NCBI accession numbers for data generated during this study.

**Additional file 2: Table S4.** ConFindr results from analysis of 10,000 *Listeria monocytogenes, Salmonella enterica,* and *Escherichia coli* fastq datasets. (XLS 7913 kb)

**Additional file 3.** Review history.

## Data Availability

The datasets generated and analyzed during the current study are available in the NCBI repository with BioProject number PRJNA561589 [34]. Accession numbers are listed in Additional file 1: Table S6. The datasets are also available at figshare with DOI 10.6084/m9.figshare.c.4282706.v1 [35]. The scripts made for this study are available in GitHub (https://github.com/apightling/contamination) [36]. An archival version is available at Zenodo with DOI 10.5281/zenodo.3552954 [37].
