# Supplementary material for: Within-species contamination of bacterial whole-genome sequence data has a greater influence on clustering analyses than between-species contamination
Source: Genome Biol. 2019 Dec 18;20:286. doi: 10.1186/s13059-019-1914-x (PMC6918607; doi:10.1186/s13059-019-1914-x)
Supplement: Supplementary file 1 — Additional file 1: Figure S1. Phylogenetic tree of 9 Listeria monocytogenes genomes with study subject and nearest neighbor labeled. Figure S2. Results of ConFindr analysis of contamination datasets generated for this study. Table S1. Contextual information for genome sequences used for this study. Table S2. Results of SNP pipeline and core-genome multi locus sequence typing analyses. Table S3. P-values for results of clustering analyses. Table S5. Percent of contamination detected in data from NCBI. Table S6. NCBI accession numbers for data generated during this study. [file 13059_2019_1914_MOESM1_ESM.pdf]

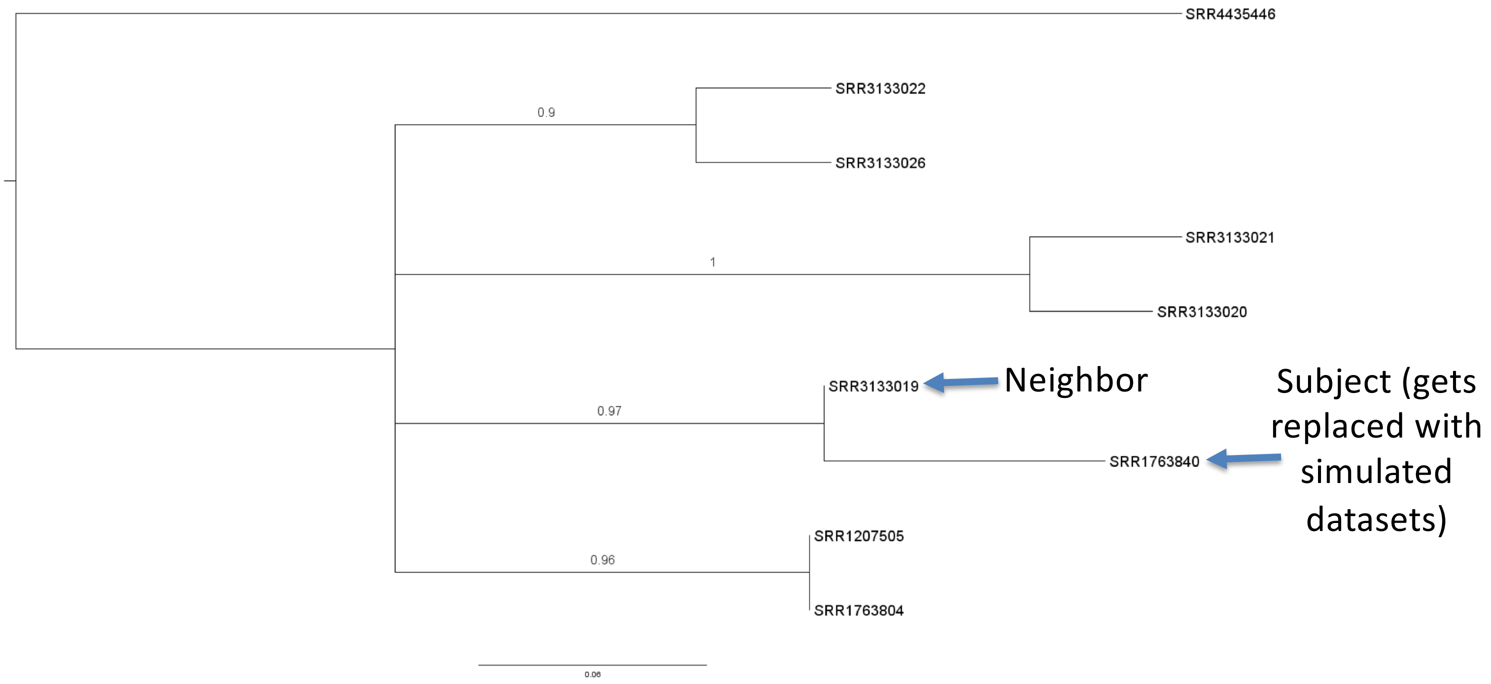

Fig. S1: Phylogenetic analysis of 9 Listeria monocytogenes genomes with study subject and nearest neighbor labeled.
SNP matrices were generated with the CFSAN SNP pipeline and phylogenetically analyzed with GARLI. Bootstrap supports were calculated from 100 replicates. Labels indicate a subject and it's nearest neighbor. All analyses utilize real Illumina sequence data except for the subject, which gets replaced with simulated data.

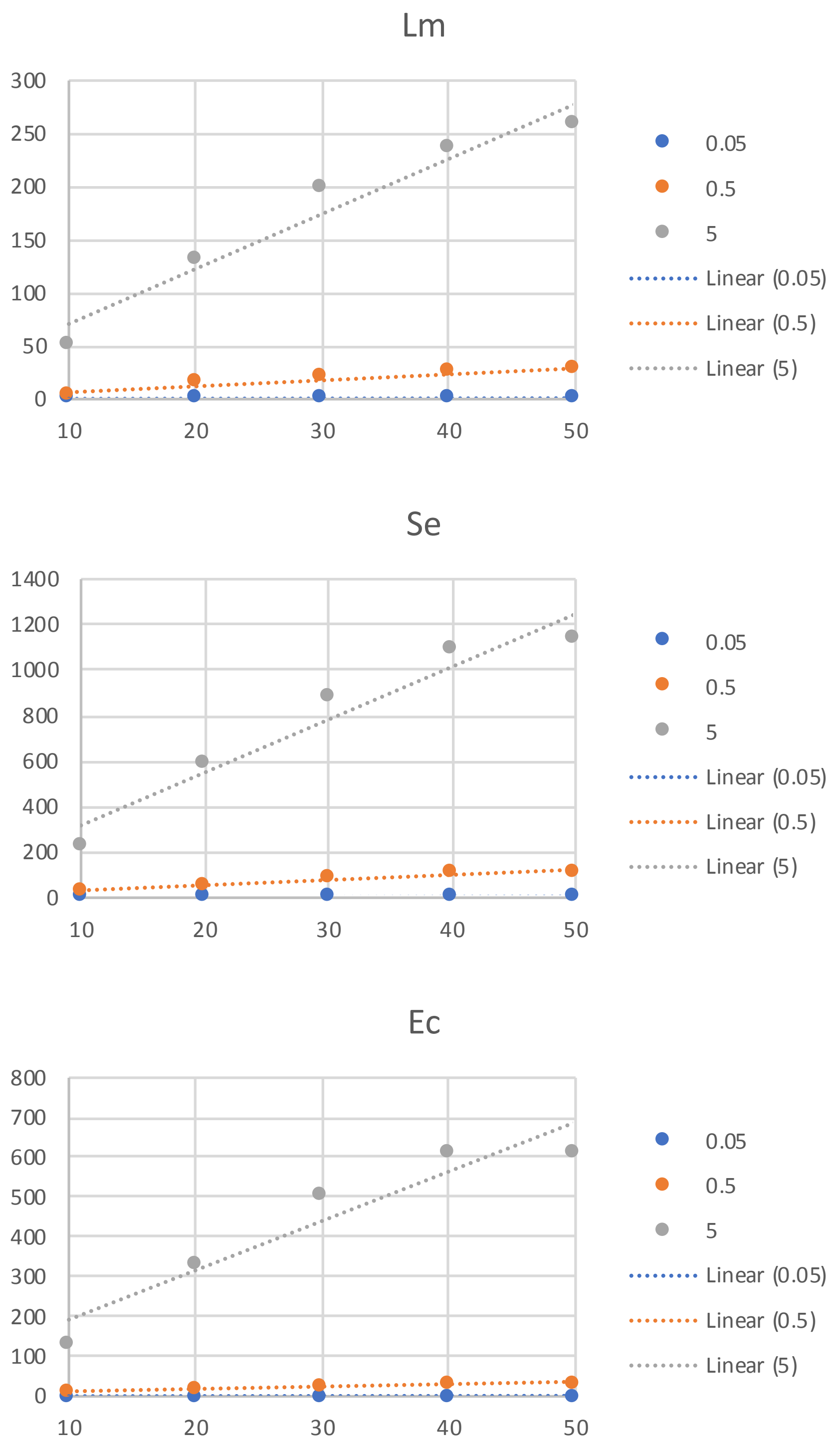

Fig. S2: Results of ConFindr analysis of contamination datasets generated for this study.

Table S1: Contextual information for genome sequences used for this study.

Dataset	Strain	Role	Taxonomy	Serotype	Number Assembly Contigs	Assembly Length (bp)	NCBI Accession (Assembly)	NCBI Accession (SRA)	SNP Count (nucmer)	Distance from Subject (%)	Nearest Neighbor	ST	CC	Lineage	Locus Variants	num SLV	num DLV	Additional Taxa Included in Phylogenetic Analyses
Lm1	HPB5622	subject/contaminant	Lm	1/2a	2	3115752	GCF_001952775.1	SRR1373527	-	-	SRR2000564	292	8	II	-	0	2	ERR1102168 ERR1102169 ERR1102530 ERR1816999 ERR1817015 ERR1817093 ERR1817093 ERR1817095 ERR555038 ERR555039 SRR1745483 SRR1816394 SRR2000564 SRR3108914 SRR3109041 SRR3112458 SRR3114199 SRR3114225 SRR3995871 SRR4048732
	FDA00006686	contaminant	Lm		19	2927271	-	SRR5817942	1529	0.05	-	551	8	II	2			
	CLIP 2015/00134	contaminant	Lm		26	3007123	-	ERR1276239	10488	0.3	-	ND	-	-	2			
	PNUSAL002887	contaminant	Lm		14	2848884	-	SRR5931817	163696	5	-	ND	-	-	7			
	2015-AM-1765	contaminant	Se	Heidelberg	11	4724324	-	SRR5944128	-	-	-	-	-	-	-	-	-	
	74-2388	contaminant	Ec	O79:H2	21	4566697	-	SRR6186591	-	-	-	-	-	-	-	-	-	
Lm2	CFSAN023459	subject/contaminant	Lm	1/2b	3	3105523	GCF_001548585.1	SRR1556286	-	-	SRR1574296	5	5	I	-	1	0	SRR1553784 SRR1553871 SRR1571522 SRR1571523 SRR1571525 SRR1571538 SRR1571540 SRR1571542 SRR1571543 SRR1571543 SRR1571544 SRR1571546 SRR1574290 SRR1574290 SRR1574296 SRR2047250 SRR2047250 SRR5318394 SRR5341774
	FDA00011699	contaminant	Lm		17	3029139	-	SRR5526104	1552	0.05	-	5	5	I	0			
	PNUSAL000316	contaminant	Lm		14	2909915	-	SRR1016606	15493	0.5	-	288	288	I	4			
	CFSAN072501	contaminant	Lm		18	2741194	-	SRR6957404	160518	5	-	565	565	IV	7			
	2015AM-2226	contaminant	Se	Javiana	13	4558278	-	SRR6480957	-	-	-	-	-	-	-	-	-	
	PNUSAE001219	contaminant	Ec	O83:H1	22	4733307	-	SRR2177969	-	-	-	-	-	-	-	-	-	
Lm3	CFSAN012299	subject/contaminant	Lm	1/2a	5	2960190	GCF_001594305.1	SRR1763840	-	-	SRR3133019	7	7	II	-	1	1	SRR1207505 SRR1763804 SRR3133019 SRR3133020 SRR3133021 SRR3133022 SRR3133026 SRR4435446
	CFSAN030330	contaminant	Lm		14	3027066	-	SRR1998940	1460	0.05	-	7	7	II	0			
	PNUSAL001162	contaminant	Lm	1/2a	19	2839821	-	SRR1695819	10476	0.4	-	ND	-	-	2			
	PNUSAL002139	contaminant	Lm		11	2939964	-	SRR3405537	142395	5	-	217	217	I	7			
	PNUSAS041307	contaminant	Se	Oranienburg	12	4597878	-	SRR5947454	-	-	-	-	-	-	-	-	-	
	PNUSAE006873	contaminant	Ec	O167:H26	27	5032835	-	SRR5515964	-	-	-	-	-	-	-	-	-	
Lm4	J1816	subject/contaminant	Lm	4b	1	2947460	GCF_000195435.3	SRR2544677	-	-	SRR1814345	6	6	I	-	1	0	SRR1814345 SRR2915358 SRR6957937 SRR6957938
	N13-1184	contaminant	Lm	4b	17	2923347	-	SRR7442117	1472	0.05	-	6	6	I	0			
	FDA00008897	contaminant	Lm		13	3067637	-	SRR1998941	14728	0.5	-	5	5	I	4			
	FDA00005165	contaminant	Lm		13	3066505	-	SRR3747686	142163	5	-	199	199	II	7			
	PNUSAS018112	contaminant	Se	Saintpaul	13	4762922	-	SRR5947763	-	-	-	-	-	-	-	-	-	
	PNUSAE003319	contaminant	Ec	O103	28	4798508	-	SRR3715600	-	-	-	-	-	-	-	-	-	
Lm5	R2-502	subject/contaminant	Lm	1/2b	2	3091600	GCF_000438585.1	SRR932067	-	-	SRR1623023	3	3	I	-	1	0	SRR1623023 SRR1640129 SRR1686374 SRR2046988
	FSIS11810476	contaminant	Lm		24	3013761	-	SRR7292620	1430	0.05	-	3	3	I	0			
	PNUSAL001248	contaminant	Lm	1/2b	12	2967425	-	SRR1745479	15604	0.5	-	330	288	I	6			
	CFSAN072501	contaminant	Lm		18	2741194	-	SRR6957404	159717	5	-	565	565	IV	7			
	2015AM-2223	contaminant	Se	Widemarsh	13	4759686	-	SRR6480961	-	-	-	-	-	-	-	-	-	
	98-3011	contaminant	Ec	O63:H6	28	4628718	-	SRR5617247	-	-	-	-	-	-	-	-	-	
Lm6	J1776	subject/contaminant	Lm	4b	2	3009523	GCF_000438705.2	SRR932072	-	-	SRR1814341	6	6	I	-	1	0	SRR1055864 SRR1283111 SRR1814341 SRR2047243 SRR2102921 SRR2422614 SRR2422721 SRR2485322 SRR2533825 SRR2533826 SRR2533828 SRR2533829 SRR2540918 SRR2585476 SRR2671210 SRR2671489 SRR3173371 SRR3339951 SRR5448081
	CFSAN035176	contaminant	Lm		15	2922073	-	SRR3394973	1533	0.05	-	ND	-	-	1			
	FDA00011702	contaminant	Lm		16	3029986	-	SRR5571267	15024	0.5	-	5	5	I	4			
	PNUSAL003846	contaminant	Lm		17	3023520	-	SRR7073271	142469	5	-	199	199	II	7			
	CFSAN003970	contaminant	Se	Anatum	13	4708308	-	SRR975397	-	-	-	-	-	-	-	-	-	
	CFSAN080285	contaminant	Ec	O19:H33	28	4593555	-	SRR7085315	-	-	-	-	-	-	-	-	-	
Lm7	C1-387	subject/contaminant	Lm	1/2a	1	2988947	GCF_000438605.1	SRR932055	-	-	SRR1814385	155	155	II	-	1	0	SRR1619234 SRR1619234 SRR1814385 SRR1815992 SRR2650308 SRR2658494 SRR2658495 SRR2658495 SRR3037950 SRR3037953 SRR3101610 SRR3175182 SRR3309230 SRR3309230 SRR5341892 SRR6028670 SRR988742 SRR988742
	CFSAN049301	contaminant	Lm		11	2922182	-	SRR5087886	1441	0.05	-	155	155	II	0			
	PNUSAL001809	contaminant	Lm	1/2a	14	2892543	-	SRR2673275	9232	0.3	-	ND	-	-	3			
	FDA00008468	contaminant	Lm		18	3013519	-	SRR5205362	144358	5	-	288	288	I	7			
	PNUSAS021023	contaminant	Se	Newport	14	4662142	-	SRR5955289	-	-	-	-	-	-	-	-	-	
	PNUSAE001706	contaminant	Ec	O13:H4	29	4868359	-	SRR3040406	-	-	-	-	-	-	-	-	-	
Lm8	CFSAN010068	subject/contaminant	Lm	1/2b	2	3043084	GCF_001548635.1	SRR1191025	-	-	SRR1181538	5	5	I	-	1	0	SRR1181538 SRR1181541 SRR1182223 SRR1182225 SRR1186334 SRR1186341 SRR1187427 SRR1187584 SRR1195657 SRR1195670
	PNUSAL003502	contaminant	Lm		19	3035347	-	SRR6238397	1523	0.05	-	5	5	I	0			
	FDA00007213	contaminant	Lm		12	2998635	-	SRR3930187	15216	0.5	-	3	3	I	5			
	CFSAN003809	contaminant	Lm		16	3050572	-	SRR975368	144141	5	-	321	321	II	7			
	2015AM-2259	contaminant	Se	Paratyphi B	14	4655512	-	SRR6480942	-	-	-	-	-	-	-	-	-	
	HICF208	contaminant	Ec	O156:H14	30	4932761	-	SRR3050978	-	-	-	-	-	-	-	-	-	
Se1	EC20121178	subject/contaminant	Se	Enteritidis	1	4685848	GCF_000623095.1	SRR1183892	-	-	SRR1183770	11	4	-	-	1	0	SRR1183770 SRR1574287 SRR1965538 SRR1969990 SRR3394947 SRR5193845 SRR5194002 SRR5216561 SRR5220932 SRR5676350 SRR5742502 SRR5864546 SRR6897795 SRR6919760 SRR6920120 SRR6922081 SRR6922088 SRR6927006 SRR7359062
	2016K-0430	contaminant	Se	Enteritidis	39	4707318	-	SRR3667831	2348	0.05	-	11	4	-	0			
	AUSMDU00009269	contaminant	Se	Enteriditis	31	4494063	-	SRR6132093	23947	0.5	-	3304	93	-	4			
	PNUSAS039047	contaminant	Se		57	4662598	-	SRR7125364	233710	5	-	4736	-	-	7			
	PNUSAL000834	contaminant	Lm		7	2986303	-	SRR1520085	-	-	-	-	-	-	-	-	-	
	AZ-TG98635	contaminant	Ec	O150:H8	31	4631413	-	SRR6244083	-	-	-	-	-	-	-	-	-	
Se2	CVM 21550	subject/contaminant	Se	Newport	2	4978519	GCF_000272715.4	SRR610464	-	-	SRR1840711	45	3	-	-	2	0	SRR1238765 SRR1384790 SRR1515016 SRR1805560 SRR1840711 SRR2082860 SRR2131025 SRR2566770 SRR3095266 SRR3115334 SRR3294560 SRR3490032 SRR5683895 SRR5890501 SRR6107943 SRR6234213 SRR6255025 SRR6424927 SRR980354
	PNUSAS015354	contaminant	Se	Newport	22	4959570	-	SRR5714198	2460	0.05	-	45	3	-	0			
	FDA00002707	contaminant	Se	Newport	26	4892515	-	SRR1952754	24780	0.5	-	46	3	-	1			
	FDA00010616	contaminant	Se		76	4530623	-	SRR4202526	236095	5	-	3556	-	-	7			
	DK_036	contaminant	Lm		8	2832140	-	ERR1599704	-	-	-	-	-	-	-	-	-	
	FSIS1605645	contaminant	Ec	O181:H14	30	4747279	-	SRR3144198	-	-	-	-	-	-	-	-	-	
Se3	CFSAN001992	subject/contaminant	Se	Javiana	3	4675267	GCF_000341425.1	SRR1029665	-	-	SRR6440593	24	17	-	-	1	1	SRR1292311 SRR1769384 SRR1784319 SRR1812337 SRR1812344 SRR1824462 SRR2915899 SRR2925146 SRR4044583 SRR6327658 SRR6327658 SRR6327668 SRR6327668 SRR6327671 SRR6440593 SRR6475538 SRR7105176 SRR7235871
	PNUSAS008704	contaminant	Se	Javiana	30	4530824	-	SRR5291615	2338	0.05	-	24	17	-	0			
	PNUSAS031785	contaminant	Se	Miami	31	4759393	-	SRR6514374	24373	0.5	-	140	158	-	2			
	PNUSAS024005	contaminant	Se		40	4712933	-	SRR6129500	233655	5	-	4308	-	-	7			
	CFSAN006457	contaminant	Lm		8	2824567	-	SRR2585434	-	-	-	-	-	-	-	-	-	
	PNUSAE005106	contaminant	Ec	O17/O44:H41	30	4871232	-	SRR5438375	-	-	-	-	-	-	-	-	-	
Se4	12-4374	subject/contaminant	Se	Heidelberg	6	4992458	GCF_001441225.1	SRR2896022	-	-	SRR5239232	15	26	-	-	1	0	SRR1363326 SRR1573176 SRR1609687 SRR1686573 SRR1861853 SRR3136893 SRR3289784 SRR3662619 SRR5055297 SRR5236384 SRR5239232 SRR5632690 SRR5714977 SRR5725173 SRR6197784 SRR6297853 SRR6386663 SRR6880697 SRR6881187
	129484	contaminant	Se	Heidelberg	48	4738906	-	SRR7187960	2544	0.05	-	15	26	-	0			
	FDA00003428	contaminant	Se	Saintpaul	28	4747326	-	SRR7284342	24700	0.5	-	2562	361	-	4			
	PNUSAS039007	contaminant	Se	Waycross	52	4815641	-	SRR4202526	234752	5	-	3556	-	-	7			
	DMG1500324	contaminant	Lm		7	2870670	-	ERR1102444	-	-	-	-	-	-	-	-	-	
	ECOL-17-VL-OH-WA-0012	contaminant	Ec	O128:H34	32	4799929	-	SRR5928789	-	-	-	-	-	-	-	-	-	
Se5	CFSAN001387	subject/contaminant	Se	Tennessee	2	4943967	GCF_001535315.2	SRR965704	-	-	SRR1574280	319	238	-	-	1	0	SRR1048252 SRR1048290 SRR1048290 SRR1048298 SRR1048300 SRR1048312 SRR1048330 SRR1233921 SRR1574257 SRR1574257 SRR1574262 SRR1574280 SRR1574283 SRR1574284 SRR1574305
	FDA00001071	contaminant	Se	Tennessee	42	4807040	-	SRR3229336	2240	0.05	-	319	238	-	0			
	CFSAN023681	contaminant	Se	Tennessee	42	5074510	-	SRR1576008	23979	0.5	-	1565	294	-	3			
	08-0004	contaminant	Se		58	4724307	-	SRR5581942	238892	5	-	3925	-	-	7			
	PNUSAL000216	contaminant	Lm		7	2877610	-	SRR1166853	-	-	-	-	-	-	-	-	-	
	CFSAN068008	contaminant	Ec	O150:H8	32	4629646	-	SRR6080586	-	-	-	-	-	-	-	-	-	
Se6	CFSAN001921	subject/contaminant	Se	Typhimurium	4	5089224	GCF_000430145.2	SRR926372	-	-	SRR3665097	19	1	-	-	1	0	SRR1823709 SRR1840615 SRR2989194 SRR3021491 SRR3045981 SRR3240239 SRR3490009 SRR3664769 SRR3664976 SRR3665097 SRR3932881 SRR4280563 SRR5017511 SRR5526144 SRR6350879 SRR6843232 SRR6940358 SRR7071541
	PNUSAS026438	contaminant	Se	Typhimurium	33	4863637	-	SRR6243450	2544	0.05	-	19	1	-	0			
	PNUSAS019972	contaminant	Se	Saintpaul	16	4760683	-	SRR5945527	25491	0.5	-	27	14	-	6			
	PNUSAS031290	contaminant	Se	Waycross	45	4597097	-	SRR6452759	233904	5	-	2509	490	-	7			
	CFSAN006454	contaminant	Lm		8	2825022	-	SRR2650300	-	-	-	-	-	-	-	-	-	
	CFSAN076439	contaminant	Ec	O159:H4	30	4971611	-	SRR6978933	-	-	-	-	-	-	-	-	-	
Se7	CFSAN002069	subject/contaminant	Se	Heidelberg	3	4931985	GCF_000430085.2	SRR1029666	-	-	SRR1616740	15	26	-	-	1	0	SRR1030526 SRR1616740 SRR1616754 SRR1616760 SRR1616765 SRR1805651 SRR1974002 SRR2074983 SRR2156577 SRR2163262 SRR2542452 SRR3490010 SRR3682071 SRR6155476 SRR6726330 SRR7084518 SRR7172543 SRR7184943
	CFSAN032314	contaminant	Se	Heidelberg	45	5011043	-	SRR2039126	2466	0.05	-	15	26	-	0			
	H120280044	contaminant	Se	Kisangani	75	4804443	-	SRR1645762	24695	0.5	-	254	243.2	-	4			
	FDA00010616	contaminant	Se		76	4530623	-	SRR4202526	234619	5	-	3556	-	-	7			
	CFSAN007980	contaminant	Lm		8	3007609	-	SRR1207546	-	-	-	-	-	-	-	-	-	
	PNUSAE003758	contaminant	Ec	O1:H7	33	5050003	-	SRR4054802	-	-	-	-	-	-	-	-	-	
Se8	CFSAN000624	subject/contaminant	Se	Saintpaul	4	5102976	GCF_000487935.2	SRR955194	-	-	SRR1122728	27	14	-	-	1	0	ERR2202570 SRR1122728 SRR1257279 SRR1300656 SRR1300656 SRR1300687 SRR1514699 SRR1514699 SRR1544182 SRR1544246 SRR1544246 SRR1598953 SRR1631192 SRR1698188 SRR1777673 SRR1951876 SRR1974001 SRR5598935 SRR5632054
	FDA00001482	contaminant	Se	Saintpaul	26	4816336	-	SRR2979535	2579	0.05	-	27	14	-	0			
	PNUSAS034874	contaminant	Se	Typhimurium	44	4801352	-	SRR6765816	25564	0.5	-	19	1	-	6			
	08-0004	contaminant	Se		58	4724307	-	SRR5581942	240093	5	-	3925	-	-	7			
	CFSAN046869	contaminant	Lm		8	2883669	-	SRR3223762	-	-	-	-	-	-	-	-	-	
	CFSAN079547	contaminant	Ec	O169/O183:H18	32	4744261	-	SRR7135396	-	-	-	-	-	-	-	-	-	
Ec1	2013C-4361	subject/contaminant	Ec	O111:H8	2	5388459	GCF_003017975.1	SRR3189185	-	-	SRR2481237	1792	29	-	-	1	0	SRR1573233 SRR2178078 SRR2481237 SRR2549982 SRR2549986 SRR2549988 SRR2569425 SRR2982239 SRR3068682 SRR3113877 SRR3146221 SRR3176145 SRR5124204 SRR6181683
	PNUSAE013798	contaminant	Ec	O111:H8	180	5210940	-	SRR7133502	2644	0.05	-	16	29	-	0			
	MOD1-EC3606	contaminant	Ec	O134:H38	73	4709925	-	SRR5181518	31147	0.6	-	154	-	-	5			
	155580	contaminant	Ec	O10:H52	130	4828021	-	SRR3578956	273999	5	-	2371	-	-	7			
	CFSAN008070	contaminant	Lm		8	2857252	-	SRR1220810	-	-	-	-	-	-	-	-	-	
	2015AM-3113	contaminant	Se	Javiana	14	4626163	-	SRR6505947	-	-	-	-	-	-	-	-	-	
Ec2	C227-11	subject/contaminant	Ec	O104:H4	8	5446006	GCF_000986765.1	-	-	-	SRR341554	678	-	-	-	1	0	SRR3224368 SRR3228745 SRR341554 SRR341559 SRR341563 SRR341565 SRR341567 SRR341569 SRR341571 SRR402958 SRR402962 SRR497015 SRR497016 SRR7179943 SRR7283764
	254603	contaminant	Ec	O104:H4	135	5249416	-	SRR6467535	2725	0.05	-	678	-	-	0			
	CFSAN041118	contaminant	Ec	O185:H7	88	4903188	-	SRR3438053	27298	0.5	-	2387	-	-	5			
	NC_CF252	contaminant	Ec	O68	109	5166183	-	SRR7299301	224140	4	-	8332	-	-	7			
	PNUSAL001781	contaminant	Lm	1/2a	7	2929524	-	SRR2646874	-	-	-	-	-	-	-	-	-	
	2015AM-2276	contaminant	Se	Heidelberg	14	4754560	-	SRR6481044	-	-	-	-	-	-	-	-	-	
Ec3	FDAARGOS_402	subject/contaminant	Ec	O104:H4	5	5288218	GCF_002554485.1	SRR5878839	-	-	SRR341554	678	-	-	-	1	0	SRR3178036 SRR3224368 SRR3228745 SRR341554 SRR341559 SRR341563 SRR341565 SRR341567 SRR341569 SRR341571 SRR3578650 SRR3578911 SRR402958 SRR402962 SRR497015 SRR497016 SRR7179943 SRR7283764
	PNUSAE005897	contaminant	Ec	O181:H4	188	5357679	-	SRR5283470	2630	0.05	-	678	-	-	0			
	CFSAN074773	contaminant	Ec	O113:H7	58	4981219	-	SRR6930597	26555	0.5	-	1056	-	-	5			
	NC_CF202	contaminant	Ec	O120	119	4972798	-	SRR7297675	224415	4	-	8331	-	-	7			
	PNUSAL000947	contaminant	Lm	4b	8	2860030	-	SRR1575064	-	-	-	-	-	-	-	-	-	
	PNUSAS040943	contaminant	Se	Oranienburg	14	4563849	-	SRR7253178	-	-	-	-	-	-	-	-	-	
Ec4	FDAARGOS_348	subject/contaminant	Ec	O104:H4	1	5442454	GCF_002209105.2	SRR5427930	-	-	SRR3228745	678	-	-	-	1	0	SRR3228745 SRR341554 SRR341557 SRR341559 SRR402960 SRR402963 SRR570184 SRR570194 SRR576622 SRR576623 SRR576624 SRR576626 SRR576628 SRR576629
	535309	contaminant	Ec	O127:H4	153	5064014	-	SRR7251472	2770	0.05	-	678	-	-	0			
	CAV1494	contaminant	Ec	O9:H7	56	4677613	-	SRR2965629	27306	0.5	-	1727	446	-	6			
	195605	contaminant	Ec		84	4446514	-	SRR5035023	317583	6	-	5463	-	-	7			
	PNUSAL000889	contaminant	Lm	4c	8	2810735	-	SRR1562093	-	-	-	-	-	-	-	-	-	
	PNUSAS038297	contaminant	Se	Saintpaul	14	4693004	-	SRR6984437	-	-	-	-	-	-	-	-	-	
Ec5	2015C-4944	subject/contaminant	Ec	O26:H11	2	5901472	GCF_003018035.1	SRR3158400	-	-	SRR2982138	21	29	-	-	1	0	SRR2155441 SRR2417072 SRR2982138 SRR3829545 SRR4289045 SRR4300027 SRR4430012 SRR4430013 SRR4896243 SRR5001052 SRR5282003 SRR5312218 SRR5790143 SRR5939814 SRR6164726
	PNUSAE014345	contaminant	Ec	O103:H11	205	5612893	-	SRR7244127	2904	0.05	-	723	29	-	1			
	OH-16-24350-2	contaminant	Ec	O175:H28	47	4691267	-	SRR7130338	32433	0.5	-	200	40	-	6			
	PSU-0678	contaminant	Ec	O19:H33	28	4593555	-	SRR7085315	306512	5	-	8241	-	-	7			
	PNUSAL002166	contaminant	Lm	4c	7	2869601	-	SRR3535390	-	-	-	-	-	-	-	-	-	
	PNUSAS038663	contaminant	Se	Enteritidis	14	4820202	-	SRR7001455	-	-	-	-	-	-	-	-	-	
Ec6	2012C-4606	subject/contaminant	Ec	O26:H11	3	5725796	GCF_003018795.1	SRR3178026	-	-	SRR3178026	21	29	-	-	1	0	SRR2982191 SRR2990059 SRR2990060 SRR2990064 SRR3113895 SRR3113896 SRR3178026 SRR3340424 SRR3371771 SRR3372209 SRR3536638 SRR4054746 SRR4054752 SRR5892838 SRR5944912 SRR6359265 SRR7413759
	PNUSAE014137	contaminant	Ec	O26:H11	195	5411152	-	SRR7195070	2928	0.05	-	21	29	-	0			
	PSU-0502	contaminant	Ec	O175:H16	67	4806425	-	SRR6930654	32280	0.6	-	603	-	-	5			
	CDPHFDLB-F06M00948-9	contaminant	Ec		167	5565959	-	SRR7221692	300612	5	-	-	-	-	7			
	PNUSAL000466	contaminant	Lm	1/2a	8	2864497	-	SRR1055859	-	-	-	-	-	-	-	-	-	
	CFSAN003973	contaminant	Se	Anatum	14	4709867	-	SRR975400	-	-	-	-	-	-	-	-	-	
Ec7	9000	subject/contaminant	Ec	O157:H7	2	5611726	GCF_001890285.1	-	-	-	SRR1984111	11	11	-	-	2	0	SRR1984021 SRR1984023 SRR1984027 SRR1984111 SRR2033964 SRR2034005 SRR3579359 SRR6052027 SRR6052048 SRR6052065 SRR7191695
	XH14653V	contaminant	Ec	O157:H7	134	5292330	-	SRR2187165	2697	0.05	-	11	11	-				
	MOD1-EC5420	contaminant	Ec	O55:H7	240	5347735	-	SRR6512620	14828	0.3	-	335	11	-	1			
	0000-0054-1706	contaminant	Ec		97	5246077	-	SRR2449168	305905	5	-	-	-	-	7			
	PNUSAL000777	contaminant	Lm		8	2870376	-	SRR1506599	-	-	-	-	-	-	-	-	-	
	2015AM-1828	contaminant	Se	Newport	15	4670779	-	SRR5725237	-	-	-	-	-	-	-	-	-	
Ec8	RM8426	subject/contaminant	Ec	O26:H11	2	5738300	GCF_003112185.1	-	-	-	SRR3372209	21	29	-	-	1	0	SRR2982191 SRR2982201 SRR2990059 SRR2990060 SRR2990063 SRR2990064 SRR3113895 SRR3113896 SRR3372209 SRR4054752 SRR5042901 SRR5057539 SRR5944912
	PNUSAE014260	contaminant	Ec	O103:H11	199	5479395	-	SRR7280413	2713	0.05	-	723	29	-	1			
	MOD1-EC3606	contaminant	Ec	O134:H38	73	4709925	-	SRR5181518	32223	0.6	-	154	-	-	6			
	SN2N-1	contaminant	Ec		38	4413523	-	SRR2000282	317190	6	-	3727	-	-	7			
	PNUSAL000636	contaminant	Lm	4c	8	2833549	-	SRR1217490	-	-	-	-	-	-	-	-	-	
	2015AM-3219	contaminant	Se	Hannover	15	4828553	-	SRR6505965	-	-	-	-	-	-	-	-	-	

Note: Data are shown for *Listeria monocytogenes* (Lm), *Salmonella enterica* (Se), and *Escherichia coli* (Ec). Serotype information was obtained from BioSample entries at the National Center for Biotechnology Information whenever possible. Otherwise, serotypes for Se and Ec were estimated *in silico*.

Table S2: Results of SNP pipeline and core-genome multi locus sequence typing analyses.

	Subject	Contaminant	Contaminant Type	Percent Contamination	SNPs	Bootstrap Support	Percent Reads Mapped	Alleles	Missing Alleles	Partial Alleles	Missing and Partial Alleles	Length	Number Contigs	N50
Lm1	GCF_001952775.1	GCF_001952775.1	Self	10	5.00	0.88	100.00	2.00	0.00	3.00	3.00	3086315.00	20.00	263503.00
Lm2	GCF_001548585.1	GCF_001548585.1	Self	10	1.00	0.82	99.93	2.00	0.00	3.00	3.00	3060497.00	33.00	248908.00
Lm3	GCF_001594305.1	GCF_001594305.1	Self	10	3.00	0.93	100.00	1.00	0.00	1.00	1.00	2926129.00	16.00	437366.00
Lm4	GCF_000195435.3	GCF_000195435.3	Self	10	3.00	0.84	100.00	3.00	0.00	0.00	0.00	2921208.00	20.00	257300.00
Lm5	GCF_000438585.1	GCF_000438585.1	Self	10	1.00	0.94	100.00	1.00	0.00	0.00	0.00	3043890.00	39.00	260672.00
Lm6	GCF_000438705.2	GCF_000438705.2	Self	10	0.00	1.00	100.00	2.00	0.00	0.00	0.00	2974317.00	23.00	515362.00
Lm7	GCF_000438605.1	GCF_000438605.1	Self	10	0.00	1.00	100.00	1.00	0.00	0.00	0.00	2957878.00	25.00	307885.00
Lm8	GCF_001548635.1	GCF_001548635.1	Self	10	1.00	0.62	100.00	1.00	0.00	0.00	0.00	3007281.00	25.00	282325.00
Median			Self	10	1.00	0.91	100.00	1.50	0.00	0.00	0.00	2990799.00	24.00	272914.00
Minimum			Self	10	0.00	0.62	99.93	1.00			0.00	2921208.00	16.00	248908.00
Maximum			Self	10	5.00	1.00	100.00	3.00			3.00	3086315.00	39.00	515362.00
Lm1	GCF_001952775.1	GCF_001952775.1	Self	20	5.00	0.86	100.00	2.00	0.00	3.00	3.00	3084854.00	24.00	212624.00
Lm2	GCF_001548585.1	GCF_001548585.1	Self	20	1.00	0.88	99.93	2.00	0.00	2.00	2.00	3061312.00	37.00	199983.00
Lm3	GCF_001594305.1	GCF_001594305.1	Self	20	3.00	0.99	100.00	2.00	0.00	1.00	1.00	2926455.00	22.00	286137.00
Lm4	GCF_000195435.3	GCF_000195435.3	Self	20	3.00	0.84	100.00	3.00	0.00	0.00	0.00	2923176.00	22.00	275827.00
Lm5	GCF_000438585.1	GCF_000438585.1	Self	20	1.00	0.94	100.00	4.00	0.00	0.00	0.00	3045040.00	37.00	260672.00
Lm6	GCF_000438705.2	GCF_000438705.2	Self	20	0.00	1.00	100.00	3.00	0.00	0.00	0.00	2973689.00	26.00	515362.00
Lm7	GCF_000438605.1	GCF_000438605.1	Self	20	0.00	1.00	100.00	2.00	0.00	0.00	0.00	2957611.00	31.00	288604.00
Lm8	GCF_001548635.1	GCF_001548635.1	Self	20	1.00	0.51	100.00	1.00	1.00	0.00	1.00	3007246.00	34.00	236472.00
Median			Self	20	1.00	0.91	100.00	2.00	0.00	0.00	0.50	2990467.50	28.50	268249.50
Minimum			Self	20	0.00	0.51	99.93	1.00			0.00	2923176.00	22.00	199983.00
Maximum			Self	20	5.00	1.00	100.00	4.00			3.00	3084854.00	37.00	515362.00
Lm1	GCF_001952775.1	GCF_001952775.1	Self	30	5.00	0.89	100.00	6.00	1.00	3.00	4.00	3085481.00	26.00	184916.00
Lm2	GCF_001548585.1	GCF_001548585.1	Self	30	1.00	0.82	99.92	6.00	0.00	3.00	3.00	3061359.00	44.00	133804.00
Lm3	GCF_001594305.1	GCF_001594305.1	Self	30	3.00	0.97	100.00	3.00	1.00	0.00	1.00	2925601.00	28.00	376826.00
Lm4	GCF_000195435.3	GCF_000195435.3	Self	30	3.00	0.91	100.00	3.00	0.00	0.00	0.00	2923221.00	25.00	334913.00
Lm5	GCF_000438585.1	GCF_000438585.1	Self	30	1.00	0.93	100.00	1.00	1.00	2.00	3.00	3042362.00	41.00	245023.00
Lm6	GCF_000438705.2	GCF_000438705.2	Self	30	0.00	1.00	100.00	3.00	2.00	1.00	3.00	2974800.00	40.00	169735.00
Lm7	GCF_000438605.1	GCF_000438605.1	Self	30	0.00	1.00	100.00	1.00	2.00	2.00	4.00	2957015.00	37.00	163089.00
Lm8	GCF_001548635.1	GCF_001548635.1	Self	30	1.00	0.60	100.00	1.00	0.00	1.00	1.00	3008143.00	30.00	264561.00
Median			Self	30	1.00	0.92	100.00	3.00	1.00	1.50	3.00	2991471.50	33.50	214969.50
Minimum			Self	30	0.00	0.60	99.92	1.00			0.00	2923221.00	25.00	133804.00
Maximum			Self	30	5.00	1.00	100.00	6.00			4.00	3085481.00	44.00	376826.00
Lm1	GCF_001952775.1	GCF_001952775.1	Self	40	5.00	0.85	100.00	4.00	3.00	5.00	8.00	3084162.00	38.00	149125.00
Lm2	GCF_001548585.1	GCF_001548585.1	Self	40	1.00	0.88	99.93	5.00	1.00	2.00	3.00	3059260.00	44.00	175575.00
Lm3	GCF_001594305.1	GCF_001594305.1	Self	40	3.00	0.97	100.00	2.00	4.00	1.00	5.00	2926473.00	30.00	244646.00
Lm4	GCF_000195435.3	GCF_000195435.3	Self	40	3.00	0.85	100.00	4.00	0.00	0.00	0.00	2920858.00	30.00	224307.00
Lm5	GCF_000438585.1	GCF_000438585.1	Self	40	1.00	0.95	100.00	5.00	2.00	0.00	2.00	3043117.00	48.00	229856.00
Lm6	GCF_000438705.2	GCF_000438705.2	Self	40	0.00	1.00	100.00	3.00	1.00	2.00	3.00	2974938.00	41.00	169955.00
Lm7	GCF_000438605.1	GCF_000438605.1	Self	40	0.00	1.00	100.00	3.00	2.00	0.00	2.00	2957251.00	38.00	124245.00
Lm8	GCF_001548635.1	GCF_001548635.1	Self	40	1.00	0.66	100.00	0.00	0.00	0.00	0.00	3006516.00	37.00	200072.00
Median			Self	40	1.00	0.92	100.00	3.50	1.50	0.50	2.50	2990727.00	38.00	187823.50
Minimum			Self	40	0.00	0.66	99.93	0.00			0.00	2920858.00	30.00	124245.00
Maximum			Self	40	5.00	1.00	100.00	5.00			8.00	3084162.00	48.00	244646.00
Lm1	GCF_001952775.1	GCF_001952775.1	Self	50	5.00	0.93	100.00	8.00	0.00	5.00	5.00	3083309.00	35.00	172596.00
Lm2	GCF_001548585.1	GCF_001548585.1	Self	50	1.00	0.85	99.93	4.00	1.00	2.00	3.00	3063820.00	51.00	173814.00
Lm3	GCF_001594305.1	GCF_001594305.1	Self	50	3.00	0.97	100.00	5.00	1.00	2.00	3.00	2925038.00	30.00	206669.00
Lm4	GCF_000195435.3	GCF_000195435.3	Self	50	3.00	0.88	100.00	6.00	0.00	1.00	1.00	2921164.00	27.00	231469.00
Lm5	GCF_000438585.1	GCF_000438585.1	Self	50	1.00	0.88	100.00	3.00	3.00	0.00	3.00	3044071.00	50.00	210324.00
Lm6	GCF_000438705.2	GCF_000438705.2	Self	50	0.00	1.00	100.00	5.00	1.00	1.00	2.00	2974297.00	34.00	255155.00
Lm7	GCF_000438605.1	GCF_000438605.1	Self	50	0.00	1.00	100.00	3.00	1.00	2.00	3.00	2957087.00	38.00	131657.00
Lm8	GCF_001548635.1	GCF_001548635.1	Self	50	1.00	0.58	100.00	5.00	1.00	0.00	1.00	3006729.00	38.00	184386.00
Median			Self	50	1.00	0.91	100.00	5.00	1.00	1.50	3.00	2990513.00	36.50	195527.50
Minimum			Self	50	0.00	0.58	99.93	3.00			1.00	2921164.00	27.00	131657.00
Maximum			Self	50	5.00	1.00	100.00	8.00			5.00	3083309.00	51.00	255155.00
Lm1	GCF_001952775.1	SRR5817942	0.05	10	5.00	0.92	99.92	0.00	0.00	3.00	3.00	3092661.00	29.00	406125.00
Lm2	GCF_001548585.1	SRR5526104	0.05	10	1.00	0.88	99.51	0.00	0.00	2.00	2.00	3111021.00	95.00	260978.00
Lm3	GCF_001594305.1	SRR1998940	0.05	10	3.00	0.92	99.42	1.00	0.00	0.00	0.00	3000600.00	109.00	437366.00
Lm4	GCF_000195435.3	SRR7442117	0.05	10	3.00	0.89	99.87	3.00	0.00	0.00	0.00	2937979.00	38.00	257300.00
Lm5	GCF_000438585.1	SRR7292620	0.05	10	1.00	0.94	99.65	1.00	0.00	0.00	0.00	3086388.00	88.00	260672.00
Lm6	GCF_000438705.2	SRR339497	0.05	10	0.00	1.00	99.92	2.00	1.00	0.00	1.00	2983782.00	35.00	387936.00
Lm7	GCF_000438605.1	SRR5087886	0.05	10	0.00	1.00	99.80	0.00	0.00	0.00	0.00	2982063.00	50.00	386853.00
Lm8	GCF_001548635.1	SRR6238397	0.05	10	1.00	0.57	99.52	0.00	0.00	0.00	0.00	3067297.00	94.00	282325.00
Median			0.05	10	1.00	0.92	99.73	0.50	0.00	0.00	0.00	3033948.50	69.00	334589.00
Minimum			0.05	10	0.00	0.57	99.42	0.00			0.00	2937979.00	29.00	257300.00
Maximum			0.05	10	5.00	1.00	99.92	3.00			3.00	3111021.00	109.00	437366.00
Lm1	GCF_001952775.1	SRR5817942	0.05	20	5.00	0.82	99.85	0.00	0.00	1.00	1.00	3100003.00	40.00	229028.00
Lm2	GCF_001548585.1	SRR5526104	0.05	20	1.00	0.85	99.07	1.00	1.00	2.00	3.00	3167115.00	123.00	155591.00
Lm3	GCF_001594305.1	SRR1998940	0.05	20	3.00	0.97	98.86	1.00	1.00	3.00	4.00	3055992.00	128.00	166243.00
Lm4	GCF_000195435.3	SRR7442117	0.05	20	3.00	0.88	99.76	3.00	0.00	5.00	5.00	2953427.00	85.00	84105.00
Lm5	GCF_000438585.1	SRR7292620	0.05	20	1.00	0.94	99.34	3.00	0.00	3.00	3.00	3124327.00	112.00	182423.00
Lm6	GCF_000438705.2	SRR339497	0.05	20	0.00	1.00	99.82	3.00	0.00	0.00	0.00	2990661.00	48.00	257257.00
Lm7	GCF_000438605.1	SRR5087886	0.05	20	0.00	1.00	99.61	1.00	0.00	1.00	1.00	3001746.00	68.00	200983.00
Lm8	GCF_001548635.1	SRR6238397	0.05	20	1.00	0.63	99.06	2.00	0.00	1.00	1.00	3122386.00	111.00	253846.00
Median			0.05	20	1.00	0.91	99.48	1.50	0.00	1.50	2.00	3077997.50	98.00	191703.00
Minimum			0.05	20	0.00	0.63	98.86	0.00			0.00	2953427.00	40.00	84105.00
Maximum			0.05	20	5.00	1.00	99.85	3.00			5.00	3167115.00	128.00	257257.00
Lm1	GCF_001952775.1	SRR5817942	0.05	30	5.00	0.79	99.77	2.00	0.00	4.00	4.00	3110181.00	78.00	135769.00
Lm2	GCF_001548585.1	SRR5526104	0.05	30	1.00	0.62	98.67	10.00	5.00	4.00	9.00	3182264.00	131.00	105224.00
Lm3	GCF_001594305.1	SRR1998940	0.05	30	2.00	0.94	98.38	5.00	2.00	7.00	9.00	3079332.00	111.00	95036.00
Lm4	GCF_000195435.3	SRR7442117	0.05	30	3.00	0.89	99.66	8.00	3.00	4.00	7.00	2957678.00	123.00	49834.00
Lm5	GCF_000438585.1	SRR7292620	0.05	30	0.00	0.92	98.92	7.00	0.00	5.00	5.00	3144791.00	106.00	154475.00
Lm6	GCF_000438705.2	SRR339497	0.05	30	1.00	1.00	99.77	4.00	2.00	2.00	4.00	2994938.00	90.00	102385.00
Lm7	GCF_000438605.1	SRR5087886	0.05	30	0.00	1.00	99.41	0.00	2.00	3.00	5.00	3013581.00	74.00	108456.00
Lm8	GCF_001548635.1	SRR6238397	0.05	30	1.00	0.65	98.56	7.00	3.00	8.00	11.00	3143253.00	120.00	76503.00
Median			0.05	30	1.00	0.91	99.17	6.00	2.00	4.00	6.00	3094756.50	108.50	103804.50
Minimum			0.05	30	0.00	0.62	98.38	0.00			4.00	2957678.00	74.00	49834.00
Maximum			0.05	30	5.00	1.00	99.77	10.00			11.00	3182264.00	131.00	154475.00
Lm1	GCF_001952775.1	SRR5817942	0.05	40	6.00	0.79	99.70	3.00	2.00	12.00	14.00	3109617.00	100.00	89869.00
Lm2	GCF_001548585.1	SRR5526104	0.05	40	3.00	0.50	98.21	20.00	5.00	10.00	15.00	3192662.00	196.00	37277.00
Lm3	GCF_001594305.1	SRR1998940	0.05	40	5.00	0.63	97.74	12.00	6.00	16.00	22.00	3089176.00	142.00	52252.00
Lm4	GCF_000195435.3	SRR7442117	0.05	40	3.00	0.90	99.49	14.00	7.00	13.00	20.00	2962442.00	236.00	21853.00
Lm5	GCF_000438585.1	SRR7292620	0.05	40	0.00	0.94	98.57	9.00	3.00	8.00	11.00	3147598.00	121.00	76657.00
Lm6	GCF_000438705.2	SRR339497	0.05	40	3.00	1.00	99.71	10.00	4.00	4.00	8.00	3003161.00	113.00	69045.00
Lm7	GCF_000438605.1	SRR5087886	0.05	40	1.00	1.00	99.23	7.00	3.00	1.00	4.00	3016535.00	81.00	84415.00
Lm8	GCF_001548635.1	SRR6238397	0.05	40	1.00	0.67	98.07	11.00	7.00	9.00	16.00	3148569.00	189.00	37408.00
Median			0.05	40	3.00	0.85	98.90	10.50	4.50	9.50	14.50	3099396.50	131.50	60648.50
Minimum			0.05	40	0.00	0.50	97.74	3.00			4.00	2962442.00	81.00	21853.00
Maximum			0.05	40	6.00	1.00	99.71	20.00			22.00	3192662.00	236.00	89869.00
Lm1	GCF_001952775.1	SRR5817942	0.05	50	9.00	0.00	99.62	12.00	1.00	13.00	14.00	3110130.00	111.00	91387.00
Lm2	GCF_001548585.1	SRR5526104	0.05	50	4.00	0.66	97.74	29.00	7.00	10.00	17.00	3197935.00	237.00	24675.00
Lm3	GCF_001594305.1	SRR1998940	0.05	50	2.00	0.69	97.18	38.00	4.00	11.00	15.00	3091690.00	134.00	39587.00
Lm4	GCF_000195435.3	SRR7442117	0.05	50	2.00	0.88	99.37	17.00	9.00	16.00	25.00	2958676.00	301.00	17144.00
Lm5	GCF_000438585.1	SRR7292620	0.05	50	0.00	0.87	98.21	20.00	2.00	16.00	18.00	3152629.00	166.00	43120.00
Lm6	GCF_000438705.2	SRR339497	0.05	50	8.00	1.00	99.63	15.00	5.00	11.00	16.00	2999803.00	152.00	48765.00
Lm7	GCF_000438605.1	SRR5087886	0.05	50	4.00	1.00	99.06	14.00	3.00	5.00	8.00	3018294.00	108.00	61061.00
Lm8	GCF_001548635.1	SRR6238397	0.05	50	2.00	0.00	97.62	28.00	7.00	15.00	22.00	3152107.00	208.00	26279.00
Median			0.05	50	3.00	0.78	98.64	18.50	4.50	12.00	16.50	3100910.00	159.00	41353.50
Minimum			0.05	50	0.00	0.00	97.18	12.00			8.00	2958676.00	108.00	17144.00
Maximum			0.05	50	9.00	1.00	99.63	38.00			25.00	3197935.00	301.00	91387.00
Lm1	GCF_001952775.1	ERR1276239	0.5	10	5.00	0.96	99.77	0.00	0.00	1.00	1.00	3109805.00	57.00	217278.00
Lm2	GCF_001548585.1	SRR1016606	0.5	10	1.00	0.90	99.55	3.00	0.00	2.00	2.00	3104590.00	92.00	154971.00
Lm3	GCF_001594305.1	SRR1695819	0.5	10	3.00	0.96	99.82	3.00	0.00	0.00	0.00	2944100.00	41.00	299560.00
Lm4	GCF_000195435.3	SRR1998941	0.5	10	2.00	0.87	99.06	4.00	1.00	0.00	1.00	3035088.00	164.00	334953.00
Lm5	GCF_000438585.1	SRR1745479	0.5	10	1.00	0.91	99.61	3.00	2.00	1.00	3.00	3088554.00	105.00	166061.00
Lm6	GCF_000438705.2	SRR5571267	0.5	10	0.00	1.00	99.21	3.00	1.00	1.00	2.00	3070078.00	149.00	335002.00
Lm7	GCF_000438605.1	SRR2673275	0.5	10	0.00	1.00	99.76	2.00	0.00	2.00	2.00	2989788.00	65.00	240609.00
Lm8	GCF_001548635.1	SRR3930187	0.5	10	1.00	0.60	99.52	1.00	0.00	0.00	0.00	3055460.00	83.00	301894.00
Median			0.5	10	1.00	0.94	99.58	3.00	0.00	1.00	1.50	3062769.00	87.50	270084.50
Minimum			0.5	10	0.00	0.60	99.06	0.00			0.00	2944100.00	41.00	154971.00
Maximum			0.5	10	5.00	1.00	99.82	4.00			3.00	3109805.00	164.00	335002.00
Lm1	GCF_001952775.1	ERR1276239	0.5	20	5.00	0.89	99.55	17.00	9.00	27.00	36.00	3128584.00	221.00	28570.00
Lm2	GCF_001548585.1	SRR1016606	0.5	20	1.00	0.84	99.20	24.00	21.00	25.00	46.00	3136387.00	303.00	22757.00
Lm3	GCF_001594305.1	SRR1695819	0.5	20	3.00	0.94	99.64	16.00	10.00	16.00	26.00	2957783.00	150.00	53945.00
Lm4	GCF_000195435.3	SRR1998941	0.5	20	2.00	0.97	98.09	31.00	9.00	13.00	22.00	3130594.00	260.00	58982.00
Lm5	GCF_000438585.1	SRR1745479	0.5	20	1.00	0.94	99.17	22.00	27.00	39.00	66.00	3132830.00	324.00	23762.00
Lm6	GCF_000438705.2	SRR5571267	0.5	20	0.00	1.00	98.43	27.00	15.00	20.00	35.00	3150293.00	330.00	27812.00
Lm7	GCF_000438605.1	SRR2673275	0.5	20	0.00	1.00	99.54	13.00	5.00	13.00	18.00	3011368.00	158.00	51527.00
Lm8	GCF_001548635.1	SRR3930187	0.5	20	1.00	0.66	99.03	40.00	15.00	27.00	42.00	3110592.00	288.00	22917.00
Median			0.5	20	1.00	0.94	99.19	23.00	12.50	22.50	35.50	3129589.00	274.00	28191.00
Minimum			0.5	20	0.00	0.66	98.09	13.00			18.00	2957783.00	150.00	22757.00
Maximum			0.5	20	5.00	1.00	99.64	40.00			66.00	3150293.00	330.00	58982.00
Lm1	GCF_001952775.1	ERR1276239	0.5	30	5.00	0.81	99.33	77.00	38.00	75.00	113.00	3133073.00	536.00	9702.00
Lm2	GCF_001548585.1	SRR1016606	0.5	30	3.00	0.65	98.87	96.00	48.00	112.00	160.00	3133954.00	711.00	7133.00
Lm3	GCF_001594305.1	SRR1695819	0.5	30	3.00	0.95	99.48	64.00	21.00	41.00	62.00	2960700.00	338.00	22339.00
Lm4	GCF_000195435.3	SRR1998941	0.5	30	3.00	0.82	97.14	120.00	42.00	72.00	114.00	3166668.00	687.00	7693.00
Lm5	GCF_000438585.1	SRR1745479	0.5	30	0.00	0.94	98.76	103.00	57.00	107.00	164.00	3132804.00	726.00	6991.00
Lm6	GCF_000438705.2	SRR5571267	0.5	30	0.00	1.00	97.57	96.00	51.00	95.00	146.00	3170719.00	693.00	7685.00
Lm7	GCF_000438605.1	SRR2673275	0.5	30	0.00	1.00	99.30	51.00	21.00	31.00	52.00	3009076.00	295.00	28120.00
Lm8	GCF_001548635.1	SRR3930187	0.5	30	1.00	0.63	98.56	112.00	47.00	83.00	130.00	3111498.00	681.00	7474.00
Median			0.5	30	2.00	0.88	98.82	96.00	44.50	79.00	122.00	3132938.50	684.00	7689.00
Minimum			0.5	30	0.00	0.63	97.14	51.00			52.00	2960700.00	295.00	6991.00
Maximum			0.5	30	5.00	1.00	99.48	120.00			164.00	3170719.00	726.00	28120.00
Lm1	GCF_001952775.1	ERR1276239	0.5	40	7.00	0.56	99.09	138.00	79.00	174.00	253.00	3088029.00	1116.00	3807.00
Lm2	GCF_001548585.1	SRR1016606	0.5	40	7.00	0.00	98.56	165.00	106.00	213.00	319.00	3060534.00	1367.00	3151.00
Lm3	GCF_001594305.1	SRR1695819	0.5	40	3.00	0.95	99.30	109.00	47.00	83.00	130.00	2951278.00	559.00	15912.00
Lm4	GCF_000195435.3	SRR1998941	0.5	40	5.00	0.81	96.26	184.00	111.00	210.00	321.00	3099392.00	1328.00	3222.00
Lm5	GCF_000438585.1	SRR1745479	0.5	40	4.00	0.94	98.35	168.00	121.00	230.00	351.00	3056154.00	1399.00	3097.00
Lm6	GCF_000438705.2	SRR5571267	0.5	40	6.00	1.00	96.77	172.00	105.00	219.00	324.00	3101361.00	1333.00	3275.00
Lm7	GCF_000438605.1	SRR2673275	0.5	40	0.00	1.00	99.05	88.00	38.00	90.00	128.00	3002134.00	644.00	14096.00
Lm8	GCF_001548635.1	SRR3930187	0.5	40	2.00	0.74	98.14	188.00	101.00	228.00	329.00	3044929.00	1382.00	3134.00
Median			0.5	40	4.50	0.88	98.46	166.50	103.00	211.50	320.00	3058344.00	1330.50	3248.50
Minimum			0.5	40	0.00	0.00	96.26	88.00			128.00	2951278.00	559.00	3097.00
Maximum			0.5	40	7.00	1.00	99.30	188.00			351.00	3101361.00	1399.00	15912.00
Lm1	GCF_001952775.1	ERR1276239	0.5	50	27.00	0.00	98.88	235.00	78.00	209.00	287.00	3076816.00	1222.00	3483.00
Lm2	GCF_001548585.1	SRR1016606	0.5	50	31.00	0.00	98.19	287.00	133.00	251.00	384.00	3040517.00	1511.00	2805.00
Lm3	GCF_001594305.1	SRR1695819	0.5	50	28.00	0.53	99.15	162.00	59.00	115.00	174.00	2923134.00	753.00	11190.00
Lm4	GCF_000195435.3	SRR1998941	0.5	50	17.00	0.72	95.18	308.00	119.00	241.00	360.00	3082490.00	1500.00	2788.00
Lm5	GCF_000438585.1	SRR1745479	0.5	50	19.00	0.80	97.96	301.00	111.00	281.00	392.00	3044327.00	1539.00	2662.00
Lm6	GCF_000438705.2	SRR5571267	0.5	50	28.00	0.98	95.99	313.00	126.00	231.00	357.00	3078361.00	1509.00	2738.00
Lm7	GCF_000438605.1	SRR2673275	0.5	50	16.00	1.00	98.86	147.00	48.00	104.00	152.00	2999619.00	717.00	12629.00
Lm8	GCF_001548635.1	SRR3930187	0.5	50	16.00	0.52	97.66	308.00	109.00	256.00	365.00	3033267.00	1481.00	2944.00
Median			0.5	50	23.00	0.63	98.08	294.00	110.00	236.00	358.50	3042422.00	1490.50	2874.50
Minimum			0.5	50	16.00	0.00	95.18	147.00			152.00	2923134.00	717.00	2662.00
Maximum			0.5	50	31.00	1.00	99.15	313.00			392.00	3082490.00	1539.00	12629.00
Lm1	GCF_001952775.1	SRR5931817	5	10	5.00	0.93	97.36	15.00	1.00	5.00	6.00	3335453.00	363.00	144162.00
Lm2	GCF_001548585.1	SRR6957404	5	10	1.00	0.88	97.35	18.00	0.00	4.00	4.00	3334898.00	391.00	120453.00
Lm3	GCF_001594305.1	SRR3405537	5	10	0.00	0.98	98.19	18.00	0.00	1.00	1.00	3079252.00	226.00	330092.00
Lm4	GCF_000195435.3	SRR3747686	5	10	2.00	0.96	97.88	16.00	0.00	0.00	0.00	3127011.00	294.00	257300.00
Lm5	GCF_000438585.1	SRR6957404	5	10	1.00	0.91	97.47	17.00	1.00	2.00	3.00	3286475.00	354.00	210324.00
Lm6	GCF_000438705.2	SRR7073271	5	10	0.00	1.00	98.05	21.00	0.00	0.00	0.00	3144950.00	250.00	255155.00
Lm7	GCF_000438605.1	SRR5205362	5	10	0.00	1.00	98.17	14.00	0.00	1.00	1.00	3124313.00	245.00	299952.00
Lm8	GCF_001548635.1	SRR975368	5	10	1.00	0.65	98.30	17.00	0.00	0.00	0.00	3153379.00	224.00	259753.00
Median			5	10	1.00	0.95	97.97	17.00	0.00	1.00	1.00	3149164.50	272.00	256227.50
Minimum			5	10	0.00	0.65	97.35	14.00			0.00	3079252.00	224.00	120453.00
Maximum			5	10	5.00	1.00	98.30	21.00			6.00	3335453.00	391.00	330092.00
Lm1	GCF_001952775.1	SRR5931817	5	20	5.00	0.98	94.43	163.00	13.00	38.00	51.00	3726024.00	808.00	17508.00
Lm2	GCF_001548585.1	SRR6957404	5	20	1.00	0.90	94.72	154.00	7.00	37.00	44.00	3676425.00	772.00	18509.00
Lm3	GCF_001594305.1	SRR3405537	5	20	0.00	0.95	96.59	161.00	9.00	19.00	28.00	3261803.00	477.00	29643.00
Lm4	GCF_000195435.3	SRR3747686	5	20	2.00	0.92	95.88	132.00	7.00	12.00	19.00	3347010.00	549.00	30278.00
Lm5	GCF_000438585.1	SRR6957404	5	20	1.00	0.94	94.80	153.00	16.00	33.00	49.00	3654346.00	788.00	16731.00
Lm6	GCF_000438705.2	SRR7073271	5	20	0.00	1.00	96.09	137.00	9.00	14.00	23.00	3384963.00	531.00	30156.00
Lm7	GCF_000438605.1	SRR5205362	5	20	0.00	1.00	96.54	138.00	7.00	18.00	25.00	3302428.00	494.00	28384.00
Lm8	GCF_001548635.1	SRR975368	5	20	1.00	0.63	96.61	146.00	9.00	26.00	35.00	3355958.00	519.00	25918.00
Median			5	20	1.00	0.95	95.99	149.50	9.00	22.50	31.50	3370460.50	540.00	27151.00
Minimum			5	20	0.00	0.63	94.43	132.00			19.00	3261803.00	477.00	16731.00
Maximum			5	20	5.00	1.00	96.61	163.00			51.00	3726024.00	808.00	30278.00
Lm1	GCF_001952775.1	SRR5931817	5	30	5.00	0.84	91.66	148.00	90.00	258.00	348.00	4301694.00	2621.00	2160.00
Lm2	GCF_001548585.1	SRR6957404	5	30	4.00	0.84	92.13	161.00	91.00	281.00	372.00	4289611.00	2732.00	1979.00
Lm3	GCF_001594305.1	SRR3405537	5	30	1.00	0.76	94.80	242.00	95.00	266.00	361.00	3561046.00	2183.00	2162.00
Lm4	GCF_000195435.3	SRR3747686	5	30	1.00	0.90	93.82	250.00	75.00	248.00	323.00	3618839.00	1997.00	2558.00
Lm5	GCF_000438585.1	SRR6957404	5	30	3.00	0.99	92.26	136.00	88.00	280.00	368.00	4299479.00	2766.00	1966.00
Lm6	GCF_000438705.2	SRR7073271	5	30	6.00	1.00	94.29	237.00	90.00	256.00	346.00	3650758.00	2144.00	2271.00
Lm7	GCF_000438605.1	SRR5205362	5	30	0.00	1.00	94.76	251.00	90.00	241.00	331.00	3601477.00	2140.00	2358.00
Lm8	GCF_001548635.1	SRR975368	5	30	1.00	0.64	94.97	232.00	91.00	292.00	383.00	3637919.00	2263.00	2132.00
Median			5	30	2.00	0.87	94.06	234.50	90.00	262.00	354.50	3644338.50	2223.00	2161.00
Minimum			5	30	0.00	0.64	91.66	136.00			323.00	3561046.00	1997.00	1966.00
Maximum			5	30	6.00	1.00	94.97	251.00			383.00	4301694.00	2766.00	2558.00
Lm1	GCF_001952775.1	SRR5931817	5	40	20.00	0.53	88.81	224.00	97.00	246.00	343.00	4702129.00	2774.00	2278.00
Lm2	GCF_001548585.1	SRR6957404	5	40	19.00	0.00	89.52	221.00	104.00	245.00	349.00	4744328.00	2738.00	2364.00
Lm3	GCF_001594305.1	SRR3405537	5	40	15.00	0.64	92.96	292.00	117.00	366.00	483.00	4048508.00	3198.00	1451.00
Lm4	GCF_000195435.3	SRR3747686	5	40	9.00	0.78	91.62	241.00	134.00	389.00	523.00	4111864.00	3279.00	1429.00
Lm5	GCF_000438585.1	SRR6957404	5	40	22.00	0.76	89.74	241.00	89.00	242.00	331.00	4704194.00	2717.00	2393.00
Lm6	GCF_000438705.2	SRR7073271	5	40	15.00	1.00	92.29	227.00	144.00	394.00	538.00	4158686.00	3293.00	1442.00
Lm7	GCF_000438605.1	SRR5205362	5	40	11.00	1.00	93.00	245.00	126.00	364.00	490.00	4100969.00	3302.00	1411.00
Lm8	GCF_001548635.1	SRR975368	5	40	20.00	0.64	93.24	238.00	118.00	409.00	527.00	4120806.00	3331.00	1425.00
Median			5	40	17.00	0.70	91.96	239.50	117.50	365.00	486.50	4139746.00	3238.50	1446.50
Minimum			5	40	9.00	0.00	88.81	221.00			331.00	4048508.00	2717.00	1411.00
Maximum			5	40	22.00	1.00	93.24	292.00			538.00	4744328.00	3331.00	2393.00
Lm1	GCF_001952775.1	SRR5931817	5	50	94.00	0.00	86.05	364.00	89.00	236.00	325.00	4785376.00	2670.00	2517.00
Lm2	GCF_001548585.1	SRR6957404	5	50	111.00	0.00	87.05	397.00	69.00	241.00	310.00	4788227.00	2624.00	2516.00
Lm3	GCF_001594305.1	SRR3405537	5	50	92.00	0.85	91.36	432.00	115.00	343.00	458.00	4231448.00	3160.00	1612.00
Lm4	GCF_000195435.3	SRR3747686	5	50	59.00	0.67	89.64	718.00	130.00	351.00	481.00	4274047.00	3247.00	1532.00
Lm5	GCF_000438585.1	SRR6957404	5	50	95.00	0.94	87.07	345.00	77.00	249.00	326.00	4756729.00	2643.00	2474.00
Lm6	GCF_000438705.2	SRR7073271	5	50	100.00	0.99	90.26	719.00	124.00	375.00	499.00	4336675.00	3242.00	1638.00
Lm7	GCF_000438605.1	SRR5205362	5	50	101.00	1.00	91.18	404.00	120.00	385.00	505.00	4224536.00	3307.00	1483.00
Lm8	GCF_001548635.1	SRR975368	5	50	82.00	0.00	91.50	742.00	107.00	361.00	468.00	4309870.00	3281.00	1551.00
Median			5	50	94.50	0.76	89.95	418.00	111.00	347.00	463.00	4323272.50	3201.00	1625.00
Minimum			5	50	59.00	0.00	86.05	345.00			310.00	4224536.00	2624.00	1483.00
Maximum			5	50	111.00	1.00	91.50	742.00			505.00	4788227.00	3307.00	2517.00
Lm1	GCF_001952775.1	SRR5944128	Se	10	5.00	0.89	90.00	0.00	0.00	3.00	3.00	4302558.00	1737.00	175279.00
Lm2	GCF_001548585.1	SRR6480957	Se	10	1.00	0.91	89.93	1.00	0.00	2.00	2.00	4280163.00	1732.00	152718.00
Lm3	GCF_001594305.1	SRR5947454	Se	10	1.00	0.96	89.95	2.00	0.00	1.00	1.00	4057856.00	1592.00	286137.00
Lm4	GCF_000195435.3	SRR5947763	Se	10	3.00	0.88	90.00	3.00	0.00	0.00	0.00	4000447.00	1561.00	223421.00
Lm5	GCF_000438585.1	SRR6480961	Se	10	1.00	0.95	90.00	1.00	1.00	0.00	1.00	4224749.00	1710.00	229738.00
Lm6	GCF_000438705.2	SRR975397	Se	10	0.00	1.00	90.00	2.00	0.00	0.00	0.00	4104208.00	1605.00	335002.00
Lm7	GCF_000438605.1	SRR5955289	Se	10	0.00	1.00	90.00	0.00	0.00	0.00	0.00	4074873.00	1594.00	228574.00
Lm8	GCF_001548635.1	SRR6480942	Se	10	1.00	0.58	90.00	2.00	0.00	0.00	0.00	4180693.00	1699.00	197758.00
Median			Se	10	1.00	0.93	90.00	1.50	0.00	0.00	0.50	4142450.50	1652.00	225997.50
Minimum			Se	10	0.00	0.58	89.93	0.00			0.00	4000447.00	1561.00	152718.00
Maximum			Se	10	5.00	1.00	90.00	3.00			3.00	4302558.00	1737.00	335002.00
Lm1	GCF_001952775.1	SRR5944128	Se	20	5.00	0.94	80.00	2.00	0.00	4.00	4.00	6013711.00	3040.00	57006.00
Lm2	GCF_001548585.1	SRR6480957	Se	20	1.00	0.80	79.94	4.00	0.00	2.00	2.00	5936358.00	3008.00	22690.00
Lm3	GCF_001594305.1	SRR5947454	Se	20	1.00	0.96	79.96	3.00	2.00	2.00	4.00	5681778.00	2963.00	29672.00
Lm4	GCF_000195435.3	SRR5947763	Se	20	3.00	0.87	80.00	3.00	0.00	2.00	2.00	5728162.00	3089.00	21553.00
Lm5	GCF_000438585.1	SRR6480961	Se	20	1.00	0.92	80.00	1.00	0.00	2.00	2.00	5947832.00	3066.00	8671.00
Lm6	GCF_000438705.2	SRR975397	Se	20	0.00	1.00	80.00	3.00	0.00	1.00	1.00	5844252.00	3065.00	12885.00
Lm7	GCF_000438605.1	SRR5955289	Se	20	0.00	1.00	80.00	3.00	1.00	0.00	1.00	5764766.00	2966.00	24906.00
Lm8	GCF_001548635.1	SRR6480942	Se	20	1.00	0.61	80.00	2.00	0.00	0.00	0.00	5859410.00	3062.00	33646.00
Median			Se	20	1.00	0.93	80.00	3.00	0.00	2.00	2.00	5851831.00	3051.00	23798.00
Minimum			Se	20	0.00	0.61	79.94	1.00			0.00	5681778.00	2963.00	8671.00
Maximum			Se	20	5.00	1.00	80.00	4.00			4.00	6013711.00	3089.00	57006.00
Lm1	GCF_001952775.1	SRR5944128	Se	30	5.00	0.93	70.00	4.00	1.00	5.00	6.00	7014016.00	2873.00	3546.00
Lm2	GCF_001548585.1	SRR6480957	Se	30	1.00	0.84	69.95	5.00	2.00	6.00	8.00	6961956.00	2769.00	3762.00
Lm3	GCF_001594305.1	SRR5947454	Se	30	1.00	0.95	69.96	4.00	1.00	5.00	6.00	6710315.00	2809.00	3435.00
Lm4	GCF_000195435.3	SRR5947763	Se	30	3.00	0.93	70.00	6.00	0.00	3.00	3.00	6754326.00	2972.00	32044.00
Lm5	GCF_000438585.1	SRR6480961	Se	30	1.00	0.92	70.00	5.00	2.00	3.00	5.00	6980776.00	2943.00	3256.00
Lm6	GCF_000438705.2	SRR975397	Se	30	0.00	1.00	70.00	6.00	1.00	4.00	5.00	6822001.00	2857.00	3279.00
Lm7	GCF_000438605.1	SRR5955289	Se	30	0.00	1.00	70.00	5.00	1.00	4.00	5.00	6782391.00	2884.00	3302.00
Lm8	GCF_001548635.1	SRR6480942	Se	30	1.00	0.64	70.00	4.00	2.00	2.00	4.00	6890936.00	2843.00	3358.00
Median			Se	30	1.00	0.93	70.00	5.00	1.00	4.00	5.00	6856468.50	2865.00	3396.50
Minimum			Se	30	0.00	0.64	69.95	4.00			3.00	6710315.00	2769.00	3256.00
Maximum			Se	30	5.00	1.00	70.00	6.00			8.00	7014016.00	2972.00	32044.00
Lm1	GCF_001952775.1	SRR5944128	Se	40	5.00	0.92	60.00	15.00	4.00	15.00	19.00	7491760.00	2138.00	5578.00
Lm2	GCF_001548585.1	SRR6480957	Se	40	1.00	0.86	59.96	14.00	5.00	10.00	15.00	7352127.00	1997.00	5794.00
Lm3	GCF_001594305.1	SRR5947454	Se	40	2.00	0.97	59.97	14.00	3.00	10.00	13.00	7193066.00	2110.00	5036.00
Lm4	GCF_000195435.3	SRR5947763	Se	40	3.00	0.89	60.00	1.00	6.00	10.00	16.00	7278613.00	2334.00	4772.00
Lm5	GCF_000438585.1	SRR6480961	Se	40	1.00	0.94	60.00	10.00	10.00	13.00	23.00	7450184.00	2208.00	5088.00
Lm6	GCF_000438705.2	SRR975397	Se	40	0.00	1.00	60.00	15.00	7.00	14.00	21.00	7335218.00	2209.00	5117.00
Lm7	GCF_000438605.1	SRR5955289	Se	40	0.00	1.00	60.00	21.00	7.00	3.00	10.00	7266877.00	2222.00	4897.00
Lm8	GCF_001548635.1	SRR6480942	Se	40	1.00	0.66	60.00	16.00	5.00	5.00	10.00	7330554.00	2143.00	5425.00
Median			Se	40	1.00	0.93	60.00	14.50	5.50	10.00	15.50	7332886.00	2175.50	5102.50
Minimum			Se	40	0.00	0.66	59.96	1.00			10.00	7193066.00	1997.00	4772.00
Maximum			Se	40	5.00	1.00	60.00	21.00			23.00	7491760.00	2334.00	5794.00
Lm1	GCF_001952775.1	SRR5944128	Se	50	5.00	0.93	50.00	34.00	9.00	26.00	35.00	7681611.00	1432.00	9106.00
Lm2	GCF_001548585.1	SRR6480957	Se	50	1.00	0.86	49.96	37.00	12.00	40.00	52.00	7502992.00	1346.00	9055.00
Lm3	GCF_001594305.1	SRR5947454	Se	50	1.00	0.96	49.98	41.00	12.00	22.00	34.00	7386804.00	1461.00	8605.00
Lm4	GCF_000195435.3	SRR5947763	Se	50	3.00	0.91	50.00	4.00	14.00	40.00	54.00	7509764.00	1657.00	7139.00
Lm5	GCF_000438585.1	SRR6480961	Se	50	1.00	0.90	50.00	42.00	17.00	34.00	51.00	7646870.00	1581.00	8267.00
Lm6	GCF_000438705.2	SRR975397	Se	50	0.00	1.00	50.00	34.00	20.00	25.00	45.00	7532164.00	1583.00	8010.00
Lm7	GCF_000438605.1	SRR5955289	Se	50	0.00	1.00	50.00	39.00	8.00	30.00	38.00	7479513.00	1550.00	7707.00
Lm8	GCF_001548635.1	SRR6480942	Se	50	1.00	0.63	50.00	44.00	13.00	30.00	43.00	7524037.00	1507.00	8292.00
Median			Se	50	1.00	0.92	50.00	38.00	12.50	30.00	44.00	7516900.50	1528.50	8279.50
Minimum			Se	50	0.00	0.63	49.96	4.00			34.00	7386804.00	1346.00	7139.00
Maximum			Se	50	5.00	1.00	50.00	44.00			54.00	7681611.00	1657.00	9106.00
Lm1	GCF_001952775.1	SRR6186591	Ec	10	5.00	0.88	90.00	0.00	0.00	3.00	3.00	4301493.00	1730.00	177092.00
Lm2	GCF_001548585.1	SRR2177969	Ec	10	1.00	0.84	89.93	2.00	0.00	1.00	1.00	4252124.00	1718.00	152758.00
Lm3	GCF_001594305.1	SRR5515964	Ec	10	1.00	0.94	89.95	1.00	0.00	0.00	0.00	3968367.00	1510.00	376802.00
Lm4	GCF_000195435.3	SRR3715600	Ec	10	3.00	0.91	90.00	2.00	0.00	0.00	0.00	4001760.00	1563.00	224307.00
Lm5	GCF_000438585.1	SRR5617247	Ec	10	1.00	0.95	90.00	1.00	0.00	0.00	0.00	4265137.00	1734.00	229738.00
Lm6	GCF_000438705.2	SRR7085315	Ec	10	0.00	1.00	90.00	2.00	0.00	0.00	0.00	4135287.00	1665.00	255158.00
Lm7	GCF_000438605.1	SRR3040406	Ec	10	0.00	1.00	90.00	1.00	1.00	0.00	1.00	4057529.00	1587.00	206541.00
Lm8	GCF_001548635.1	SRR3050978	Ec	10	1.00	0.70	90.00	0.00	0.00	0.00	0.00	4150217.00	1670.00	175948.00
Median			Ec	10	1.00	0.93	90.00	1.00	0.00	0.00	0.00	4142752.00	1667.50	215424.00
Minimum			Ec	10	0.00	0.70	89.93	0.00			0.00	3968367.00	1510.00	152758.00
Maximum			Ec	10	5.00	1.00	90.00	2.00			3.00	4301493.00	1734.00	376802.00
Lm1	GCF_001952775.1	SRR6186591	Ec	20	5.00	0.90	80.00	6.00	0.00	4.00	4.00	6003143.00	3023.00	44277.00
Lm2	GCF_001548585.1	SRR2177969	Ec	20	1.00	0.85	79.94	1.00	0.00	3.00	3.00	6007338.00	3112.00	17100.00
Lm3	GCF_001594305.1	SRR5515964	Ec	20	2.00	0.93	79.96	1.00	1.00	3.00	4.00	5685236.00	3092.00	40666.00
Lm4	GCF_000195435.3	SRR3715600	Ec	20	3.00	0.85	80.00	2.00	1.00	1.00	2.00	5697078.00	3055.00	49666.00
Lm5	GCF_000438585.1	SRR5617247	Ec	20	1.00	0.94	80.00	2.00	0.00	1.00	1.00	5958048.00	3026.00	8671.00
Lm6	GCF_000438705.2	SRR7085315	Ec	20	0.00	1.00	80.00	2.00	0.00	1.00	1.00	5746319.00	2900.00	24471.00
Lm7	GCF_000438605.1	SRR3040406	Ec	20	0.00	1.00	80.00	2.00	1.00	1.00	2.00	5798164.00	3147.00	21678.00
Lm8	GCF_001548635.1	SRR3050978	Ec	20	1.00	0.57	80.00	1.00	0.00	1.00	1.00	5923352.00	3185.00	9260.00
Median			Ec	20	1.00	0.92	80.00	2.00	0.00	1.00	2.00	5860758.00	3073.50	23074.50
Minimum			Ec	20	0.00	0.57	79.94	1.00			1.00	5685236.00	2900.00	8671.00
Maximum			Ec	20	5.00	1.00	80.00	6.00			4.00	6007338.00	3185.00	49666.00
Lm1	GCF_001952775.1	SRR6186591	Ec	30	5.00	0.92	70.00	7.00	0.00	8.00	8.00	6971025.00	2649.00	3802.00
Lm2	GCF_001548585.1	SRR2177969	Ec	30	1.00	0.85	69.95	4.00	1.00	5.00	6.00	7002275.00	2862.00	3558.00
Lm3	GCF_001594305.1	SRR5515964	Ec	30	2.00	0.98	69.96	3.00	1.00	2.00	3.00	6873271.00	3214.00	2864.00
Lm4	GCF_000195435.3	SRR3715600	Ec	30	3.00	0.91	70.00	2.00	2.00	5.00	7.00	6760933.00	3045.00	3025.00
Lm5	GCF_000438585.1	SRR5617247	Ec	30	1.00	0.92	70.00	7.00	1.00	2.00	3.00	6939633.00	2821.00	3483.00
Lm6	GCF_000438705.2	SRR7085315	Ec	30	0.00	1.00	70.00	7.00	1.00	4.00	5.00	6772687.00	2738.00	3582.00
Lm7	GCF_000438605.1	SRR3040406	Ec	30	0.00	1.00	70.00	3.00	2.00	1.00	3.00	6886763.00	3086.00	2897.00
Lm8	GCF_001548635.1	SRR3050978	Ec	30	1.00	0.64	70.00	4.00	0.00	1.00	1.00	6987377.00	3119.00	2974.00
Median			Ec	30	1.00	0.92	70.00	4.00	1.00	3.00	4.00	6913198.00	2953.50	3254.00
Minimum			Ec	30	0.00	0.64	69.95	2.00			1.00	6760933.00	2649.00	2864.00
Maximum			Ec	30	5.00	1.00	70.00	7.00			8.00	7002275.00	3214.00	3802.00
Lm1	GCF_001952775.1	SRR6186591	Ec	40	5.00	0.91	60.00	10.00	5.00	18.00	23.00	7397213.00	1949.00	6357.00
Lm2	GCF_001548585.1	SRR2177969	Ec	40	1.00	0.89	59.96	20.00	4.00	10.00	14.00	7482966.00	2089.00	5678.00
Lm3	GCF_001594305.1	SRR5515964	Ec	40	0.00	0.95	59.97	13.00	5.00	7.00	12.00	7465301.00	2625.00	4175.00
Lm4	GCF_000195435.3	SRR3715600	Ec	40	3.00	0.89	60.00	16.00	2.00	12.00	14.00	7296704.00	2390.00	4643.00
Lm5	GCF_000438585.1	SRR5617247	Ec	40	1.00	0.95	60.00	16.00	5.00	16.00	21.00	7371049.00	2113.00	5364.00
Lm6	GCF_000438705.2	SRR7085315	Ec	40	0.00	1.00	60.00	16.00	4.00	3.00	7.00	7220420.00	2098.00	5215.00
Lm7	GCF_000438605.1	SRR3040406	Ec	40	0.00	1.00	60.00	17.00	6.00	12.00	18.00	7417629.00	2441.00	4573.00
Lm8	GCF_001548635.1	SRR3050978	Ec	40	1.00	0.66	60.00	20.00	4.00	13.00	17.00	7535222.00	2428.00	4855.00
Median			Ec	40	1.00	0.93	60.00	16.00	4.50	12.00	15.50	7407421.00	2251.50	5035.00
Minimum			Ec	40	0.00	0.66	59.96	10.00			7.00	7220420.00	1949.00	4175.00
Maximum			Ec	40	5.00	1.00	60.00	20.00			23.00	7535222.00	2625.00	6357.00
Lm1	GCF_001952775.1	SRR6186591	Ec	50	5.00	0.92	50.00	40.00	12.00	40.00	52.00	7540500.00	1351.00	9154.00
Lm2	GCF_001548585.1	SRR2177969	Ec	50	1.00	0.84	49.96	44.00	15.00	23.00	38.00	7668789.00	1484.00	8650.00
Lm3	GCF_001594305.1	SRR5515964	Ec	50	0.00	0.93	49.98	40.00	23.00	24.00	47.00	7726954.00	1870.00	6339.00
Lm4	GCF_000195435.3	SRR3715600	Ec	50	3.00	0.84	50.00	46.00	9.00	27.00	36.00	7530365.00	1687.00	6855.00
Lm5	GCF_000438585.1	SRR5617247	Ec	50	1.00	0.93	50.00	36.00	15.00	24.00	39.00	7540700.00	1440.00	8473.00
Lm6	GCF_000438705.2	SRR7085315	Ec	50	0.00	1.00	50.00	38.00	16.00	23.00	39.00	7407869.00	1485.00	8411.00
Lm7	GCF_000438605.1	SRR3040406	Ec	50	0.00	1.00	50.00	36.00	12.00	34.00	46.00	7650467.00	1776.00	6783.00
Lm8	GCF_001548635.1	SRR3050978	Ec	50	1.00	0.60	50.00	25.00	12.00	35.00	47.00	7756608.00	1804.00	7080.00
Median			Ec	50	1.00	0.93	50.00	39.00	13.50	25.50	42.50	7595583.50	1586.00	7745.50
Minimum			Ec	50	0.00	0.60	49.96	25.00			36.00	7407869.00	1351.00	6339.00
Maximum			Ec	50	5.00	1.00	50.00	46.00			52.00	7756608.00	1870.00	9154.00

Note: Lm=*Listeria monocytogenes* , Se=*Salmonella enterica*, Ec=*Escherichia coli*

Table S2: Results of SNP pipeline and core-genome multi locus sequence typing analyses. (continued)

	Subject	Contaminant	Contaminant Type	Percent Contamination	SNPs	Bootstrap Support	Percent Reads Mapped	Alleles	Missing Alleles	Partial Alleles	Missing and Partial Alleles	Length	Number Contigs	N50
Se1	GCF_000623095.1	GCF_000623095.1	Self	0	2.00	1.00	100.00	6.00	1.00	0.00	1.00	4639863.00	33.00	386933.00
Se2	GCF_000272715.4	GCF_000272715.4	Self	0	1.00	1.00	99.99	5.00	1.00	0.00	1.00	4910912.00	53.00	330477.00
Se3	GCF_000341425.1	GCF_000341425.1	Self	0	0.00	1.00	100.00	0.00	1.00	2.00	3.00	4628884.00	32.00	539097.00
Se4	GCF_001441225.1	GCF_001441225.1	Self	0	3.00	1.00	100.00	0.00	0.00	0.00	0.00	4942434.00	49.00	248639.00
Se5	GCF_001535315.2	GCF_001535315.2	Self	0	2.00	0.56	100.00	1.00	0.00	2.00	2.00	4895110.00	48.00	241646.00
Se6	GCF_000430145.2	GCF_000430145.2	Self	0	0.00	1.00	100.00	0.00	0.00	0.00	0.00	5001878.00	65.00	255528.00
Se7	GCF_000430085.2	GCF_000430085.2	Self	0	6.00	1.00	100.00	3.00	0.00	0.00	0.00	4886122.00	36.00	339242.00
Se8	GCF_000487935.2	GCF_000487935.2	Self	0	0.00	1.00	100.00	1.00	0.00	3.00	3.00	5052014.00	40.00	406735.00
Median			Self	0	1.50	1.00	100.00	1.00	0.00	0.00	1.00	4903011.00	44.00	334859.50
Minimum			Self	0	0.00	0.56	99.99	0.00			0.00	4628884.00	32.00	241646.00
Maximum			Self	0	6.00	1.00	100.00	6.00			3.00	5052014.00	65.00	539097.00
Se1	GCF_000623095.1	GCF_000623095.1	Self	10	2.00	1.00	100.00	6.00	1.00	0.00	1.00	4638738.00	41.00	250733.00
Se2	GCF_000272715.4	GCF_000272715.4	Self	10	1.00	1.00	100.00	5.00	1.00	0.00	1.00	4907012.00	62.00	289457.00
Se3	GCF_000341425.1	GCF_000341425.1	Self	10	0.00	1.00	100.00	1.00	1.00	2.00	3.00	4628306.00	35.00	399755.00
Se4	GCF_001441225.1	GCF_001441225.1	Self	10	3.00	1.00	100.00	0.00	0.00	1.00	1.00	4941652.00	55.00	235221.00
Se5	GCF_001535315.2	GCF_001535315.2	Self	10	2.00	0.65	100.00	2.00	0.00	2.00	2.00	4894227.00	50.00	230779.00
Se6	GCF_000430145.2	GCF_000430145.2	Self	10	0.00	1.00	100.00	1.00	0.00	0.00	0.00	5001625.00	70.00	229559.00
Se7	GCF_000430085.2	GCF_000430085.2	Self	10	6.00	1.00	100.00	5.00	1.00	0.00	1.00	4884900.00	45.00	213713.00
Se8	GCF_000487935.2	GCF_000487935.2	Self	10	0.00	1.00	100.00	1.00	0.00	3.00	3.00	5050637.00	48.00	282786.00
Median			Self	10	1.50	1.00	100.00	1.50	0.50	0.50	1.00	4900619.50	49.00	242977.00
Minimum			Self	10	0.00	0.65	100.00	0.00			0.00	4628306.00	35.00	213713.00
Maximum			Self	10	6.00	1.00	100.00	6.00			3.00	5050637.00	70.00	399755.00
Se1	GCF_000623095.1	GCF_000623095.1	Self	20	2.00	1.00	100.00	7.00	1.00	1.00	2.00	4639771.00	43.00	345397.00
Se2	GCF_000272715.4	GCF_000272715.4	Self	20	1.00	1.00	100.00	5.00	1.00	0.00	1.00	4906713.00	66.00	205452.00
Se3	GCF_000341425.1	GCF_000341425.1	Self	20	0.00	1.00	100.00	0.00	1.00	3.00	4.00	4629174.00	41.00	356165.00
Se4	GCF_001441225.1	GCF_001441225.1	Self	20	3.00	1.00	100.00	1.00	0.00	1.00	1.00	4941933.00	58.00	201714.00
Se5	GCF_001535315.2	GCF_001535315.2	Self	20	2.00	0.64	100.00	2.00	0.00	2.00	2.00	4894104.00	57.00	230779.00
Se6	GCF_000430145.2	GCF_000430145.2	Self	20	0.00	1.00	100.00	2.00	1.00	1.00	2.00	5002104.00	76.00	207982.00
Se7	GCF_000430085.2	GCF_000430085.2	Self	20	6.00	1.00	100.00	3.00	0.00	0.00	0.00	4884544.00	51.00	192851.00
Se8	GCF_000487935.2	GCF_000487935.2	Self	20	0.00	1.00	100.00	2.00	1.00	3.00	4.00	5049839.00	53.00	230762.00
Median			Self	20	1.50	1.00	100.00	2.00	1.00	1.00	2.00	4900408.50	55.00	219372.00
Minimum			Self	20	0.00	0.64	100.00	0.00			0.00	4629174.00	41.00	192851.00
Maximum			Self	20	6.00	1.00	100.00	7.00			4.00	5049839.00	76.00	356165.00
Se1	GCF_000623095.1	GCF_000623095.1	Self	30	2.00	1.00	100.00	7.00	1.00	0.00	1.00	4638774.00	52.00	160581.00
Se2	GCF_000272715.4	GCF_000272715.4	Self	30	1.00	0.99	100.00	8.00	1.00	0.00	1.00	4907244.00	66.00	219609.00
Se3	GCF_000341425.1	GCF_000341425.1	Self	30	0.00	1.00	100.00	0.00	1.00	6.00	7.00	4627693.00	50.00	205884.00
Se4	GCF_001441225.1	GCF_001441225.1	Self	30	3.00	1.00	100.00	4.00	1.00	0.00	1.00	4939968.00	71.00	201714.00
Se5	GCF_001535315.2	GCF_001535315.2	Self	30	2.00	0.64	100.00	3.00	1.00	2.00	3.00	4895336.00	64.00	153972.00
Se6	GCF_000430145.2	GCF_000430145.2	Self	30	0.00	1.00	100.00	0.00	1.00	1.00	2.00	5000209.00	85.00	143119.00
Se7	GCF_000430085.2	GCF_000430085.2	Self	30	6.00	1.00	100.00	7.00	0.00	0.00	0.00	4885387.00	53.00	172013.00
Se8	GCF_000487935.2	GCF_000487935.2	Self	30	0.00	1.00	100.00	5.00	1.00	4.00	5.00	5050440.00	62.00	194095.00
Median			Self	30	1.50	1.00	100.00	4.50	1.00	0.50	1.50	4901290.00	63.00	183054.00
Minimum			Self	30	0.00	0.64	100.00	0.00			0.00	4627693.00	50.00	143119.00
Maximum			Self	30	6.00	1.00	100.00	8.00			7.00	5050440.00	85.00	219609.00
Se1	GCF_000623095.1	GCF_000623095.1	Self	40	2.00	1.00	100.00	7.00	1.00	2.00	3.00	4637092.00	57.00	139183.00
Se2	GCF_000272715.4	GCF_000272715.4	Self	40	1.00	1.00	100.00	6.00	2.00	0.00	2.00	4906488.00	77.00	171169.00
Se3	GCF_000341425.1	GCF_000341425.1	Self	40	0.00	1.00	100.00	6.00	2.00	4.00	6.00	4626608.00	51.00	191319.00
Se4	GCF_001441225.1	GCF_001441225.1	Self	40	3.00	1.00	100.00	5.00	0.00	1.00	1.00	4941382.00	71.00	167662.00
Se5	GCF_001535315.2	GCF_001535315.2	Self	40	2.00	0.58	100.00	1.00	3.00	2.00	5.00	4894944.00	73.00	117307.00
Se6	GCF_000430145.2	GCF_000430145.2	Self	40	0.00	1.00	100.00	2.00	0.00	3.00	3.00	4999772.00	84.00	206820.00
Se7	GCF_000430085.2	GCF_000430085.2	Self	40	6.00	1.00	100.00	5.00	0.00	1.00	1.00	4885930.00	50.00	200299.00
Se8	GCF_000487935.2	GCF_000487935.2	Self	40	0.00	1.00	100.00	3.00	0.00	4.00	4.00	5049051.00	72.00	149316.00
Median			Self	40	1.50	1.00	100.00	5.00	0.50	2.00	3.00	4900716.00	71.50	169415.50
Minimum			Self	40	0.00	0.58	100.00	1.00			1.00	4626608.00	50.00	117307.00
Maximum			Self	40	6.00	1.00	100.00	7.00			6.00	5049051.00	84.00	206820.00
Se1	GCF_000623095.1	GCF_000623095.1	Self	50	2.00	1.00	100.00	12.00	2.00	0.00	2.00	4639269.00	56.00	160115.00
Se2	GCF_000272715.4	GCF_000272715.4	Self	50	1.00	1.00	100.00	6.00	1.00	2.00	3.00	4909884.00	74.00	289586.00
Se3	GCF_000341425.1	GCF_000341425.1	Self	50	0.00	1.00	100.00	5.00	1.00	3.00	4.00	4627278.00	56.00	176901.00
Se4	GCF_001441225.1	GCF_001441225.1	Self	50	3.00	1.00	100.00	4.00	0.00	3.00	3.00	4938382.00	66.00	172013.00
Se5	GCF_001535315.2	GCF_001535315.2	Self	50	2.00	0.63	100.00	7.00	1.00	7.00	8.00	4894609.00	80.00	130253.00
Se6	GCF_000430145.2	GCF_000430145.2	Self	50	0.00	1.00	100.00	2.00	2.00	0.00	2.00	5000403.00	89.00	114934.00
Se7	GCF_000430085.2	GCF_000430085.2	Self	50	6.00	1.00	100.00	8.00	2.00	3.00	5.00	4882886.00	68.00	138317.00
Se8	GCF_000487935.2	GCF_000487935.2	Self	50	0.00	1.00	100.00	5.00	0.00	5.00	5.00	5050300.00	75.00	149375.00
Median			Self	50	1.50	1.00	100.00	5.50	1.00	3.00	3.50	4902246.50	71.00	154745.00
Minimum			Self	50	0.00	0.63	100.00	2.00			2.00	4627278.00	56.00	114934.00
Maximum			Self	50	6.00	1.00	100.00	12.00			8.00	5050300.00	89.00	289586.00
Se1	GCF_000623095.1	SRR3667831	0.05	10	2.00	1.00	99.81	6.00	1.00	3.00	4.00	4680288.00	86.00	227886.00
Se2	GCF_000272715.4	SRR5714198	0.05	10	1.00	1.00	99.30	5.00	0.00	0.00	0.00	5056886.00	229.00	377654.00
Se3	GCF_000341425.1	SRR5291615	0.05	10	0.00	1.00	99.81	0.00	2.00	1.00	3.00	4664612.00	83.00	332915.00
Se4	GCF_001441225.1	SRR718796	0.05	10	3.00	1.00	99.71	0.00	0.00	0.00	0.00	5003678.00	120.00	234965.00
Se5	GCF_001535315.2	SRR3229336	0.05	10	2.00	0.59	99.75	1.00	0.00	1.00	1.00	4943202.00	106.00	199391.00
Se6	GCF_000430145.2	SRR6243450	0.05	10	0.00	1.00	99.70	0.00	0.00	0.00	0.00	5056547.00	128.00	216487.00
Se7	GCF_000430085.2	SRR2039126	0.05	10	6.00	1.00	99.61	4.00	0.00	0.00	0.00	4974916.00	151.00	234825.00
Se8	GCF_000487935.2	SRR2979535	0.05	10	0.00	1.00	99.57	0.00	0.00	4.00	4.00	5139305.00	158.00	283190.00
Median			0.05	10	1.50	1.00	99.71	0.50	0.00	0.50	0.50	4989297.00	124.00	234895.00
Minimum			0.05	10	0.00	0.59	99.30	0.00			0.00	4664612.00	83.00	199391.00
Maximum			0.05	10	6.00	1.00	99.81	6.00			4.00	5139305.00	229.00	377654.00
Se1	GCF_000623095.1	SRR3667831	0.05	20	2.00	1.00	99.61	9.00	4.00	9.00	13.00	4717025.00	172.00	67080.00
Se2	GCF_000272715.4	SRR5714198	0.05	20	1.00	1.00	98.62	6.00	0.00	1.00	1.00	5183324.00	257.00	224713.00
Se3	GCF_000341425.1	SRR5291615	0.05	20	0.00	1.00	99.63	2.00	5.00	3.00	8.00	4696213.00	164.00	79697.00
Se4	GCF_001441225.1	SRR718796	0.05	20	3.00	1.00	99.48	0.00	2.00	5.00	7.00	5041567.00	184.00	138427.00
Se5	GCF_001535315.2	SRR3229336	0.05	20	2.00	0.61	99.52	2.00	1.00	1.00	2.00	4983614.00	134.00	132333.00
Se6	GCF_000430145.2	SRR6243450	0.05	20	0.00	1.00	99.41	3.00	1.00	2.00	3.00	5101247.00	168.00	122635.00
Se7	GCF_000430085.2	SRR2039126	0.05	20	6.00	1.00	99.25	4.00	0.00	0.00	0.00	5040542.00	171.00	298521.00
Se8	GCF_000487935.2	SRR2979535	0.05	20	1.00	1.00	99.13	3.00	2.00	7.00	9.00	5226504.00	217.00	166128.00
Median			0.05	20	1.50	1.00	99.45	3.00	1.50	2.50	5.00	5041054.50	171.50	135380.00
Minimum			0.05	20	0.00	0.61	98.62	0.00			0.00	4696213.00	134.00	67080.00
Maximum			0.05	20	6.00	1.00	99.63	9.00			13.00	5226504.00	257.00	298521.00
Se1	GCF_000623095.1	SRR3667831	0.05	30	3.00	1.00	99.45	21.00	10.00	18.00	28.00	4731038.00	336.00	26524.00
Se2	GCF_000272715.4	SRR5714198	0.05	30	1.00	1.00	97.88	9.00	3.00	1.00	4.00	5238615.00	224.00	96527.00
Se3	GCF_000341425.1	SRR5291615	0.05	30	2.00	1.00	99.44	11.00	8.00	17.00	25.00	4716886.00	305.00	35187.00
Se4	GCF_001441225.1	SRR718796	0.05	30	3.00	1.00	99.18	8.00	3.00	10.00	13.00	5057795.00	256.00	89094.00
Se5	GCF_001535315.2	SRR3229336	0.05	30	2.00	0.00	99.26	3.00	1.00	3.00	4.00	5002542.00	168.00	90289.00
Se6	GCF_000430145.2	SRR6243450	0.05	30	1.00	1.00	99.15	5.00	5.00	8.00	13.00	5119155.00	270.00	48379.00
Se7	GCF_000430085.2	SRR2039126	0.05	30	7.00	1.00	98.91	5.00	0.00	1.00	1.00	5060758.00	181.00	114404.00
Se8	GCF_000487935.2	SRR2979535	0.05	30	1.00	1.00	98.73	6.00	2.00	16.00	18.00	5236220.00	263.00	76096.00
Median			0.05	30	2.00	1.00	99.17	7.00	3.00	9.00	13.00	5059276.50	259.50	82595.00
Minimum			0.05	30	1.00	0.00	97.88	3.00			1.00	4716886.00	168.00	26524.00
Maximum			0.05	30	7.00	1.00	99.45	21.00			28.00	5238615.00	336.00	114404.00
Se1	GCF_000623095.1	SRR3667831	0.05	40	5.00	1.00	99.21	47.00	16.00	42.00	58.00	4729900.00	615.00	12768.00
Se2	GCF_000272715.4	SRR5714198	0.05	40	0.00	0.98	97.22	10.00	3.00	8.00	11.00	5255250.00	274.00	65807.00
Se3	GCF_000341425.1	SRR5291615	0.05	40	5.00	0.92	99.24	31.00	20.00	29.00	49.00	4712331.00	462.00	16985.00
Se4	GCF_001441225.1	SRR718796	0.05	40	10.00	1.00	98.93	19.00	6.00	19.00	25.00	5052887.00	316.00	55320.00
Se5	GCF_001535315.2	SRR3229336	0.05	40	1.00	0.00	99.02	5.00	4.00	5.00	9.00	5014005.00	238.00	47265.00
Se6	GCF_000430145.2	SRR6243450	0.05	40	1.00	1.00	98.86	15.00	6.00	11.00	17.00	5132254.00	353.00	34217.00
Se7	GCF_000430085.2	SRR2039126	0.05	40	8.00	1.00	98.50	5.00	0.00	0.00	0.00	5070538.00	194.00	120168.00
Se8	GCF_000487935.2	SRR2979535	0.05	40	1.00	1.00	98.33	19.00	12.00	18.00	30.00	5229487.00	403.00	38004.00
Median			0.05	40	3.00	1.00	98.90	17.00	6.00	14.50	21.00	5061712.50	334.50	42634.50
Minimum			0.05	40	0.00	0.00	97.22	5.00			0.00	4712331.00	194.00	12768.00
Maximum			0.05	40	10.00	1.00	99.24	47.00			58.00	5255250.00	615.00	120168.00
Se1	GCF_000623095.1	SRR3667831	0.05	50	18.00	0.83	99.03	76.00	26.00	60.00	86.00	4719016.00	727.00	11041.00
Se2	GCF_000272715.4	SRR5714198	0.05	50	4.00	0.84	96.47	18.00	5.00	5.00	10.00	5267226.00	244.00	58684.00
Se3	GCF_000341425.1	SRR5291615	0.05	50	10.00	0.87	99.05	48.00	19.00	28.00	47.00	4714966.00	629.00	12403.00
Se4	GCF_001441225.1	SRR718796	0.05	50	19.00	0.47	98.63	28.00	9.00	24.00	33.00	5054071.00	361.00	49296.00
Se5	GCF_001535315.2	SRR3229336	0.05	50	3.00	0.00	98.73	14.00	1.00	6.00	7.00	5020075.00	270.00	45008.00
Se6	GCF_000430145.2	SRR6243450	0.05	50	9.00	0.99	98.54	29.00	7.00	8.00	15.00	5134301.00	400.00	26152.00
Se7	GCF_000430085.2	SRR2039126	0.05	50	17.00	0.92	98.17	5.00	0.00	0.00	0.00	5061275.00	187.00	127369.00
Se8	GCF_000487935.2	SRR2979535	0.05	50	18.00	0.99	97.86	21.00	10.00	17.00	27.00	5230731.00	414.00	35914.00
Median			0.05	50	13.50	0.86	98.59	24.50	8.00	12.50	21.00	5057673.00	380.50	40461.00
Minimum			0.05	50	3.00	0.00	96.47	5.00			0.00	4714966.00	187.00	11041.00
Maximum			0.05	50	19.00	0.99	99.05	76.00			86.00	5267226.00	727.00	127369.00
Se1	GCF_000623095.1	SRR6132093	0.5	10	2.00	1.00	99.79	12.00	1.00	5.00	6.00	4678178.00	109.00	137447.00
Se2	GCF_000272715.4	SRR1952754	0.5	10	1.00	1.00	99.32	6.00	0.00	0.00	0.00	5053848.00	246.00	301626.00
Se3	GCF_000341425.1	SRR6514374	0.5	10	0.00	1.00	99.27	0.00	1.00	2.00	3.00	4767772.00	230.00	180369.00
Se4	GCF_001441225.1	SRR7284342	0.5	10	3.00	1.00	99.73	2.00	1.00	1.00	2.00	4993166.00	143.00	157228.00
Se5	GCF_001535315.2	SRR1576008	0.5	10	2.00	0.60	99.09	7.00	1.00	1.00	2.00	5092758.00	300.00	153972.00
Se6	GCF_000430145.2	SRR5945527	0.5	10	0.00	1.00	99.62	3.00	1.00	2.00	3.00	5084064.00	192.00	105666.00
Se7	GCF_000430085.2	SRR1645762	0.5	10	6.00	1.00	99.51	10.00	1.00	2.00	3.00	4986081.00	169.00	161949.00
Se8	GCF_000487935.2	SRR6765816	0.5	10	0.00	1.00	99.50	5.00	0.00	4.00	4.00	5145627.00	168.00	230878.00
Median			0.5	10	1.50	1.00	99.51	5.50	1.00	2.00	3.00	5023507.00	180.50	159588.50
Minimum			0.5	10	0.00	0.60	99.09	0.00			0.00	4678178.00	109.00	105666.00
Maximum			0.5	10	6.00	1.00	99.79	12.00			6.00	5145627.00	300.00	301626.00
Se1	GCF_000623095.1	SRR6132093	0.5	20	2.00	1.00	99.60	36.00	16.00	38.00	54.00	4692530.00	450.00	22473.00
Se2	GCF_000272715.4	SRR1952754	0.5	20	0.00	1.00	98.57	28.00	17.00	27.00	44.00	5184058.00	596.00	25282.00
Se3	GCF_000341425.1	SRR6514374	0.5	20	0.00	1.00	98.55	24.00	13.00	35.00	48.00	4885817.00	544.00	22987.00
Se4	GCF_001441225.1	SRR7284342	0.5	20	3.00	1.00	99.47	31.00	11.00	27.00	38.00	5019808.00	501.00	29802.00
Se5	GCF_001535315.2	SRR1576008	0.5	20	2.00	0.69	98.16	30.00	11.00	25.00	36.00	5246507.00	617.00	27272.00
Se6	GCF_000430145.2	SRR5945527	0.5	20	0.00	1.00	99.24	28.00	11.00	33.00	44.00	5127220.00	570.00	20596.00
Se7	GCF_000430085.2	SRR1645762	0.5	20	6.00	1.00	99.00	26.00	13.00	18.00	31.00	5060078.00	498.00	31269.00
Se8	GCF_000487935.2	SRR6765816	0.5	20	0.00	1.00	99.01	32.00	18.00	42.00	60.00	5213854.00	633.00	18084.00
Median			0.5	20	1.00	1.00	99.01	29.00	13.00	30.00	44.00	5093649.00	557.00	24134.50
Minimum			0.5	20	0.00	0.69	98.16	24.00			31.00	4692530.00	450.00	18084.00
Maximum			0.5	20	6.00	1.00	99.60	36.00			60.00	5246507.00	633.00	31269.00
Se1	GCF_000623095.1	SRR6132093	0.5	30	3.00	1.00	99.43	129.00	42.00	91.00	133.00	4622144.00	1121.00	8147.00
Se2	GCF_000272715.4	SRR1952754	0.5	30	0.00	1.00	97.79	66.00	48.00	98.00	146.00	5118750.00	1415.00	7692.00
Se3	GCF_000341425.1	SRR6514374	0.5	30	1.00	1.00	97.79	83.00	45.00	113.00	158.00	4817379.00	1522.00	5253.00
Se4	GCF_001441225.1	SRR7284342	0.5	30	4.00	1.00	99.22	91.00	42.00	81.00	123.00	4957228.00	1151.00	11042.00
Se5	GCF_001535315.2	SRR1576008	0.5	30	3.00	0.59	97.30	87.00	49.00	119.00	168.00	5187514.00	1534.00	6535.00
Se6	GCF_000430145.2	SRR5945527	0.5	30	0.00	1.00	98.83	77.00	56.00	141.00	197.00	5007337.00	1656.00	5573.00
Se7	GCF_000430085.2	SRR1645762	0.5	30	8.00	1.00	98.49	70.00	52.00	112.00	164.00	4954559.00	1441.00	9593.00
Se8	GCF_000487935.2	SRR6765816	0.5	30	2.00	1.00	98.52	93.00	57.00	152.00	209.00	5065645.00	1781.00	5457.00
Median			0.5	30	2.50	1.00	98.51	85.00	48.50	112.50	161.00	4982282.50	1481.50	7113.50
Minimum			0.5	30	0.00	0.59	97.30	66.00			123.00	4622144.00	1121.00	5253.00
Maximum			0.5	30	8.00	1.00	99.43	129.00			209.00	5187514.00	1781.00	11042.00
Se1	GCF_000623095.1	SRR6132093	0.5	40	13.00	1.00	99.21	201.00	81.00	219.00	300.00	4412589.00	2133.00	3406.00
Se2	GCF_000272715.4	SRR1952754	0.5	40	3.00	1.00	97.10	148.00	52.00	168.00	220.00	4987150.00	1899.00	6225.00
Se3	GCF_000341425.1	SRR6514374	0.5	40	8.00	1.00	96.99	156.00	75.00	198.00	273.00	4689688.00	2048.00	4047.00
Se4	GCF_001441225.1	SRR7284342	0.5	40	22.00	1.00	98.94	173.00	64.00	203.00	267.00	4741414.00	1984.00	6301.00
Se5	GCF_001535315.2	SRR1576008	0.5	40	13.00	0.54	96.48	161.00	68.00	188.00	256.00	5043007.00	2008.00	6333.00
Se6	GCF_000430145.2	SRR5945527	0.5	40	5.00	1.00	98.48	185.00	66.00	220.00	286.00	4849332.00	2252.00	4168.00
Se7	GCF_000430085.2	SRR1645762	0.5	40	22.00	1.00	97.99	137.00	83.00	172.00	255.00	4843316.00	1903.00	6958.00
Se8	GCF_000487935.2	SRR6765816	0.5	40	9.00	1.00	98.04	179.00	67.00	226.00	293.00	4942756.00	2249.00	4258.00
Median			0.5	40	11.00	1.00	98.02	167.00	67.50	200.50	270.00	4846324.00	2028.00	5241.50
Minimum			0.5	40	3.00	0.54	96.48	137.00			220.00	4412589.00	1899.00	3406.00
Maximum			0.5	40	22.00	1.00	99.21	201.00			300.00	5043007.00	2252.00	6958.00
Se1	GCF_000623095.1	SRR6132093	0.5	50	68.00	0.95	99.01	311.00	107.00	221.00	328.00	4348792.00	2291.00	3157.00
Se2	GCF_000272715.4	SRR1952754	0.5	50	37.00	0.97	96.36	232.00	75.00	175.00	250.00	4944949.00	1994.00	5759.00
Se3	GCF_000341425.1	SRR6514374	0.5	50	38.00	1.00	96.30	288.00	83.00	217.00	300.00	4640358.00	2266.00	3166.00
Se4	GCF_001441225.1	SRR7284342	0.5	50	71.00	0.00	98.69	293.00	58.00	216.00	274.00	4728449.00	2086.00	6208.00
Se5	GCF_001535315.2	SRR1576008	0.5	50	44.00	0.65	95.52	245.00	105.00	195.00	300.00	4991699.00	2172.00	6008.00
Se6	GCF_000430145.2	SRR5945527	0.5	50	67.00	0.98	98.08	275.00	81.00	234.00	315.00	4785224.00	2358.00	3908.00
Se7	GCF_000430085.2	SRR1645762	0.5	50	46.00	0.91	97.48	242.00	75.00	185.00	260.00	4795046.00	2091.00	7052.00
Se8	GCF_000487935.2	SRR6765816	0.5	50	82.00	0.99	97.53	283.00	72.00	241.00	313.00	4883879.00	2396.00	3744.00
Median			0.5	50	56.50	0.96	97.51	279.00	78.00	216.50	300.00	4790135.00	2219.00	4833.50
Minimum			0.5	50	37.00	0.00	95.52	232.00			250.00	4348792.00	1994.00	3157.00
Maximum			0.5	50	82.00	1.00	99.01	311.00			328.00	4991699.00	2396.00	7052.00
Se1	GCF_000623095.1	SRR7125364	5	10	2.00	1.00	96.62	19.00	1.00	1.00	2.00	5161488.00	694.00	195754.00
Se2	GCF_000272715.4	SRR4202526	5	10	1.00	1.00	96.84	24.00	1.00	2.00	3.00	5429582.00	709.00	149188.00
Se3	GCF_000341425.1	SRR6129500	5	10	0.00	1.00	96.61	5.00	3.00	1.00	4.00	5159218.00	696.00	188792.00
Se4	GCF_001441225.1	SRR4202526	5	10	3.00	1.00	96.77	19.00	2.00	1.00	3.00	5485153.00	739.00	138803.00
Se5	GCF_001535315.2	SRR5581942	5	10	2.00	0.71	96.53	16.00	1.00	6.00	7.00	5500484.00	816.00	131737.00
Se6	GCF_000430145.2	SRR6452759	5	10	0.00	1.00	96.76	17.00	0.00	0.00	0.00	5566985.00	778.00	115138.00
Se7	GCF_000430085.2	SRR4202526	5	10	6.00	1.00	96.86	23.00	1.00	0.00	1.00	5398571.00	696.00	160459.00
Se8	GCF_000487935.2	SRR5581942	5	10	0.00	1.00	96.47	6.00	0.00	6.00	6.00	5651485.00	797.00	138648.00
Median			5	10	1.50	1.00	96.69	18.00	1.00	1.00	3.00	5457367.50	724.00	143995.50
Minimum			5	10	0.00	0.71	96.47	5.00			0.00	5159218.00	694.00	115138.00
Maximum			5	10	6.00	1.00	96.86	24.00			7.00	5651485.00	816.00	195754.00
Se1	GCF_000623095.1	SRR7125364	5	20	2.00	1.00	93.23	130.00	10.00	15.00	25.00	5789693.00	1237.00	24646.00
Se2	GCF_000272715.4	SRR4202526	5	20	1.00	1.00	93.63	167.00	14.00	17.00	31.00	6044629.00	1267.00	19915.00
Se3	GCF_000341425.1	SRR6129500	5	20	0.00	1.00	93.21	113.00	9.00	16.00	25.00	5793194.00	1234.00	26971.00
Se4	GCF_001441225.1	SRR4202526	5	20	4.00	1.00	93.44	143.00	10.00	30.00	40.00	6121994.00	1279.00	19308.00
Se5	GCF_001535315.2	SRR5581942	5	20	2.00	0.66	93.10	140.00	4.00	19.00	23.00	6160666.00	1331.00	21005.00
Se6	GCF_000430145.2	SRR6452759	5	20	2.00	1.00	93.50	148.00	8.00	39.00	47.00	6197083.00	1341.00	17854.00
Se7	GCF_000430085.2	SRR4202526	5	20	6.00	1.00	93.60	128.00	13.00	28.00	41.00	6067481.00	1330.00	18955.00
Se8	GCF_000487935.2	SRR5581942	5	20	0.00	1.00	93.01	143.00	8.00	32.00	40.00	6356340.00	1371.00	20167.00
Median			5	20	2.00	1.00	93.34	141.50	9.50	23.50	35.50	6094737.50	1304.50	20041.00
Minimum			5	20	0.00	0.66	93.01	113.00			23.00	5789693.00	1234.00	17854.00
Maximum			5	20	6.00	1.00	93.63	167.00			47.00	6356340.00	1371.00	26971.00
Se1	GCF_000623095.1	SRR7125364	5	30	5.00	1.00	89.95	226.00	73.00	188.00	261.00	6579981.00	3252.00	2903.00
Se2	GCF_000272715.4	SRR4202526	5	30	3.00	1.00	90.46	180.00	84.00	288.00	372.00	7117562.00	4187.00	2337.00
Se3	GCF_000341425.1	SRR6129500	5	30	1.00	1.00	89.88	235.00	82.00	174.00	256.00	6561000.00	3264.00	2954.00
Se4	GCF_001441225.1	SRR4202526	5	30	9.00	1.00	90.32	153.00	75.00	280.00	355.00	7207948.00	4132.00	2324.00
Se5	GCF_001535315.2	SRR5581942	5	30	2.00	0.59	89.60	156.00	83.00	266.00	349.00	7154351.00	4038.00	2363.00
Se6	GCF_000430145.2	SRR6452759	5	30	1.00	1.00	90.23	160.00	69.00	276.00	345.00	7328627.00	4296.00	2290.00
Se7	GCF_000430085.2	SRR4202526	5	30	6.00	1.00	90.41	151.00	85.00	251.00	336.00	7115714.00	4088.00	2325.00
Se8	GCF_000487935.2	SRR5581942	5	30	5.00	1.00	89.50	139.00	73.00	262.00	335.00	7435542.00	4094.00	2569.00
Median			5	30	4.00	1.00	90.09	158.00	78.50	264.00	340.50	7135956.50	4091.00	2350.00
Minimum			5	30	1.00	0.59	89.50	139.00			256.00	6561000.00	3252.00	2290.00
Maximum			5	30	9.00	1.00	90.46	235.00			372.00	7435542.00	4296.00	2954.00
Se1	GCF_000623095.1	SRR7125364	5	40	19.00	1.00	86.54	259.00	90.00	261.00	351.00	7548568.00	4418.00	2378.00
Se2	GCF_000272715.4	SRR4202526	5	40	43.00	1.00	87.25	258.00	61.00	264.00	325.00	7890838.00	4191.00	2669.00
Se3	GCF_000341425.1	SRR6129500	5	40	16.00	1.00	86.47	241.00	76.00	288.00	364.00	7553927.00	4467.00	2281.00
Se4	GCF_001441225.1	SRR4202526	5	40	39.00	1.00	87.17	279.00	99.00	257.00	356.00	7951322.00	4169.00	2656.00
Se5	GCF_001535315.2	SRR5581942	5	40	29.00	0.62	86.16	242.00	103.00	279.00	382.00	7932819.00	4314.00	2637.00
Se6	GCF_000430145.2	SRR6452759	5	40	27.00	1.00	87.21	249.00	83.00	231.00	314.00	8018027.00	4295.00	2690.00
Se7	GCF_000430085.2	SRR4202526	5	40	41.00	1.00	87.33	251.00	79.00	239.00	318.00	7915617.00	4233.00	2643.00
Se8	GCF_000487935.2	SRR5581942	5	40	30.00	1.00	86.03	256.00	83.00	277.00	360.00	8180942.00	4331.00	2758.00
Median			5	40	29.50	1.00	86.86	253.50	83.00	262.50	353.50	7924218.00	4304.50	2649.50
Minimum			5	40	16.00	0.62	86.03	241.00			314.00	7548568.00	4169.00	2281.00
Maximum			5	40	43.00	1.00	87.33	279.00			382.00	8180942.00	4467.00	2758.00
Se1	GCF_000623095.1	SRR7125364	5	50	103.00	0.99	83.13	398.00	76.00	246.00	322.00	7820207.00	4173.00	2636.00
Se2	GCF_000272715.4	SRR4202526	5	50	163.00	0.99	84.07	409.00	101.00	247.00	348.00	8047014.00	4030.00	2883.00
Se3	GCF_000341425.1	SRR6129500	5	50	106.00	1.00	83.12	414.00	77.00	236.00	313.00	7828333.00	4253.00	2537.00
Se4	GCF_001441225.1	SRR4202526	5	50	199.00	0.95	83.95	406.00	91.00	216.00	307.00	8129095.00	3991.00	2957.00
Se5	GCF_001535315.2	SRR5581942	5	50	123.00	0.61	82.70	389.00	101.00	236.00	337.00	8152229.00	4040.00	2983.00
Se6	GCF_000430145.2	SRR6452759	5	50	191.00	1.00	83.93	423.00	91.00	234.00	325.00	8167793.00	4025.00	2986.00
Se7	GCF_000430085.2	SRR4202526	5	50	147.00	1.00	84.10	436.00	86.00	233.00	319.00	8064007.00	3991.00	3010.00
Se8	GCF_000487935.2	SRR5581942	5	50	161.00	1.00	82.69	410.00	98.00	246.00	344.00	8370596.00	4165.00	2939.00
Median			5	50	154.00	1.00	83.53	409.50	91.00	236.00	323.50	8096551.00	4035.00	2948.00
Minimum			5	50	103.00	0.61	82.69	389.00			307.00	7820207.00	3991.00	2537.00
Maximum			5	50	199.00	1.00	84.10	436.00			348.00	8370596.00	4253.00	3010.00
Se1	GCF_000623095.1	SRR1520085	Lm	10	2.00	1.00	90.00	6.00	6.00	0.00	6.00	6804124.00	2022.00	134830.00
Se2	GCF_000272715.4	ERR1599704	Lm	10	1.00	1.00	90.00	5.00	6.00	0.00	6.00	7141623.00	1895.00	124644.00
Se3	GCF_000341425.1	SRR2585434	Lm	10	0.00	1.00	90.00	2.00	5.00	2.00	7.00	6774347.00	1924.00	151818.00
Se4	GCF_001441225.1	ERR1102444	Lm	10	3.00	1.00	90.00	0.00	4.00	0.00	4.00	7173170.00	1905.00	143743.00
Se5	GCF_001535315.2	SRR1166853	Lm	10	2.00	0.67	90.00	1.00	5.00	2.00	7.00	7114148.00	1901.00	122122.00
Se6	GCF_000430145.2	SRR2650300	Lm	10	0.00	1.00	90.00	0.00	6.00	0.00	6.00	7244910.00	1867.00	95697.00
Se7	GCF_000430085.2	SRR1207546	Lm	10	6.00	1.00	90.00	3.00	7.00	1.00	8.00	7105148.00	1984.00	140376.00
Se8	GCF_000487935.2	SRR3223762	Lm	10	0.00	1.00	90.00	1.00	5.00	3.00	8.00	7301706.00	1885.00	161895.00
Median			Lm	10	1.50	1.00	90.00	1.50	5.50	0.50	6.50	7127885.50	1903.00	137603.00
Minimum			Lm	10	0.00	0.67	90.00	0.00			4.00	6774347.00	1867.00	95697.00
Maximum			Lm	10	6.00	1.00	90.00	6.00			8.00	7301706.00	2022.00	161895.00
Se1	GCF_000623095.1	SRR1520085	Lm	20	2.00	1.00	80.00	7.00	7.00	1.00	8.00	7556038.00	1180.00	53313.00
Se2	GCF_000272715.4	ERR1599704	Lm	20	1.00	1.00	80.00	6.00	8.00	2.00	10.00	7689240.00	708.00	87089.00
Se3	GCF_000341425.1	SRR2585434	Lm	20	0.00	1.00	80.00	2.00	8.00	2.00	10.00	7400563.00	995.00	101636.00
Se4	GCF_001441225.1	ERR1102444	Lm	20	3.00	1.00	80.00	2.00	7.00	1.00	8.00	7756655.00	725.00	89094.00
Se5	GCF_001535315.2	SRR1166853	Lm	20	2.00	0.62	80.00	2.00	7.00	4.00	11.00	7719949.00	719.00	79053.00
Se6	GCF_000430145.2	SRR2650300	Lm	20	0.00	1.00	80.00	2.00	8.00	0.00	8.00	7779223.00	653.00	80570.00
Se7	GCF_000430085.2	SRR1207546	Lm	20	6.00	1.00	80.00	5.00	8.00	1.00	9.00	7791160.00	1064.00	84721.00
Se8	GCF_000487935.2	SRR3223762	Lm	20	0.00	1.00	80.00	4.00	7.00	3.00	10.00	7879598.00	669.00	111409.00
Median			Lm	20	1.50	1.00	80.00	3.00	7.50	1.50	9.50	7738302.00	722.00	85905.00
Minimum			Lm	20	0.00	0.62	80.00	2.00			8.00	7400563.00	653.00	53313.00
Maximum			Lm	20	6.00	1.00	80.00	7.00			11.00	7879598.00	1180.00	111409.00
Se1	GCF_000623095.1	SRR1520085	Lm	30	2.00	1.00	70.00	8.00	10.00	4.00	14.00	7612333.00	329.00	48683.00
Se2	GCF_000272715.4	ERR1599704	Lm	30	1.00	0.99	70.00	10.00	11.00	2.00	13.00	7730969.00	235.00	77039.00
Se3	GCF_000341425.1	SRR2585434	Lm	30	0.00	1.00	70.00	5.00	9.00	6.00	15.00	7441519.00	264.00	55336.00
Se4	GCF_001441225.1	ERR1102444	Lm	30	3.00	1.00	70.00	4.00	9.00	4.00	13.00	7803757.00	234.00	73320.00
Se5	GCF_001535315.2	SRR1166853	Lm	30	2.00	0.60	70.00	4.00	9.00	5.00	14.00	7761531.00	232.00	66265.00
Se6	GCF_000430145.2	SRR2650300	Lm	30	0.00	1.00	70.00	4.00	9.00	1.00	10.00	7819721.00	236.00	63221.00
Se7	GCF_000430085.2	SRR1207546	Lm	30	6.00	1.00	70.00	9.00	11.00	3.00	14.00	7879393.00	271.00	75900.00
Se8	GCF_000487935.2	SRR3223762	Lm	30	0.00	1.00	70.00	3.00	10.00	5.00	15.00	7923438.00	243.00	69653.00
Median			Lm	30	1.50	1.00	70.00	4.50	9.50	4.00	14.00	7782644.00	239.50	67959.00
Minimum			Lm	30	0.00	0.60	70.00	3.00			10.00	7441519.00	232.00	48683.00
Maximum			Lm	30	6.00	1.00	70.00	10.00			15.00	7923438.00	329.00	77039.00
Se1	GCF_000623095.1	SRR1520085	Lm	40	2.00	1.00	60.00	18.00	13.00	5.00	18.00	7618008.00	188.00	74348.00
Se2	GCF_000272715.4	ERR1599704	Lm	40	1.00	1.00	60.00	20.00	12.00	7.00	19.00	7724920.00	212.00	75649.00
Se3	GCF_000341425.1	SRR2585434	Lm	40	0.00	1.00	60.00	18.00	10.00	4.00	14.00	7445183.00	192.00	67838.00
Se4	GCF_001441225.1	ERR1102444	Lm	40	3.00	1.00	60.00	13.00	7.00	3.00	10.00	7801871.00	214.00	72261.00
Se5	GCF_001535315.2	SRR1166853	Lm	40	2.00	0.67	60.00	13.00	8.00	9.00	17.00	7763779.00	199.00	82661.00
Se6	GCF_000430145.2	SRR2650300	Lm	40	0.00	1.00	60.00	12.00	10.00	7.00	17.00	7820085.00	198.00	88923.00
Se7	GCF_000430085.2	SRR1207546	Lm	40	6.00	1.00	60.00	18.00	17.00	4.00	21.00	7841112.00	196.00	82044.00
Se8	GCF_000487935.2	SRR3223762	Lm	40	0.00	1.00	60.00	16.00	16.00	15.00	31.00	7925546.00	203.00	81720.00
Median			Lm	40	1.50	1.00	60.00	17.00	11.00	6.00	17.50	7782825.00	198.50	78684.50
Minimum			Lm	40	0.00	0.67	60.00	12.00			10.00	7445183.00	188.00	67838.00
Maximum			Lm	40	6.00	1.00	60.00	20.00			31.00	7925546.00	214.00	88923.00
Se1	GCF_000623095.1	SRR1520085	Lm	50	2.00	1.00	50.00	33.00	21.00	24.00	45.00	7609137.00	327.00	49817.00
Se2	GCF_000272715.4	ERR1599704	Lm	50	1.00	1.00	50.00	32.00	20.00	22.00	42.00	7719475.00	378.00	38195.00
Se3	GCF_000341425.1	SRR2585434	Lm	50	0.00	1.00	50.00	32.00	21.00	18.00	39.00	7437981.00	301.00	46071.00
Se4	GCF_001441225.1	ERR1102444	Lm	50	3.00	1.00	50.00	39.00	18.00	22.00	40.00	7793508.00	329.00	43457.00
Se5	GCF_001535315.2	SRR1166853	Lm	50	2.00	0.61	50.00	30.00	18.00	20.00	38.00	7753830.00	368.00	38438.00
Se6	GCF_000430145.2	SRR2650300	Lm	50	0.00	1.00	50.00	35.00	21.00	19.00	40.00	7811457.00	390.00	42979.00
Se7	GCF_000430085.2	SRR1207546	Lm	50	6.00	1.00	50.00	34.00	23.00	16.00	39.00	7837616.00	344.00	41124.00
Se8	GCF_000487935.2	SRR3223762	Lm	50	0.00	1.00	50.00	26.00	16.00	31.00	47.00	7915881.00	355.00	38805.00
Median			Lm	50	1.50	1.00	50.00	32.50	20.50	21.00	40.00	7773669.00	349.50	42051.50
Minimum			Lm	50	0.00	0.61	50.00	26.00			38.00	7437981.00	301.00	38195.00
Maximum			Lm	50	6.00	1.00	50.00	39.00			47.00	7915881.00	390.00	49817.00
Se1	GCF_000623095.1	SRR6244083	Ec	10	2.00	1.00	90.61	8.00	2.00	0.00	2.00	6517813.00	2357.00	131481.00
Se2	GCF_000272715.4	SRR3144198	Ec	10	1.00	1.00	90.56	9.00	1.00	0.00	1.00	6910316.00	2528.00	124644.00
Se3	GCF_000341425.1	SRR5438375	Ec	10	0.00	1.00	90.59	3.00	1.00	1.00	2.00	6477640.00	2359.00	152766.00
Se4	GCF_001441225.1	SRR5928789	Ec	10	3.00	1.00	90.70	2.00	0.00	0.00	0.00	6879589.00	2440.00	143743.00
Se5	GCF_001535315.2	SRR6080586	Ec	10	2.00	0.69	90.59	4.00	0.00	2.00	2.00	6903742.00	2455.00	114562.00
Se6	GCF_000430145.2	SRR6978933	Ec	10	0.00	1.00	90.60	1.00	0.00	0.00	0.00	7065545.00	2575.00	82061.00
Se7	GCF_000430085.2	SRR4054802	Ec	10	6.00	1.00	90.52	7.00	0.00	0.00	0.00	6837454.00	2472.00	137383.00
Se8	GCF_000487935.2	SRR7135396	Ec	10	0.00	1.00	90.61	3.00	0.00	3.00	3.00	7125182.00	2512.00	147386.00
Median			Ec	10	1.50	1.00	90.60	3.50	0.00	0.00	1.50	6891665.50	2463.50	134432.00
Minimum			Ec	10	0.00	0.69	90.52	1.00			0.00	6477640.00	2357.00	82061.00
Maximum			Ec	10	6.00	1.00	90.70	9.00			3.00	7125182.00	2575.00	152766.00
Se1	GCF_000623095.1	SRR6244083	Ec	20	2.00	1.00	81.18	14.00	9.00	19.00	28.00	8255452.00	2908.00	14080.00
Se2	GCF_000272715.4	SRR3144198	Ec	20	1.00	1.00	81.16	15.00	12.00	19.00	31.00	8718144.00	3011.00	12500.00
Se3	GCF_000341425.1	SRR5438375	Ec	20	0.00	1.00	81.13	12.00	11.00	17.00	28.00	8350777.00	3062.00	16660.00
Se4	GCF_001441225.1	SRR5928789	Ec	20	3.00	1.00	81.39	14.00	9.00	11.00	20.00	8724512.00	2985.00	14877.00
Se5	GCF_001535315.2	SRR6080586	Ec	20	2.00	0.63	81.15	10.00	13.00	14.00	27.00	8650048.00	2833.00	13719.00
Se6	GCF_000430145.2	SRR6978933	Ec	20	0.00	1.00	81.22	10.00	9.00	22.00	31.00	8956038.00	3172.00	11744.00
Se7	GCF_000430085.2	SRR4054802	Ec	20	6.00	1.00	81.05	13.00	8.00	5.00	13.00	8727159.00	3085.00	20841.00
Se8	GCF_000487935.2	SRR7135396	Ec	20	0.00	1.00	81.22	25.00	3.00	12.00	15.00	8879408.00	2731.00	20349.00
Median			Ec	20	1.50	1.00	81.17	13.50	9.00	15.50	27.50	8721328.00	2998.00	14478.50
Minimum			Ec	20	0.00	0.63	81.05	10.00			13.00	8255452.00	2731.00	11744.00
Maximum			Ec	20	6.00	1.00	81.39	25.00			31.00	8956038.00	3172.00	20841.00
Se1	GCF_000623095.1	SRR6244083	Ec	30	2.00	1.00	71.77	25.00	15.00	19.00	34.00	8941997.00	2014.00	9643.00
Se2	GCF_000272715.4	SRR3144198	Ec	30	1.00	1.00	71.70	32.00	17.00	19.00	36.00	9373482.00	1957.00	9515.00
Se3	GCF_000341425.1	SRR5438375	Ec	30	0.00	1.00	71.65	20.00	17.00	24.00	41.00	9113696.00	2234.00	8609.00
Se4	GCF_001441225.1	SRR5928789	Ec	30	3.00	1.00	72.08	23.00	19.00	19.00	38.00	9386076.00	1969.00	10192.00
Se5	GCF_001535315.2	SRR6080586	Ec	30	3.00	0.65	71.78	17.00	18.00	20.00	38.00	9255303.00	1863.00	10938.00
Se6	GCF_000430145.2	SRR6978933	Ec	30	0.00	1.00	71.83	20.00	20.00	24.00	44.00	9616991.00	2143.00	9934.00
Se7	GCF_000430085.2	SRR4054802	Ec	30	6.00	1.00	71.65	27.00	14.00	20.00	34.00	9550554.00	2267.00	8533.00
Se8	GCF_000487935.2	SRR7135396	Ec	30	0.00	1.00	71.79	23.00	20.00	21.00	41.00	9525743.00	1863.00	11499.00
Median			Ec	30	1.50	1.00	71.78	23.00	17.50	20.00	38.00	9379779.00	1991.50	9788.50
Minimum			Ec	30	0.00	0.65	71.65	17.00			34.00	8941997.00	1863.00	8533.00
Maximum			Ec	30	6.00	1.00	72.08	32.00			44.00	9616991.00	2267.00	11499.00
Se1	GCF_000623095.1	SRR6244083	Ec	40	2.00	1.00	62.37	52.00	18.00	17.00	35.00	9121826.00	1199.00	14874.00
Se2	GCF_000272715.4	SRR3144198	Ec	40	1.00	1.00	62.29	52.00	25.00	22.00	47.00	9527459.00	1168.00	15872.00
Se3	GCF_000341425.1	SRR5438375	Ec	40	0.00	1.00	62.24	47.00	14.00	17.00	31.00	9342299.00	1413.00	12804.00
Se4	GCF_001441225.1	SRR5928789	Ec	40	3.00	1.00	62.77	50.00	18.00	22.00	40.00	9549147.00	1195.00	16041.00
Se5	GCF_001535315.2	SRR6080586	Ec	40	3.00	0.66	62.36	44.00	28.00	17.00	45.00	9405184.00	1095.00	17661.00
Se6	GCF_000430145.2	SRR6978933	Ec	40	4.00	1.00	62.47	53.00	26.00	16.00	42.00	9796789.00	1309.00	14964.00
Se7	GCF_000430085.2	SRR4054802	Ec	40	6.00	1.00	62.21	59.00	24.00	23.00	47.00	9762495.00	1431.00	13375.00
Se8	GCF_000487935.2	SRR7135396	Ec	40	1.00	1.00	62.40	61.00	12.00	24.00	36.00	9665299.00	1120.00	16487.00
Median			Ec	40	2.50	1.00	62.37	52.00	21.00	19.50	41.00	9538303.00	1197.00	15418.00
Minimum			Ec	40	0.00	0.66	62.21	44.00			31.00	9121826.00	1095.00	12804.00
Maximum			Ec	40	6.00	1.00	62.77	61.00			47.00	9796789.00	1431.00	17661.00
Se1	GCF_000623095.1	SRR6244083	Ec	50	10.00	1.00	52.97	101.00	29.00	34.00	63.00	9155899.00	1006.00	17141.00
Se2	GCF_000272715.4	SRR3144198	Ec	50	7.00	1.00	52.89	115.00	20.00	25.00	45.00	9556550.00	925.00	19096.00
Se3	GCF_000341425.1	SRR5438375	Ec	50	3.00	1.00	52.77	90.00	28.00	33.00	61.00	9395880.00	1082.00	15390.00
Se4	GCF_001441225.1	SRR5928789	Ec	50	6.00	1.00	53.53	110.00	23.00	27.00	50.00	9578499.00	993.00	18436.00
Se5	GCF_001535315.2	SRR6080586	Ec	50	8.00	0.69	52.89	100.00	21.00	26.00	47.00	9416340.00	900.00	18937.00
Se6	GCF_000430145.2	SRR6978933	Ec	50	4.00	1.00	53.13	101.00	23.00	21.00	44.00	9836988.00	1013.00	17544.00
Se7	GCF_000430085.2	SRR4054802	Ec	50	6.00	1.00	52.68	99.00	27.00	25.00	52.00	9811835.00	1091.00	16025.00
Se8	GCF_000487935.2	SRR7135396	Ec	50	8.00	1.00	53.00	116.00	20.00	28.00	48.00	9687087.00	933.00	18585.00
Median			Ec	50	6.50	1.00	52.93	101.00	23.00	26.50	49.00	9567524.50	999.50	17990.00
Minimum			Ec	50	3.00	0.69	52.68	90.00			44.00	9155899.00	900.00	15390.00
Maximum			Ec	50	10.00	1.00	53.53	116.00			63.00	9836988.00	1091.00	19096.00

Note: Lm=*Listeria monocytogenes*, Se=*Salmonella enterica*, Ec=*Escherichia coli*

Table S2: Results of SNP pipeline and core-genome multi locus sequence typing analyses. (continued)

	Subject	Contaminant	Contaminant Type	Percent Contamination	SNPs	Bootstrap Support	Percent Reads Mapped	Alleles	Missing Alleles	Partial Alleles	Missing and Partial Alleles	Length	Number Contigs	N50
Ec1	GCF_003017975.1	GCF_003017975.1	Self	0	0.00	0.76	100.00	26.00	2.00	1.00	3.00	5093254.00	219.00	99838.00
Ec2	GCF_000986765.1	GCF_000986765.1	Self	0	3.00	0.87	100.00	2.00	1.00	1.00	2.00	5251073.00	200.00	107123.00
Ec3	GCF_002554485.1	GCF_002554485.1	Self	0	3.00	0.89	100.00	1.00	1.00	0.00	1.00	5272182.00	207.00	111221.00
Ec4	GCF_002209105.2	GCF_002209105.2	Self	0	9.00	1.00	100.00	4.00	2.00	0.00	2.00	5268936.00	187.00	100026.00
Ec5	GCF_003018035.1	GCF_003018035.1	Self	0	3.00	0.98	100.00	8.00	1.00	2.00	3.00	5468773.00	306.00	91532.00
Ec6	GCF_003018795.1	GCF_003018795.1	Self	0	2.00	1.00	100.00	0.00	1.00	0.00	1.00	5311028.00	276.00	114092.00
Ec7	GCF_001890285.1	GCF_001890285.1	Self	0	6.00	1.00	100.00	0.00	0.00	0.00	0.00	5278479.00	211.00	142507.00
Ec8	GCF_003112185.1	GCF_003112185.1	Self	0	5.00	0.93	100.00	3.00	0.00	0.00	0.00	5332324.00	272.00	104154.00
Median			Self	0	3.00	0.96	100.00	2.50	1.00	0.00	1.50	5275330.50	215.00	105638.50
Minimum			Self	0	0.00	0.76	100.00	0.00			0.00	5093254.00	187.00	91532.00
Maximum			Self	0	9.00	1.00	100.00	26.00			3.00	5468773.00	306.00	142507.00
Ec1	GCF_003017975.1	GCF_003017975.1	Self	10	0.00	0.63	100.00	26.00	1.00	2.00	3.00	5088029.00	219.00	91459.00
Ec2	GCF_000986765.1	GCF_000986765.1	Self	10	3.00	0.85	100.00	2.00	1.00	1.00	2.00	5252088.00	218.00	103971.00
Ec3	GCF_002554485.1	GCF_002554485.1	Self	10	3.00	0.89	100.00	1.00	1.00	0.00	1.00	5266757.00	217.00	95186.00
Ec4	GCF_002209105.2	GCF_002209105.2	Self	10	9.00	1.00	100.00	5.00	2.00	0.00	2.00	5265915.00	195.00	84764.00
Ec5	GCF_003018035.1	GCF_003018035.1	Self	10	3.00	0.98	100.00	8.00	1.00	2.00	3.00	5466936.00	315.00	79172.00
Ec6	GCF_003018795.1	GCF_003018795.1	Self	10	2.00	1.00	100.00	0.00	1.00	0.00	1.00	5311845.00	271.00	100876.00
Ec7	GCF_001890285.1	GCF_001890285.1	Self	10	6.00	1.00	100.00	1.00	0.00	0.00	0.00	5272525.00	225.00	132974.00
Ec8	GCF_003112185.1	GCF_003112185.1	Self	10	5.00	0.94	100.00	3.00	0.00	0.00	0.00	5331776.00	274.00	104154.00
Median			Self	10	3.00	0.96	100.00	2.50	1.00	0.00	1.50	5269641.00	222.00	98031.00
Minimum			Self	10	0.00	0.63	100.00	0.00			0.00	5088029.00	195.00	79172.00
Maximum			Self	10	9.00	1.00	100.00	26.00			3.00	5466936.00	315.00	132974.00
Ec1	GCF_003017975.1	GCF_003017975.1	Self	20	0.00	0.63	100.00	26.00	1.00	1.00	2.00	5090651.00	220.00	90911.00
Ec2	GCF_000986765.1	GCF_000986765.1	Self	20	3.00	0.86	100.00	4.00	1.00	2.00	3.00	5248390.00	219.00	102266.00
Ec3	GCF_002554485.1	GCF_002554485.1	Self	20	3.00	0.83	100.00	2.00	1.00	0.00	1.00	5266259.00	222.00	88166.00
Ec4	GCF_002209105.2	GCF_002209105.2	Self	20	9.00	1.00	100.00	6.00	2.00	1.00	3.00	5262180.00	194.00	91368.00
Ec5	GCF_003018035.1	GCF_003018035.1	Self	20	3.00	0.97	100.00	11.00	1.00	3.00	4.00	5458850.00	313.00	73440.00
Ec6	GCF_003018795.1	GCF_003018795.1	Self	20	2.00	1.00	100.00	2.00	1.00	0.00	1.00	5312101.00	291.00	103964.00
Ec7	GCF_001890285.1	GCF_001890285.1	Self	20	6.00	1.00	100.00	1.00	0.00	1.00	1.00	5271854.00	227.00	109602.00
Ec8	GCF_003112185.1	GCF_003112185.1	Self	20	5.00	0.93	100.00	4.00	0.00	0.00	0.00	5326390.00	297.00	92072.00
Median			Self	20	3.00	0.95	100.00	4.00	1.00	1.00	1.50	5269056.50	224.50	91720.00
Minimum			Self	20	0.00	0.63	100.00	1.00			0.00	5090651.00	194.00	73440.00
Maximum			Self	20	9.00	1.00	100.00	26.00			4.00	5458850.00	313.00	109602.00
Ec1	GCF_003017975.1	GCF_003017975.1	Self	30	0.00	0.71	100.00	26.00	1.00	2.00	3.00	5096775.00	252.00	72202.00
Ec2	GCF_000986765.1	GCF_000986765.1	Self	30	3.00	0.94	100.00	4.00	1.00	2.00	3.00	5245158.00	238.00	78194.00
Ec3	GCF_002554485.1	GCF_002554485.1	Self	30	3.00	0.88	100.00	2.00	2.00	1.00	3.00	5274609.00	226.00	88591.00
Ec4	GCF_002209105.2	GCF_002209105.2	Self	30	9.00	1.00	100.00	7.00	2.00	0.00	2.00	5263530.00	213.00	91365.00
Ec5	GCF_003018035.1	GCF_003018035.1	Self	30	3.00	0.99	100.00	11.00	2.00	2.00	4.00	5455858.00	340.00	60168.00
Ec6	GCF_003018795.1	GCF_003018795.1	Self	30	2.00	1.00	100.00	2.00	1.00	1.00	2.00	5310038.00	293.00	92448.00
Ec7	GCF_001890285.1	GCF_001890285.1	Self	30	6.00	1.00	100.00	5.00	1.00	2.00	3.00	5269248.00	235.00	99482.00
Ec8	GCF_003112185.1	GCF_003112185.1	Self	30	5.00	0.95	100.00	4.00	0.00	0.00	0.00	5322335.00	294.00	92072.00
Median			Self	30	3.00	0.97	100.00	4.50	1.00	1.50	3.00	5271928.50	245.00	89978.00
Minimum			Self	30	0.00	0.71	100.00	2.00			0.00	5096775.00	213.00	60168.00
Maximum			Self	30	9.00	1.00	100.00	26.00			4.00	5455858.00	340.00	99482.00
Ec1	GCF_003017975.1	GCF_003017975.1	Self	40	0.00	0.67	100.00	28.00	3.00	2.00	5.00	5090871.00	246.00	71910.00
Ec2	GCF_000986765.1	GCF_000986765.1	Self	40	3.00	0.93	100.00	2.00	3.00	5.00	8.00	5249480.00	234.00	77207.00
Ec3	GCF_002554485.1	GCF_002554485.1	Self	40	3.00	0.95	100.00	2.00	0.00	1.00	1.00	5274089.00	242.00	84985.00
Ec4	GCF_002209105.2	GCF_002209105.2	Self	40	9.00	1.00	100.00	8.00	3.00	0.00	3.00	5259825.00	232.00	76899.00
Ec5	GCF_003018035.1	GCF_003018035.1	Self	40	3.00	0.98	100.00	8.00	3.00	3.00	6.00	5451324.00	372.00	56895.00
Ec6	GCF_003018795.1	GCF_003018795.1	Self	40	2.00	1.00	100.00	4.00	2.00	1.00	3.00	5305464.00	342.00	67649.00
Ec7	GCF_001890285.1	GCF_001890285.1	Self	40	6.00	1.00	100.00	3.00	1.00	1.00	2.00	5280846.00	235.00	109262.00
Ec8	GCF_003112185.1	GCF_003112185.1	Self	40	5.00	0.91	100.00	6.00	1.00	2.00	3.00	5323437.00	329.00	64342.00
Median			Self	40	3.00	0.97	100.00	5.00	2.50	1.50	3.00	5277467.50	244.00	74404.50
Minimum			Self	40	0.00	0.67	100.00	2.00			1.00	5090871.00	232.00	56895.00
Maximum			Self	40	9.00	1.00	100.00	28.00			8.00	5451324.00	372.00	109262.00
Ec1	GCF_003017975.1	GCF_003017975.1	Self	50	0.00	0.59	100.00	31.00	3.00	2.00	5.00	5090462.00	243.00	78192.00
Ec2	GCF_000986765.1	GCF_000986765.1	Self	50	3.00	0.86	100.00	5.00	1.00	2.00	3.00	5246175.00	242.00	75192.00
Ec3	GCF_002554485.1	GCF_002554485.1	Self	50	3.00	0.87	100.00	5.00	3.00	3.00	6.00	5266852.00	257.00	74286.00
Ec4	GCF_002209105.2	GCF_002209105.2	Self	50	9.00	1.00	100.00	5.00	2.00	4.00	6.00	5265280.00	229.00	73603.00
Ec5	GCF_003018035.1	GCF_003018035.1	Self	50	3.00	1.00	100.00	11.00	3.00	7.00	10.00	5456432.00	379.00	55370.00
Ec6	GCF_003018795.1	GCF_003018795.1	Self	50	2.00	0.99	100.00	1.00	1.00	3.00	4.00	5308789.00	341.00	55083.00
Ec7	GCF_001890285.1	GCF_001890285.1	Self	50	6.00	1.00	100.00	3.00	0.00	2.00	2.00	5270484.00	243.00	109430.00
Ec8	GCF_003112185.1	GCF_003112185.1	Self	50	5.00	0.94	100.00	5.00	1.00	2.00	3.00	5323646.00	322.00	67899.00
Median			Self	50	3.00	0.97	100.00	5.00	1.50	2.50	4.50	5268668.00	250.00	73944.50
Minimum			Self	50	0.00	0.59	100.00	1.00			2.00	5090462.00	229.00	55083.00
Maximum			Self	50	9.00	1.00	100.00	31.00			10.00	5456432.00	379.00	109430.00
Ec1	GCF_003017975.1	SRR7133502	0.05	10	0.00	0.65	99.71	26.00	1.00	1.00	2.00	5148278.00	275.00	104304.00
Ec2	GCF_000986765.1	SRR6467535	0.05	10	3.00	0.90	99.57	2.00	1.00	0.00	1.00	5347456.00	316.00	103337.00
Ec3	GCF_002554485.1	SRR5283470	0.05	10	3.00	0.87	99.61	1.00	1.00	0.00	1.00	5356095.00	317.00	111221.00
Ec4	GCF_002209105.2	SRR7251472	0.05	10	9.00	1.00	99.83	4.00	2.00	0.00	2.00	5309672.00	239.00	100672.00
Ec5	GCF_003018035.1	SRR7244127	0.05	10	3.00	0.99	94.76	7.00	1.00	1.00	2.00	5511840.00	387.00	92078.00
Ec6	GCF_003018795.1	SRR7195070	0.05	10	2.00	1.00	99.75	0.00	1.00	0.00	1.00	5358807.00	333.00	114781.00
Ec7	GCF_001890285.1	SRR2187165	0.05	10	6.00	1.00	99.78	0.00	0.00	0.00	0.00	5321204.00	268.00	146132.00
Ec8	GCF_003112185.1	SRR7280413	0.05	10	5.00	0.93	99.62	3.00	0.00	0.00	0.00	5413270.00	377.00	100982.00
Median			0.05	10	3.00	0.96	99.67	2.50	1.00	0.00	1.00	5351775.50	316.50	103820.50
Minimum			0.05	10	0.00	0.65	94.76	0.00			0.00	5148278.00	239.00	92078.00
Maximum			0.05	10	9.00	1.00	99.83	26.00			2.00	5511840.00	387.00	146132.00
Ec1	GCF_003017975.1	SRR7133502	0.05	20	0.00	0.54	99.44	27.00	2.00	1.00	3.00	5198302.00	305.00	89939.00
Ec2	GCF_000986765.1	SRR6467535	0.05	20	3.00	0.88	99.14	1.00	0.00	1.00	1.00	5417446.00	348.00	102266.00
Ec3	GCF_002554485.1	SRR5283470	0.05	20	3.00	0.77	99.26	2.00	1.00	1.00	2.00	5423191.00	359.00	88166.00
Ec4	GCF_002209105.2	SRR7251472	0.05	20	9.00	1.00	99.65	3.00	0.00	0.00	0.00	5323861.00	286.00	88194.00
Ec5	GCF_003018035.1	SRR7244127	0.05	20	3.00	1.00	89.54	7.00	1.00	1.00	2.00	5605280.00	511.00	58098.00
Ec6	GCF_003018795.1	SRR7195070	0.05	20	2.00	1.00	99.50	0.00	1.00	0.00	1.00	5402804.00	377.00	79859.00
Ec7	GCF_001890285.1	SRR2187165	0.05	20	6.00	0.98	99.54	0.00	0.00	3.00	3.00	5377265.00	355.00	62361.00
Ec8	GCF_003112185.1	SRR7280413	0.05	20	5.00	0.91	99.23	3.00	2.00	1.00	3.00	5488167.00	431.00	70213.00
Median			0.05	20	3.00	0.95	99.35	2.50	1.00	1.00	2.00	5410125.00	357.00	84012.50
Minimum			0.05	20	0.00	0.54	89.54	0.00			0.00	5198302.00	286.00	58098.00
Maximum			0.05	20	9.00	1.00	99.65	27.00			3.00	5605280.00	511.00	102266.00
Ec1	GCF_003017975.1	SRR7133502	0.05	30	1.00	0.48	99.11	28.00	1.00	2.00	3.00	5229362.00	364.00	58564.00
Ec2	GCF_000986765.1	SRR6467535	0.05	30	4.00	0.72	98.72	2.00	1.00	4.00	5.00	5448487.00	389.00	61347.00
Ec3	GCF_002554485.1	SRR5283470	0.05	30	3.00	0.86	98.85	2.00	1.00	1.00	2.00	5453442.00	382.00	83364.00
Ec4	GCF_002209105.2	SRR7251472	0.05	30	10.00	1.00	99.47	5.00	3.00	5.00	8.00	5328280.00	355.00	84764.00
Ec5	GCF_003018035.1	SRR7244127	0.05	30	3.00	1.00	84.31	17.00	2.00	6.00	8.00	5665507.00	648.00	33595.00
Ec6	GCF_003018795.1	SRR7195070	0.05	30	2.00	0.99	99.24	9.00	1.00	4.00	5.00	5438196.00	444.00	48292.00
Ec7	GCF_001890285.1	SRR2187165	0.05	30	6.00	0.99	99.28	5.00	7.00	15.00	22.00	5393384.00	508.00	25802.00
Ec8	GCF_003112185.1	SRR7280413	0.05	30	5.00	0.95	98.86	6.00	2.00	2.00	4.00	5504601.00	471.00	48292.00
Median			0.05	30	3.50	0.97	98.99	5.50	1.50	4.00	5.00	5443341.50	416.50	53428.00
Minimum			0.05	30	1.00	0.48	84.31	2.00			2.00	5229362.00	355.00	25802.00
Maximum			0.05	30	10.00	1.00	99.47	28.00			22.00	5665507.00	648.00	84764.00
Ec1	GCF_003017975.1	SRR7133502	0.05	40	1.00	0.46	98.86	28.00	3.00	3.00	6.00	5232360.00	414.00	44533.00
Ec2	GCF_000986765.1	SRR6467535	0.05	40	3.00	0.00	98.31	8.00	2.00	7.00	9.00	5459191.00	488.00	38150.00
Ec3	GCF_002554485.1	SRR5283470	0.05	40	1.00	0.68	98.45	4.00	2.00	4.00	6.00	5464212.00	435.00	65458.00
Ec4	GCF_002209105.2	SRR7251472	0.05	40	10.00	1.00	99.30	7.00	4.00	5.00	9.00	5325736.00	412.00	74312.00
Ec5	GCF_003018035.1	SRR7244127	0.05	40	3.00	0.98	79.08	25.00	4.00	7.00	11.00	5690807.00	713.00	29035.00
Ec6	GCF_003018795.1	SRR7195070	0.05	40	4.00	0.99	98.97	13.00	2.00	8.00	10.00	5450501.00	577.00	28049.00
Ec7	GCF_001890285.1	SRR2187165	0.05	40	10.00	0.79	99.07	17.00	15.00	34.00	49.00	5386433.00	889.00	10500.00
Ec8	GCF_003112185.1	SRR7280413	0.05	40	7.00	0.97	98.49	14.00	6.00	8.00	14.00	5510655.00	619.00	26797.00
Median			0.05	40	3.50	0.88	98.68	13.50	3.50	7.00	9.50	5454846.00	532.50	33592.50
Minimum			0.05	40	1.00	0.00	79.08	4.00			6.00	5232360.00	412.00	10500.00
Maximum			0.05	40	10.00	1.00	99.30	28.00			49.00	5690807.00	889.00	74312.00
Ec1	GCF_003017975.1	SRR7133502	0.05	50	2.00	0.42	98.56	34.00	2.00	8.00	10.00	5236562.00	436.00	37017.00
Ec2	GCF_000986765.1	SRR6467535	0.05	50	11.00	0.00	97.87	13.00	1.00	6.00	7.00	5454831.00	497.00	37634.00
Ec3	GCF_002554485.1	SRR5283470	0.05	50	12.00	0.00	98.10	11.00	1.00	6.00	7.00	5466151.00	443.00	51953.00
Ec4	GCF_002209105.2	SRR7251472	0.05	50	14.00	1.00	99.10	11.00	4.00	3.00	7.00	5311441.00	437.00	73500.00
Ec5	GCF_003018035.1	SRR7244127	0.05	50	3.00	0.98	73.83	39.00	6.00	11.00	17.00	5716768.00	746.00	26116.00
Ec6	GCF_003018795.1	SRR7195070	0.05	50	7.00	0.84	98.74	18.00	5.00	8.00	13.00	5446751.00	620.00	25745.00
Ec7	GCF_001890285.1	SRR2187165	0.05	50	13.00	0.00	98.82	30.00	16.00	47.00	63.00	5381635.00	971.00	9777.00
Ec8	GCF_003112185.1	SRR7280413	0.05	50	12.00	0.81	98.07	31.00	4.00	8.00	12.00	5514368.00	631.00	25730.00
Median			0.05	50	11.50	0.62	98.33	24.00	4.00	8.00	11.00	5450791.00	558.50	31566.50
Minimum			0.05	50	2.00	0.00	73.83	11.00			7.00	5236562.00	436.00	9777.00
Maximum			0.05	50	14.00	1.00	99.10	39.00			63.00	5716768.00	971.00	73500.00
Ec1	GCF_003017975.1	SRR5181518	0.5	10	0.00	0.66	99.12	28.00	1.00	2.00	3.00	5282807.00	461.00	92323.00
Ec2	GCF_000986765.1	SRR3438053	0.5	10	3.00	0.94	99.09	2.00	1.00	1.00	2.00	5451107.00	456.00	87594.00
Ec3	GCF_002554485.1	SRR6930597	0.5	10	3.00	0.89	98.96	3.00	1.00	0.00	1.00	5483941.00	474.00	88166.00
Ec4	GCF_002209105.2	SRR2965629	0.5	10	8.00	1.00	99.31	8.00	1.00	1.00	2.00	5419993.00	382.00	81091.00
Ec5	GCF_003018035.1	SRR7130338	0.5	10	3.00	0.96	99.15	9.00	2.00	1.00	3.00	5658678.00	548.00	68684.00
Ec6	GCF_003018795.1	SRR6930654	0.5	10	2.00	1.00	99.09	3.00	1.00	2.00	3.00	5505203.00	504.00	82552.00
Ec7	GCF_001890285.1	SRR6512620	0.5	10	6.00	0.99	99.09	1.00	0.00	0.00	0.00	5466960.00	436.00	120943.00
Ec8	GCF_003112185.1	SRR5181518	0.5	10	5.00	0.94	99.25	10.00	0.00	0.00	0.00	5496469.00	478.00	82595.00
Median			0.5	10	3.00	0.95	99.11	5.50	1.00	1.00	2.00	5475450.50	467.50	85094.50
Minimum			0.5	10	0.00	0.66	98.96	1.00			0.00	5282807.00	382.00	68684.00
Maximum			0.5	10	8.00	1.00	99.31	28.00			3.00	5658678.00	548.00	120943.00
Ec1	GCF_003017975.1	SRR5181518	0.5	20	0.00	0.63	98.24	58.00	9.00	45.00	54.00	5415280.00	896.00	13082.00
Ec2	GCF_000986765.1	SRR3438053	0.5	20	3.00	0.88	98.15	27.00	6.00	29.00	35.00	5591729.00	785.00	17666.00
Ec3	GCF_002554485.1	SRR6930597	0.5	20	4.00	0.81	97.98	16.00	6.00	24.00	30.00	5661410.00	836.00	20279.00
Ec4	GCF_002209105.2	SRR2965629	0.5	20	7.00	1.00	98.67	34.00	11.00	24.00	35.00	5504261.00	774.00	16375.00
Ec5	GCF_003018035.1	SRR7130338	0.5	20	3.00	0.97	98.36	52.00	17.00	59.00	76.00	5759512.00	1146.00	10066.00
Ec6	GCF_003018795.1	SRR6930654	0.5	20	2.00	1.00	98.20	41.00	14.00	46.00	60.00	5632319.00	1079.00	11575.00
Ec7	GCF_001890285.1	SRR6512620	0.5	20	6.00	0.98	98.13	7.00	4.00	8.00	12.00	5643272.00	689.00	34567.00
Ec8	GCF_003112185.1	SRR5181518	0.5	20	5.00	0.91	98.51	50.00	17.00	60.00	77.00	5581145.00	1099.00	9583.00
Median			0.5	20	3.50	0.94	98.22	37.50	10.00	37.00	44.50	5612024.00	866.00	14728.50
Minimum			0.5	20	0.00	0.63	97.98	7.00			12.00	5415280.00	689.00	9583.00
Maximum			0.5	20	7.00	1.00	98.67	58.00			77.00	5759512.00	1146.00	34567.00
Ec1	GCF_003017975.1	SRR5181518	0.5	30	1.00	0.57	97.39	108.00	49.00	193.00	242.00	5311809.00	2228.00	3625.00
Ec2	GCF_000986765.1	SRR3438053	0.5	30	5.00	0.68	97.26	63.00	31.00	118.00	149.00	5558225.00	1776.00	5339.00
Ec3	GCF_002554485.1	SRR6930597	0.5	30	3.00	0.83	96.89	69.00	27.00	104.00	131.00	5646871.00	1762.00	5790.00
Ec4	GCF_002209105.2	SRR2965629	0.5	30	9.00	1.00	98.03	65.00	35.00	127.00	162.00	5396118.00	1854.00	4941.00
Ec5	GCF_003018035.1	SRR7130338	0.5	30	7.00	0.86	97.52	88.00	57.00	215.00	272.00	5573018.00	2602.00	3190.00
Ec6	GCF_003018795.1	SRR6930654	0.5	30	2.00	1.00	97.31	98.00	51.00	170.00	221.00	5500153.00	2384.00	3569.00
Ec7	GCF_001890285.1	SRR6512620	0.5	30	7.00	0.94	97.27	28.00	9.00	35.00	44.00	5695968.00	1001.00	13427.00
Ec8	GCF_003112185.1	SRR5181518	0.5	30	5.00	0.95	97.77	104.00	50.00	185.00	235.00	5456753.00	2415.00	3500.00
Median			0.5	30	5.00	0.90	97.35	78.50	42.00	148.50	191.50	5529189.00	2041.00	4283.00
Minimum			0.5	30	1.00	0.57	96.89	28.00			44.00	5311809.00	1001.00	3190.00
Maximum			0.5	30	9.00	1.00	98.03	108.00			272.00	5695968.00	2602.00	13427.00
Ec1	GCF_003017975.1	SRR5181518	0.5	40	13.00	0.57	96.45	206.00	59.00	244.00	303.00	5193383.00	2796.00	2693.00
Ec2	GCF_000986765.1	SRR3438053	0.5	40	16.00	0.56	96.39	116.00	45.00	155.00	200.00	5483326.00	2249.00	4282.00
Ec3	GCF_002554485.1	SRR6930597	0.5	40	12.00	0.87	95.86	123.00	36.00	157.00	193.00	5585644.00	2234.00	4469.00
Ec4	GCF_002209105.2	SRR2965629	0.5	40	16.00	1.00	97.34	131.00	49.00	172.00	221.00	5317797.00	2374.00	3695.00
Ec5	GCF_003018035.1	SRR7130338	0.5	40	20.00	0.78	96.72	219.00	51.00	289.00	340.00	5466515.00	3095.00	2459.00
Ec6	GCF_003018795.1	SRR6930654	0.5	40	22.00	1.00	96.43	180.00	62.00	244.00	306.00	5379253.00	2963.00	2602.00
Ec7	GCF_001890285.1	SRR6512620	0.5	40	6.00	0.96	96.31	85.00	21.00	55.00	76.00	5704286.00	1361.00	8131.00
Ec8	GCF_003112185.1	SRR5181518	0.5	40	20.00	0.95	97.03	180.00	68.00	263.00	331.00	5310464.00	2984.00	2535.00
Median			0.5	40	16.00	0.91	96.44	155.50	50.00	208.00	262.00	5422884.00	2585.00	3194.00
Minimum			0.5	40	6.00	0.56	95.86	85.00			76.00	5193383.00	1361.00	2459.00
Maximum			0.5	40	22.00	1.00	97.34	219.00			340.00	5704286.00	3095.00	8131.00
Ec1	GCF_003017975.1	SRR5181518	0.5	50	71.00	0.00	95.60	280.00	72.00	259.00	331.00	5159762.00	2958.00	2373.00
Ec2	GCF_000986765.1	SRR3438053	0.5	50	72.00	0.00	95.46	183.00	51.00	168.00	219.00	5481703.00	2427.00	3787.00
Ec3	GCF_002554485.1	SRR6930597	0.5	50	66.00	0.00	94.88	180.00	42.00	160.00	202.00	5560438.00	2386.00	3976.00
Ec4	GCF_002209105.2	SRR2965629	0.5	50	80.00	1.00	96.71	210.00	42.00	180.00	222.00	5299325.00	2492.00	3498.00
Ec5	GCF_003018035.1	SRR7130338	0.5	50	100.00	0.00	95.84	299.00	77.00	275.00	352.00	5443167.00	3173.00	2349.00
Ec6	GCF_003018795.1	SRR6930654	0.5	50	89.00	1.00	95.56	272.00	72.00	248.00	320.00	5336036.00	3035.00	2463.00
Ec7	GCF_001890285.1	SRR6512620	0.5	50	34.00	0.00	95.51	134.00	23.00	61.00	84.00	5710444.00	1508.00	7061.00
Ec8	GCF_003112185.1	SRR5181518	0.5	50	92.00	0.99	96.22	292.00	66.00	252.00	318.00	5308424.00	3102.00	2376.00
Median			0.5	50	76.00	0.00	95.58	241.00	58.50	214.00	270.00	5389601.50	2725.00	2980.50
Minimum			0.5	50	34.00	0.00	94.88	134.00			84.00	5159762.00	1508.00	2349.00
Maximum			0.5	50	100.00	1.00	96.71	299.00			352.00	5710444.00	3173.00	7061.00
Ec1	GCF_003017975.1	SRR3578956	5	10	0.00	0.60	96.00	39.00	1.00	1.00	2.00	5854162.00	1156.00	67439.00
Ec2	GCF_000986765.1	SRR7299301	5	10	3.00	0.93	94.53	5.00	2.00	2.00	4.00	6435034.00	1671.00	57316.00
Ec3	GCF_002554485.1	SRR7297675	5	10	3.00	0.83	94.59	12.00	1.00	1.00	2.00	6469808.00	1645.00	58097.00
Ec4	GCF_002209105.2	SRR5035023	5	10	9.00	1.00	95.61	16.00	3.00	1.00	4.00	6114751.00	1282.00	59378.00
Ec5	GCF_003018035.1	SRR7085315	5	10	3.00	0.99	95.96	22.00	1.00	2.00	3.00	6320320.00	1395.00	40671.00
Ec6	GCF_003018795.1	SRR7221692	5	10	2.00	1.00	90.51	1.00	1.00	1.00	2.00	7594933.00	3055.00	39605.00
Ec7	GCF_001890285.1	SRR2449168	5	10	6.00	0.97	90.56	2.00	0.00	3.00	3.00	7579990.00	2978.00	55275.00
Ec8	GCF_003112185.1	SRR2000282	5	10	5.00	0.93	95.66	25.00	0.00	2.00	2.00	6217808.00	1413.00	37987.00
Median			5	10	3.00	0.95	95.10	14.00	1.00	1.50	2.50	6377677.00	1529.00	56295.50
Minimum			5	10	0.00	0.60	90.51	1.00			2.00	5854162.00	1156.00	37987.00
Maximum			5	10	9.00	1.00	96.00	39.00			4.00	7594933.00	3055.00	67439.00
Ec1	GCF_003017975.1	SRR3578956	5	20	0.00	0.60	92.06	153.00	5.00	26.00	31.00	6748664.00	1902.00	11767.00
Ec2	GCF_000986765.1	SRR7299301	5	20	3.00	0.78	88.94	79.00	1.00	16.00	17.00	7489146.00	2111.00	15778.00
Ec3	GCF_002554485.1	SRR7297675	5	20	3.00	0.87	89.07	92.00	4.00	22.00	26.00	7494403.00	2033.00	13165.00
Ec4	GCF_002209105.2	SRR5035023	5	20	9.00	1.00	91.19	122.00	7.00	36.00	43.00	7211033.00	2260.00	8392.00
Ec5	GCF_003018035.1	SRR7085315	5	20	3.00	0.98	91.88	129.00	14.00	56.00	70.00	7354866.00	2389.00	7459.00
Ec6	GCF_003018795.1	SRR7221692	5	20	2.00	0.98	81.04	6.00	4.00	15.00	19.00	9702716.00	3689.00	5726.00
Ec7	GCF_001890285.1	SRR2449168	5	20	6.00	0.98	81.12	6.00	4.00	15.00	19.00	9543573.00	3382.00	7933.00
Ec8	GCF_003112185.1	SRR2000282	5	20	5.00	0.91	91.22	141.00	10.00	54.00	64.00	7386967.00	2596.00	6392.00
Median			5	20	3.00	0.95	90.13	107.00	4.50	24.00	28.50	7438056.50	2324.50	8162.50
Minimum			5	20	0.00	0.60	81.04	6.00			17.00	6748664.00	1902.00	5726.00
Maximum			5	20	9.00	1.00	92.06	153.00			70.00	9702716.00	3689.00	15778.00
Ec1	GCF_003017975.1	SRR3578956	5	30	3.00	0.69	88.07	152.00	34.00	207.00	241.00	7892343.00	4304.00	2574.00
Ec2	GCF_000986765.1	SRR7299301	5	30	6.00	0.87	83.47	96.00	36.00	193.00	229.00	8205927.00	3675.00	3438.00
Ec3	GCF_002554485.1	SRR7297675	5	30	3.00	0.89	83.74	86.00	24.00	191.00	215.00	8178307.00	3661.00	3733.00
Ec4	GCF_002209105.2	SRR5035023	5	30	10.00	1.00	86.76	114.00	45.00	131.00	176.00	8533909.00	3784.00	3560.00
Ec5	GCF_003018035.1	SRR7085315	5	30	6.00	0.88	87.82	103.00	42.00	160.00	202.00	8700770.00	4176.00	3136.00
Ec6	GCF_003018795.1	SRR7221692	5	30	2.00	1.00	71.55	22.00	9.00	19.00	28.00	10483974.00	2569.00	7732.00
Ec7	GCF_001890285.1	SRR2449168	5	30	6.00	1.00	71.67	15.00	3.00	19.00	22.00	10212604.00	2211.00	8933.00
Ec8	GCF_003112185.1	SRR2000282	5	30	11.00	0.91	86.97	110.00	40.00	119.00	159.00	8717643.00	3740.00	3536.00
Median			5	30	6.00	0.90	85.25	99.50	35.00	145.50	189.00	8617339.50	3707.50	3548.00
Minimum			5	30	2.00	0.69	71.55	15.00			22.00	7892343.00	2211.00	2574.00
Maximum			5	30	11.00	1.00	88.07	152.00			241.00	10483974.00	4304.00	8933.00
Ec1	GCF_003017975.1	SRR3578956	5	40	20.00	0.61	83.99	196.00	45.00	191.00	236.00	8590047.00	4145.00	3002.00
Ec2	GCF_000986765.1	SRR7299301	5	40	18.00	0.83	77.98	146.00	45.00	198.00	243.00	8662237.00	3711.00	4622.00
Ec3	GCF_002554485.1	SRR7297675	5	40	27.00	0.82	78.38	143.00	43.00	212.00	255.00	8592071.00	3551.00	4915.00
Ec4	GCF_002209105.2	SRR5035023	5	40	34.00	1.00	82.47	163.00	43.00	92.00	135.00	9031854.00	3115.00	4817.00
Ec5	GCF_003018035.1	SRR7085315	5	40	29.00	0.90	83.76	175.00	28.00	132.00	160.00	9207355.00	3505.00	4172.00
Ec6	GCF_003018795.1	SRR7221692	5	40	2.00	1.00	62.05	35.00	8.00	18.00	26.00	10679639.00	1658.00	12446.00
Ec7	GCF_001890285.1	SRR2449168	5	40	7.00	1.00	62.26	38.00	3.00	15.00	18.00	10362355.00	1474.00	14273.00
Ec8	GCF_003112185.1	SRR2000282	5	40	32.00	0.93	82.50	150.00	30.00	85.00	115.00	9109000.00	3007.00	4930.00
Median			5	40	23.50	0.92	80.43	148.00	36.50	112.00	147.50	9070427.00	3310.00	4866.00
Minimum			5	40	2.00	0.61	62.05	35.00			18.00	8590047.00	1474.00	3002.00
Maximum			5	40	34.00	1.00	83.99	196.00			255.00	10679639.00	4145.00	14273.00
Ec1	GCF_003017975.1	SRR3578956	5	50	146.00	0.00	80.00	306.00	52.00	171.00	223.00	8699731.00	4045.00	3256.00
Ec2	GCF_000986765.1	SRR7299301	5	50	108.00	0.63	72.46	245.00	45.00	210.00	255.00	8757154.00	3534.00	5013.00
Ec3	GCF_002554485.1	SRR7297675	5	50	117.00	0.79	73.02	270.00	29.00	210.00	239.00	8702795.00	3418.00	5349.00
Ec4	GCF_002209105.2	SRR5035023	5	50	142.00	1.00	78.05	259.00	31.00	81.00	112.00	9071931.00	3059.00	4772.00
Ec5	GCF_003018035.1	SRR7085315	5	50	214.00	0.00	79.80	299.00	38.00	124.00	162.00	9175368.00	3602.00	4179.00
Ec6	GCF_003018795.1	SRR7221692	5	50	10.00	0.99	52.59	81.00	14.00	13.00	27.00	10714901.00	1400.00	15752.00
Ec7	GCF_001890285.1	SRR2449168	5	50	13.00	0.99	52.80	62.00	12.00	19.00	31.00	10386420.00	1216.00	17633.00
Ec8	GCF_003112185.1	SRR2000282	5	50	187.00	0.95	78.18	268.00	30.00	100.00	130.00	9053444.00	3074.00	4868.00
Median			5	50	129.50	0.87	75.54	263.50	30.50	112.00	146.00	9062687.50	3246.00	4940.50
Minimum			5	50	10.00	0.00	52.59	62.00			27.00	8699731.00	1216.00	3256.00
Maximum			5	50	214.00	1.00	80.00	306.00			255.00	10714901.00	4045.00	17633.00
Ec1	GCF_003017975.1	SRR1220810	Lm	10	0.00	0.72	90.00	26.00	1.00	1.00	2.00	7432899.00	1996.00	44802.00
Ec2	GCF_000986765.1	SRR2646874	Lm	10	3.00	0.93	90.00	2.00	1.00	0.00	1.00	7619129.00	2061.00	51359.00
Ec3	GCF_002554485.1	SRR1575064	Lm	10	3.00	0.88	90.00	2.00	0.00	0.00	0.00	7605263.00	1987.00	51595.00
Ec4	GCF_002209105.2	SRR1562093	Lm	10	9.00	1.00	90.00	4.00	2.00	1.00	3.00	7585989.00	1931.00	48430.00
Ec5	GCF_003018035.1	SRR3535390	Lm	10	3.00	0.98	90.00	8.00	1.00	2.00	3.00	7915410.00	2010.00	41534.00
Ec6	GCF_003018795.1	SRR1055859	Lm	10	2.00	1.00	90.00	1.00	1.00	0.00	1.00	7722911.00	1977.00	47677.00
Ec7	GCF_001890285.1	SRR1506599	Lm	10	6.00	0.99	90.00	0.00	0.00	1.00	1.00	7667022.00	1958.00	83983.00
Ec8	GCF_003112185.1	SRR1217490	Lm	10	5.00	0.93	90.00	5.00	0.00	0.00	0.00	7744040.00	1959.00	47801.00
Median			Lm	10	3.00	0.96	90.00	3.00	1.00	0.50	1.00	7643075.50	1982.00	48115.50
Minimum			Lm	10	0.00	0.72	90.00	0.00			0.00	7432899.00	1931.00	41534.00
Maximum			Lm	10	9.00	1.00	90.00	26.00			3.00	7915410.00	2061.00	83983.00
Ec1	GCF_003017975.1	SRR1220810	Lm	20	0.00	0.66	80.00	27.00	2.00	2.00	4.00	7914780.00	987.00	32761.00
Ec2	GCF_000986765.1	SRR2646874	Lm	20	3.00	0.84	80.00	2.00	2.00	0.00	2.00	8144595.00	859.00	37559.00
Ec3	GCF_002554485.1	SRR1575064	Lm	20	3.00	0.86	80.00	5.00	1.00	2.00	3.00	8094474.00	781.00	39324.00
Ec4	GCF_002209105.2	SRR1562093	Lm	20	9.00	1.00	80.00	3.00	4.00	1.00	5.00	8038710.00	679.00	42180.00
Ec5	GCF_003018035.1	SRR3535390	Lm	20	3.00	0.98	80.00	9.00	1.00	5.00	6.00	8312712.00	971.00	25152.00
Ec6	GCF_003018795.1	SRR1055859	Lm	20	2.00	1.00	80.00	1.00	2.00	2.00	4.00	8152169.00	959.00	33634.00
Ec7	GCF_001890285.1	SRR1506599	Lm	20	6.00	1.00	80.00	3.00	1.00	3.00	4.00	8117964.00	919.00	33127.00
Ec8	GCF_003112185.1	SRR1217490	Lm	20	5.00	0.89	80.00	4.00	0.00	1.00	1.00	8131017.00	946.00	32540.00
Median			Lm	20	3.00	0.94	80.00	3.50	1.50	2.00	4.00	8124490.50	932.50	33380.50
Minimum			Lm	20	0.00	0.66	80.00	1.00			1.00	7914780.00	679.00	25152.00
Maximum			Lm	20	9.00	1.00	80.00	27.00			6.00	8312712.00	987.00	42180.00
Ec1	GCF_003017975.1	SRR1220810	Lm	30	0.00	0.56	70.00	29.00	1.00	3.00	4.00	7936352.00	357.00	59169.00
Ec2	GCF_000986765.1	SRR2646874	Lm	30	3.00	0.86	70.00	8.00	2.00	1.00	3.00	8170087.00	370.00	55425.00
Ec3	GCF_002554485.1	SRR1575064	Lm	30	3.00	0.89	70.00	8.00	1.00	8.00	9.00	8122494.00	382.00	52305.00
Ec4	GCF_002209105.2	SRR1562093	Lm	30	9.00	1.00	70.00	8.00	4.00	1.00	5.00	8065668.00	324.00	64967.00
Ec5	GCF_003018035.1	SRR3535390	Lm	30	3.00	0.96	70.00	10.00	1.00	3.00	4.00	8306622.00	448.00	54315.00
Ec6	GCF_003018795.1	SRR1055859	Lm	30	2.00	1.00	70.00	4.00	3.00	2.00	5.00	8163140.00	408.00	67070.00
Ec7	GCF_001890285.1	SRR1506599	Lm	30	6.00	1.00	70.00	5.00	1.00	5.00	6.00	8130963.00	366.00	73474.00
Ec8	GCF_003112185.1	SRR1217490	Lm	30	5.00	0.88	70.00	4.00	4.00	3.00	7.00	8142594.00	404.00	57993.00
Median			Lm	30	3.00	0.93	70.00	8.00	1.50	3.00	5.00	8136778.50	376.00	58581.00
Minimum			Lm	30	0.00	0.56	70.00	4.00			3.00	7936352.00	324.00	52305.00
Maximum			Lm	30	9.00	1.00	70.00	29.00			9.00	8306622.00	448.00	73474.00
Ec1	GCF_003017975.1	SRR1220810	Lm	40	0.00	0.59	60.00	39.00	3.00	12.00	15.00	7928196.00	372.00	67762.00
Ec2	GCF_000986765.1	SRR2646874	Lm	40	3.00	0.86	60.00	12.00	4.00	9.00	13.00	8162215.00	374.00	71035.00
Ec3	GCF_002554485.1	SRR1575064	Lm	40	3.00	0.82	60.00	14.00	1.00	5.00	6.00	8109999.00	386.00	80481.00
Ec4	GCF_002209105.2	SRR1562093	Lm	40	9.00	1.00	60.00	8.00	2.00	7.00	9.00	8051344.00	355.00	63391.00
Ec5	GCF_003018035.1	SRR3535390	Lm	40	3.00	0.96	60.00	15.00	5.00	7.00	12.00	8299631.00	476.00	55558.00
Ec6	GCF_003018795.1	SRR1055859	Lm	40	2.00	1.00	60.00	14.00	5.00	7.00	12.00	8155191.00	444.00	55180.00
Ec7	GCF_001890285.1	SRR1506599	Lm	40	6.00	1.00	60.00	8.00	7.00	7.00	14.00	8125271.00	382.00	72043.00
Ec8	GCF_003112185.1	SRR1217490	Lm	40	5.00	0.88	60.00	12.00	4.00	7.00	11.00	8138708.00	424.00	84519.00
Median			Lm	40	3.00	0.92	60.00	13.00	4.00	7.00	12.00	8131989.50	384.00	69398.50
Minimum			Lm	40	0.00	0.59	60.00	8.00			6.00	7928196.00	355.00	55180.00
Maximum			Lm	40	9.00	1.00	60.00	39.00			15.00	8299631.00	476.00	84519.00
Ec1	GCF_003017975.1	SRR1220810	Lm	50	0.00	0.69	50.00	51.00	7.00	20.00	27.00	7918569.00	516.00	39088.00
Ec2	GCF_000986765.1	SRR2646874	Lm	50	3.00	0.86	50.00	25.00	5.00	24.00	29.00	8151714.00	565.00	36969.00
Ec3	GCF_002554485.1	SRR1575064	Lm	50	3.00	0.92	50.00	26.00	9.00	20.00	29.00	8098680.00	552.00	38566.00
Ec4	GCF_002209105.2	SRR1562093	Lm	50	9.00	1.00	50.00	34.00	17.00	18.00	35.00	8032797.00	507.00	38700.00
Ec5	GCF_003018035.1	SRR3535390	Lm	50	3.00	1.00	50.70	135.00	10.00	30.00	40.00	8318943.00	660.00	32258.00
Ec6	GCF_003018795.1	SRR1055859	Lm	50	2.00	1.00	50.00	33.00	6.00	18.00	24.00	8132506.00	597.00	39586.00
Ec7	GCF_001890285.1	SRR1506599	Lm	50	6.00	0.97	50.00	33.00	6.00	25.00	31.00	8110225.00	570.00	32147.00
Ec8	GCF_003112185.1	SRR1217490	Lm	50	5.00	0.92	50.32	129.00	11.00	20.00	31.00	8135731.00	605.00	36413.00
Median			Lm	50	3.00	0.95	50.00	33.50	8.00	20.00	30.00	8121365.50	567.50	37767.50
Minimum			Lm	50	0.00	0.69	50.00	25.00			24.00	7918569.00	507.00	32147.00
Maximum			Lm	50	9.00	1.00	50.70	135.00			40.00	8318943.00	660.00	39586.00
Ec1	GCF_003017975.1	SRR6505947	Se	10	0.00	0.69	90.60	28.00	1.00	1.00	2.00	7306522.00	2774.00	41046.00
Ec2	GCF_000986765.1	SRR6481044	Se	10	3.00	0.77	90.57	4.00	1.00	2.00	3.00	7507826.00	2896.00	43570.00
Ec3	GCF_002554485.1	SRR7253178	Se	10	3.00	0.92	90.58	5.00	0.00	1.00	1.00	7553216.00	2841.00	43570.00
Ec4	GCF_002209105.2	SRR6984437	Se	10	8.00	1.00	90.56	6.00	2.00	2.00	4.00	7494806.00	2814.00	42425.00
Ec5	GCF_003018035.1	SRR7001455	Se	10	3.00	0.98	90.56	8.00	0.00	4.00	4.00	7905601.00	3074.00	30745.00
Ec6	GCF_003018795.1	SRR975400	Se	10	2.00	1.00	90.58	2.00	2.00	1.00	3.00	7696381.00	2992.00	34163.00
Ec7	GCF_001890285.1	SRR5725237	Se	10	6.00	1.00	90.56	2.00	0.00	0.00	0.00	7588531.00	2850.00	55223.00
Ec8	GCF_003112185.1	SRR6505965	Se	10	5.00	0.95	90.53	4.00	1.00	0.00	1.00	7728475.00	3042.00	34245.00
Median			Se	10	3.00	0.97	90.57	4.50	1.00	1.00	2.50	7570873.50	2873.00	41735.50
Minimum			Se	10	0.00	0.69	90.53	2.00			0.00	7306522.00	2774.00	30745.00
Maximum			Se	10	8.00	1.00	90.60	28.00			4.00	7905601.00	3074.00	55223.00
Ec1	GCF_003017975.1	SRR6505947	Se	20	0.00	0.59	81.20	41.00	5.00	16.00	21.00	9000689.00	2827.00	9863.00
Ec2	GCF_000986765.1	SRR6481044	Se	20	3.00	0.90	81.16	8.00	7.00	26.00	33.00	9313114.00	3082.00	7615.00
Ec3	GCF_002554485.1	SRR7253178	Se	20	3.00	0.85	81.14	7.00	7.00	14.00	21.00	9159898.00	2800.00	10693.00
Ec4	GCF_002209105.2	SRR6984437	Se	20	9.00	1.00	81.18	16.00	2.00	10.00	12.00	9215951.00	2757.00	12667.00
Ec5	GCF_003018035.1	SRR7001455	Se	20	3.00	0.99	81.11	23.00	2.00	15.00	17.00	9598328.00	2881.00	7766.00
Ec6	GCF_003018795.1	SRR975400	Se	20	2.00	1.00	81.13	10.00	4.00	12.00	16.00	9366391.00	2815.00	9301.00
Ec7	GCF_001890285.1	SRR5725237	Se	20	6.00	1.00	81.13	15.00	2.00	7.00	9.00	9259916.00	2853.00	9601.00
Ec8	GCF_003112185.1	SRR6505965	Se	20	5.00	0.96	81.16	14.00	5.00	17.00	22.00	9458430.00	2955.00	7968.00
Median			Se	20	3.00	0.98	81.15	14.50	4.50	14.50	19.00	9286515.00	2840.00	9451.00
Minimum			Se	20	0.00	0.59	81.11	7.00			9.00	9000689.00	2757.00	7615.00
Maximum			Se	20	9.00	1.00	81.20	41.00			33.00	9598328.00	3082.00	12667.00
Ec1	GCF_003017975.1	SRR6505947	Se	30	1.00	0.67	71.78	45.00	8.00	21.00	29.00	9508951.00	1698.00	11821.00
Ec2	GCF_000986765.1	SRR6481044	Se	30	3.00	0.85	71.76	21.00	7.00	22.00	29.00	9784797.00	1876.00	10658.00
Ec3	GCF_002554485.1	SRR7253178	Se	30	3.00	0.82	71.72	17.00	8.00	21.00	29.00	9624827.00	1647.00	12304.00
Ec4	GCF_002209105.2	SRR6984437	Se	30	8.00	1.00	71.74	26.00	8.00	16.00	24.00	9719237.00	1724.00	12355.00
Ec5	GCF_003018035.1	SRR7001455	Se	30	3.00	1.00	71.65	31.00	7.00	29.00	36.00	10064034.00	1733.00	12324.00
Ec6	GCF_003018795.1	SRR975400	Se	30	3.00	1.00	71.75	17.00	10.00	18.00	28.00	9832686.00	1673.00	12241.00
Ec7	GCF_001890285.1	SRR5725237	Se	30	6.00	1.00	71.74	17.00	6.00	22.00	28.00	9744535.00	1606.00	12956.00
Ec8	GCF_003112185.1	SRR6505965	Se	30	5.00	0.95	71.66	20.00	16.00	15.00	31.00	9938441.00	1803.00	11298.00
Median			Se	30	3.00	0.98	71.74	20.50	8.00	21.00	29.00	9764666.00	1711.00	12272.50
Minimum			Se	30	1.00	0.67	71.65	17.00			24.00	9508951.00	1606.00	10658.00
Maximum			Se	30	8.00	1.00	71.78	45.00			36.00	10064034.00	1876.00	12956.00
Ec1	GCF_003017975.1	SRR6505947	Se	40	1.00	0.66	62.36	70.00	7.00	18.00	25.00	9615936.00	1114.00	18280.00
Ec2	GCF_000986765.1	SRR6481044	Se	40	5.00	0.90	62.32	40.00	11.00	21.00	32.00	9895375.00	1202.00	16734.00
Ec3	GCF_002554485.1	SRR7253178	Se	40	5.00	0.91	62.33	39.00	9.00	18.00	27.00	9734232.00	1053.00	18295.00
Ec4	GCF_002209105.2	SRR6984437	Se	40	8.00	1.00	62.31	37.00	10.00	21.00	31.00	9841239.00	1067.00	18543.00
Ec5	GCF_003018035.1	SRR7001455	Se	40	4.00	0.98	62.24	56.00	10.00	14.00	24.00	10158062.00	1142.00	19158.00
Ec6	GCF_003018795.1	SRR975400	Se	40	2.00	1.00	62.35	37.00	6.00	18.00	24.00	9915753.00	1078.00	19258.00
Ec7	GCF_001890285.1	SRR5725237	Se	40	11.00	0.98	62.33	49.00	10.00	23.00	33.00	9858700.00	991.00	20649.00
Ec8	GCF_003112185.1	SRR6505965	Se	40	6.00	0.93	62.25	38.00	11.00	17.00	28.00	10030183.00	1152.00	18986.00
Median			Se	40	5.00	0.96	62.33	39.50	10.00	18.00	27.50	9877037.50	1096.00	18764.50
Minimum			Se	40	1.00	0.66	62.24	37.00			24.00	9615936.00	991.00	16734.00
Maximum			Se	40	11.00	1.00	62.36	70.00			33.00	10158062.00	1202.00	20649.00
Ec1	GCF_003017975.1	SRR6505947	Se	50	4.00	0.63	52.96	100.00	13.00	28.00	41.00	9610220.00	982.00	20530.00
Ec2	GCF_000986765.1	SRR6481044	Se	50	17.00	0.91	52.95	83.00	10.00	18.00	28.00	9898746.00	1007.00	20052.00
Ec3	GCF_002554485.1	SRR7253178	Se	50	18.00	0.88	52.91	86.00	15.00	25.00	40.00	9731946.00	1007.00	20816.00
Ec4	GCF_002209105.2	SRR6984437	Se	50	16.00	1.00	52.87	101.00	7.00	26.00	33.00	9852947.00	1025.00	19357.00
Ec5	GCF_003018035.1	SRR7001455	Se	50	5.00	0.98	52.80	88.00	11.00	17.00	28.00	10152180.00	1121.00	20585.00
Ec6	GCF_003018795.1	SRR975400	Se	50	8.00	1.00	52.96	99.00	13.00	24.00	37.00	9899151.00	1070.00	19851.00
Ec7	GCF_001890285.1	SRR5725237	Se	50	17.00	0.99	52.85	90.00	8.00	19.00	27.00	9853608.00	978.00	21773.00
Ec8	GCF_003112185.1	SRR6505965	Se	50	10.00	0.99	52.76	97.00	11.00	25.00	36.00	10031767.00	1103.00	19907.00
Median			Se	50	13.00	0.99	52.89	93.50	11.00	24.50	34.50	9876177.00	1016.00	20291.00
Minimum			Se	50	4.00	0.63	52.76	83.00			27.00	9610220.00	978.00	19357.00
Maximum			Se	50	18.00	1.00	52.96	101.00			41.00	10152180.00	1121.00	21773.00

Note: Lm=*Listeria monocytogenes*, Se=*Salmonella enterica*, Ec=*Escherichia coli*

Table S3: P-values for results of clustering analyses

		Percent Contamination	Type of Contamination				
			0.05	0.5	5	Interspecies 1	Interspecies 2
SNPs	Lm	10	0.500000	0.443226	0.284153	0.387883	0.387883
		20	0.500000	0.443226	0.284153	0.387883	0.443226
		30	0.443226	0.446356	0.197241	0.387883	0.443226
		40	0.156440	0.018925	0.000008	0.443226	0.338259
		50	0.061296	0.000008	0.000000	0.387883	0.338259
	Se	10	0.500000	0.500000	0.500000	0.500000	0.500000
		20	0.451314	0.453328	0.359447	0.500000	0.500000
		30	0.235723	0.234817	0.044249	0.500000	0.452945
		40	0.092559	0.002339	0.000030	0.500000	0.231879
		50	0.001175	0.000019	0.000004	0.500000	0.000314
	Ec	10	0.500000	0.462726	0.500000	0.500000	0.462726
		20	0.500000	0.461102	0.500000	0.500000	0.500000
		30	0.395715	0.239395	0.103638	0.500000	0.460774
		40	0.274397	0.000090	0.001924	0.500000	0.186085
		50	0.008200	0.000009	0.001692	0.500000	0.002727
Bootstrap Support	Lm	10	0.432838	0.371454	0.283775	0.395872	0.339222
		20	0.452578	0.350040	0.304115	0.446916	0.486978
		30	0.295464	0.288857	0.388814	0.429683	0.422231
		40	0.131121	0.141059	0.045782	0.430531	0.421211
		50	0.070907	0.030222	0.046646	0.423953	0.477883
	Se	10	0.456498	0.463204	0.448462	0.483713	0.466525
		20	0.477864	0.458853	0.484177	0.485031	0.492409
		30	0.284075	0.471262	0.471262	0.470806	0.484366
		40	0.270890	0.474857	0.472353	0.434368	0.442254
		50	0.065000	0.143873	0.434106	0.485425	0.451449
	Ec	10	0.460309	0.426898	0.470219	0.379607	0.484056
		20	0.406838	0.469319	0.412557	0.492083	0.449290
		30	0.219912	0.122859	0.290507	0.267781	0.345929
		40	0.085139	0.119429	0.243748	0.262091	0.430058
		50	0.021805	0.011249	0.087674	0.414148	0.405693
Alleles	Lm	10	0.070847	0.092330	0.000000	0.297395	0.113409
		20	0.216444	0.000307	0.000000	0.294648	0.035485
		30	0.055101	0.000008	0.000004	0.020441	0.069189
		40	0.001683	0.000004	0.000000	0.000746	0.000001
		50	0.000568	0.000008	0.000040	0.000150	0.000000
	Se	10	0.308800	0.047596	0.000382	0.374031	0.078732
		20	0.250174	0.000000	0.000000	0.184584	0.000058
		30	0.044861	0.000002	0.000001	0.141440	0.000000
		40	0.011770	0.000000	0.000000	0.000000	0.000000
		50	0.010191	0.000000	0.000000	0.000000	0.000000
	Ec	10	0.465935	0.306187	0.055775	0.477030	0.355180
		20	0.116039	0.002082	0.005231	0.398098	0.002176
		30	0.353863	0.000045	0.000882	0.324842	0.001186
		40	0.065264	0.000020	0.000326	0.061791	0.000003
		50	0.006246	0.000008	0.000185	0.009112	0.000000
Missing & Partial	Lm	10	0.423073	0.212986	0.150462	0.500000	0.343933
		20	0.043323	0.000158	0.000059	0.050641	0.019556
		30	0.000947	0.000055	0.000000	0.000900	0.027594
		40	0.000457	0.000036	0.000001	0.000019	0.000035
		50	0.000034	0.000020	0.000001	0.000000	0.000000
	Se	10	0.500000	0.040160	0.043719	0.000001	0.330806
		20	0.039707	0.000002	0.000009	0.000000	0.000018
		30	0.009360	0.000001	0.000000	0.000000	0.000000
		40	0.009153	0.000000	0.000000	0.000061	0.000000
		50	0.022371	0.000000	0.000000	0.000000	0.000000
	Ec	10	0.240123	0.346362	0.016840	0.418415	0.142978
		20	0.500000	0.000423	0.001174	0.016824	0.000131
		30	0.040509	0.000128	0.000703	0.001921	0.000000
		40	0.040030	0.000064	0.001640	0.000025	0.000000
		50	0.057000	0.000053	0.001353	0.000000	0.000000

Note: Lm=*Listeria monocytogenes* , Se=*Salmonella enterica* , Ec=*Escherichia coli* . Values less than or equal to 0.05 are highlighted.

Table S5: Percent of contamination detected in fastq data from NCBI.

Taxon	Percent Contamination	Percent Between-species	Percent Within-species	Percent SNP confounding	Percent MLST confounding
**Lm**	**8.92**	**1.23**	**7.69**	**1.48**	**2.26**
**Se**	**6.38**	**0.29**	**6.09**	**2.22**	**5.06**
**Ec**	**5.47**	**0.15**	**5.33**	**0.87**	**1.26**

Note: We analyzed 10,000 randomly selected fastq datasets from NCBI for *L. monocytogenes, S. enterica,* and *E. coli* and used ConFindr to estimate levels of contamination.

Table S6: NCBI accession numbers for data generated during this study.

Strain Name	Organism	BioProject	NCBI BioSample	SRA Run
Ec_0.5_40_Ec4	Escherichia coli	PRJNA561589	SAMN12686891	SRR10063038
Ec_0.5_40_Ec3	Escherichia coli	PRJNA561589	SAMN12686894	SRR10063039
Ec_0.5_20_Ec1	Escherichia coli	PRJNA561589	SAMN12686888	SRR10063040
Ec_0.05_40_Ec5	Escherichia coli	PRJNA561589	SAMN12686931	SRR10063041
Ec_0.5_20_Ec2	Escherichia coli	PRJNA561589	SAMN12686929	SRR10063042
Ec_0.5_10_Ec7	Escherichia coli	PRJNA561589	SAMN12686923	SRR10063043
Ec_0.5_30_Ec7	Escherichia coli	PRJNA561589	SAMN12686927	SRR10063044
Ec_0.05_40_Ec4	Escherichia coli	PRJNA561589	SAMN12686924	SRR10063045
Ec_0.5_10_Ec8	Escherichia coli	PRJNA561589	SAMN12686944	SRR10063058
Ec_0.5_30_Ec6	Escherichia coli	PRJNA561589	SAMN12686945	SRR10063059
Ec_0.05_40_Ec2	Escherichia coli	PRJNA561589	SAMN12686942	SRR10063060
Ec_0.5_10_Ec5	Escherichia coli	PRJNA561589	SAMN12686943	SRR10063061
Ec_0.05_30_Ec8	Escherichia coli	PRJNA561589	SAMN12686952	SRR10063068
Ec_0.5_10_Ec6	Escherichia coli	PRJNA561589	SAMN12686954	SRR10063069
Ec_0.05_40_Ec3	Escherichia coli	PRJNA561589	SAMN12686953	SRR10063070
Ec_0.5_40_Ec2	Escherichia coli	PRJNA561589	SAMN12686962	SRR10063077
Ec_0.05_20_Ec3	Escherichia coli	PRJNA561589	SAMN12686963	SRR10063078
Ec_0.5_20_Ec5	Escherichia coli	PRJNA561589	SAMN12686961	SRR10063079
Ec_0.05_40_Ec1	Escherichia coli	PRJNA561589	SAMN12686964	SRR10063084
Ec_0.5_40_Ec6	Escherichia coli	PRJNA561589	SAMN12686976	SRR10063091
Ec_0.5_20_Ec4	Escherichia coli	PRJNA561589	SAMN12686975	SRR10063092
Ec_0.05_30_Ec4	Escherichia coli	PRJNA561589	SAMN12686977	SRR10063094
Ec_0.05_30_Ec3	Escherichia coli	PRJNA561589	SAMN12686983	SRR10063097
Ec_0.05_10_Ec2	Escherichia coli	PRJNA561589	SAMN12686984	SRR10063098
Ec_0.05_10_Ec3	Escherichia coli	PRJNA561589	SAMN12686982	SRR10063099
Ec_0.5_40_Ec1	Escherichia coli	PRJNA561589	SAMN12686981	SRR10063100
Ec_0.5_40_Ec7	Escherichia coli	PRJNA561589	SAMN12687002	SRR10063106
Ec_0.05_10_Ec1	Escherichia coli	PRJNA561589	SAMN12687005	SRR10063107
Ec_0.05_30_Ec6	Escherichia coli	PRJNA561589	SAMN12687004	SRR10063110
Ec_0.05_30_Ec7	Escherichia coli	PRJNA561589	SAMN12687003	SRR10063112
Ec_0.05_20_Ec8	Escherichia coli	PRJNA561589	SAMN12687015	SRR10063117
Ec_0.5_40_Ec8	Escherichia coli	PRJNA561589	SAMN12687016	SRR10063118
Ec_0.05_20_Ec6	Escherichia coli	PRJNA561589	SAMN12687013	SRR10063119
Ec_0.05_30_Ec1	Escherichia coli	PRJNA561589	SAMN12687014	SRR10063120
Ec_0.05_20_Ec7	Escherichia coli	PRJNA561589	SAMN12687034	SRR10063217
Ec_0.5_30_Ec5	Escherichia coli	PRJNA561589	SAMN12687033	SRR10063218
Ec_0.05_20_Ec5	Escherichia coli	PRJNA561589	SAMN12687032	SRR10063219
Ec_0.5_30_Ec4	Escherichia coli	PRJNA561589	SAMN12687038	SRR10063225
Ec_0.05_50_Ec7	Escherichia coli	PRJNA561589	SAMN12687040	SRR10063226
Ec_0.5_30_Ec3	Escherichia coli	PRJNA561589	SAMN12687041	SRR10063227
Ec_0.5_40_Ec5	Escherichia coli	PRJNA561589	SAMN12687039	SRR10063228
Ec_0.5_30_Ec2	Escherichia coli	PRJNA561589	SAMN12687042	SRR10063229
Ec_0.05_50_Ec5	Escherichia coli	PRJNA561589	SAMN12687043	SRR10063230
Ec_0.05_30_Ec2	Escherichia coli	PRJNA561589	SAMN12687044	SRR10063231
Ec_0.5_50_Ec6	Escherichia coli	PRJNA561589	SAMN12687050	SRR10063235
Ec_0.05_50_Ec3	Escherichia coli	PRJNA561589	SAMN12687049	SRR10063236
Ec_0.05_50_Ec4	Escherichia coli	PRJNA561589	SAMN12687051	SRR10063237
Ec_0.5_50_Ec5	Escherichia coli	PRJNA561589	SAMN12687048	SRR10063238
Ec_0.05_50_Ec6	Escherichia coli	PRJNA561589	SAMN12687052	SRR10063239
Ec_0.5_50_Ec4	Escherichia coli	PRJNA561589	SAMN12687059	SRR10063249
Ec_0.5_20_Ec7	Escherichia coli	PRJNA561589	SAMN12687068	SRR10063250
Ec_0.05_30_Ec5	Escherichia coli	PRJNA561589	SAMN12687069	SRR10063251
Ec_0.05_50_Ec2	Escherichia coli	PRJNA561589	SAMN12687060	SRR10063252
Ec_0.05_40_Ec7	Escherichia coli	PRJNA561589	SAMN12687092	SRR10063344
Ec_0.5_50_Ec3	Escherichia coli	PRJNA561589	SAMN12687089	SRR10063345
Ec_0.5_20_Ec6	Escherichia coli	PRJNA561589	SAMN12687097	SRR10063346
Ec_0.5_20_Ec3	Escherichia coli	PRJNA561589	SAMN12687101	SRR10063347
Ec_0.5_50_Ec2	Escherichia coli	PRJNA561589	SAMN12687234	SRR10063369
Ec_0.05_20_Ec4	Escherichia coli	PRJNA561589	SAMN12687236	SRR10063370
Ec_0.05_50_Ec1	Escherichia coli	PRJNA561589	SAMN12687233	SRR10063371
Ec_0.05_40_Ec6	Escherichia coli	PRJNA561589	SAMN12687235	SRR10063372
Ec_0.5_20_Ec8	Escherichia coli	PRJNA561589	SAMN12687240	SRR10063373
Ec_0.5_30_Ec1	Escherichia coli	PRJNA561589	SAMN12687239	SRR10063374
Ec_0.5_50_Ec1	Escherichia coli	PRJNA561589	SAMN12687238	SRR10063375
Ec_0.5_30_Ec8	Escherichia coli	PRJNA561589	SAMN12687241	SRR10063376
Ec_0.05_40_Ec8	Escherichia coli	PRJNA561589	SAMN12687485	SRR10063379
Ec_0.05_10_Ec8	Escherichia coli	PRJNA561589	SAMN12687481	SRR10063380
Ec_0.05_20_Ec1	Escherichia coli	PRJNA561589	SAMN12687483	SRR10063381
Ec_0.5_10_Ec2	Escherichia coli	PRJNA561589	SAMN12687484	SRR10063382
Ec_0.05_10_Ec7	Escherichia coli	PRJNA561589	SAMN12687482	SRR10063383
Ec_0.5_50_Ec8	Escherichia coli	PRJNA561589	SAMN12687495	SRR10063391
Ec_0.05_10_Ec5	Escherichia coli	PRJNA561589	SAMN12687496	SRR10063392
Ec_0.05_10_Ec6	Escherichia coli	PRJNA561589	SAMN12687497	SRR10063393
Ec_Se_20_Ec1	Escherichia coli	PRJNA561589	SAMN12687516	SRR10063411
Ec_Lm_40_Ec5	Escherichia coli	PRJNA561589	SAMN12687517	SRR10063412
Ec_0.5_50_Ec7	Escherichia coli	PRJNA561589	SAMN12687520	SRR10063413
Ec_0.05_10_Ec4	Escherichia coli	PRJNA561589	SAMN12687521	SRR10063414
Lm_0.05_30_Lm6	Listeria	PRJNA561589	SAMN12687518	SRR10063415
Lm_0.05_30_Lm5	Listeria	PRJNA561589	SAMN12687538	SRR10063418
Ec_5_10_Ec8	Escherichia coli	PRJNA561589	SAMN12687537	SRR10063419
Ec_0.05_20_Ec2	Escherichia coli	PRJNA561589	SAMN12687534	SRR10063420
Lm_0.05_30_Lm4	Listeria	PRJNA561589	SAMN12687535	SRR10063421
Ec_5_10_Ec7	Escherichia coli	PRJNA561589	SAMN12687536	SRR10063422
Ec_Se_10_Ec8	Escherichia coli	PRJNA561589	SAMN12687555	SRR10063423
Ec_Lm_50_Ec1	Escherichia coli	PRJNA561589	SAMN12687554	SRR10063424
Ec_0.5_10_Ec4	Escherichia coli	PRJNA561589	SAMN12687553	SRR10063425
Ec_0.5_10_Ec1	Escherichia coli	PRJNA561589	SAMN12687552	SRR10063426
Ec_0.5_10_Ec3	Escherichia coli	PRJNA561589	SAMN12687558	SRR10063427
Lm_0.05_20_Lm2	Listeria	PRJNA561589	SAMN12687559	SRR10063429
Ec_5_10_Ec6	Escherichia coli	PRJNA561589	SAMN12687557	SRR10063430
Ec_Self_40_Ec7	Escherichia coli	PRJNA561589	SAMN12687556	SRR10063432
Ec_Self_40_Ec6	Escherichia coli	PRJNA561589	SAMN12687570	SRR10063656
Ec_0.05_50_Ec8	Escherichia coli	PRJNA561589	SAMN12687572	SRR10063657
Lm_0.05_30_Lm3	Listeria	PRJNA561589	SAMN12687571	SRR10063658
Ec_5_10_Ec5	Escherichia coli	PRJNA561589	SAMN12687573	SRR10063676
Ec_Se_10_Ec7	Escherichia coli	PRJNA561589	SAMN12687591	SRR10063681
Ec_Lm_30_Ec3	Escherichia coli	PRJNA561589	SAMN12687588	SRR10063682
Ec_Lm_20_Ec7	Escherichia coli	PRJNA561589	SAMN12687589	SRR10063683
Ec_Lm_40_Ec8	Escherichia coli	PRJNA561589	SAMN12687590	SRR10063684
Ec_Lm_30_Ec2	Escherichia coli	PRJNA561589	SAMN12687603	SRR10063685
Ec_5_10_Ec4	Escherichia coli	PRJNA561589	SAMN12687604	SRR10063686
Lm_0.05_30_Lm2	Listeria	PRJNA561589	SAMN12687602	SRR10063687
Ec_Self_50_Ec2	Escherichia coli	PRJNA561589	SAMN12687601	SRR10063688
Ec_Se_10_Ec6	Escherichia coli	PRJNA561589	SAMN12687606	SRR10063689
Ec_Lm_30_Ec1	Escherichia coli	PRJNA561589	SAMN12687607	SRR10063690
Lm_0.05_20_Lm1	Listeria	PRJNA561589	SAMN12687608	SRR10063691
Ec_Self_50_Ec1	Escherichia coli	PRJNA561589	SAMN12687605	SRR10063692
Ec_Se_50_Ec8	Escherichia coli	PRJNA561589	SAMN12687610	SRR10063693
Ec_Self_40_Ec8	Escherichia coli	PRJNA561589	SAMN12687649	SRR10063694
Ec_Lm_20_Ec8	Escherichia coli	PRJNA561589	SAMN12687612	SRR10063695
Ec_Self_0_Ec1	Escherichia coli	PRJNA561589	SAMN12687615	SRR10063696
Ec_Lm_50_Ec2	Escherichia coli	PRJNA561589	SAMN12687653	SRR10063697
Ec_Self_50_Ec4	Escherichia coli	PRJNA561589	SAMN12687652	SRR10063698
Ec_5_20_Ec1	Escherichia coli	PRJNA561589	SAMN12687655	SRR10063699
Ec_5_20_Ec5	Escherichia coli	PRJNA561589	SAMN12687651	SRR10063700
Lm_0.05_20_Lm4	Listeria	PRJNA561589	SAMN12687654	SRR10063701
Ec_Lm_30_Ec4	Escherichia coli	PRJNA561589	SAMN12687656	SRR10063702
Ec_5_20_Ec4	Escherichia coli	PRJNA561589	SAMN12687658	SRR10063703
Ec_Self_50_Ec3	Escherichia coli	PRJNA561589	SAMN12687659	SRR10063704
Ec_Lm_30_Ec5	Escherichia coli	PRJNA561589	SAMN12687660	SRR10063705
Lm_0.05_20_Lm6	Listeria	PRJNA561589	SAMN12687657	SRR10063706
Ec_5_20_Ec2	Escherichia coli	PRJNA561589	SAMN12687662	SRR10063747
Ec_Lm_50_Ec3	Escherichia coli	PRJNA561589	SAMN12687663	SRR10063748
Lm_0.05_20_Lm5	Listeria	PRJNA561589	SAMN12687661	SRR10063749
Ec_5_20_Ec3	Escherichia coli	PRJNA561589	SAMN12687664	SRR10063750
Ec_Lm_30_Ec8	Escherichia coli	PRJNA561589	SAMN12687668	SRR10063751
Ec_Lm_30_Ec7	Escherichia coli	PRJNA561589	SAMN12687667	SRR10063752
Ec_Lm_30_Ec6	Escherichia coli	PRJNA561589	SAMN12687665	SRR10063753
Ec_Self_50_Ec7	Escherichia coli	PRJNA561589	SAMN12687666	SRR10063754
Lm_0.05_20_Lm7	Listeria	PRJNA561589	SAMN12687669	SRR10063755
Ec_Self_50_Ec5	Escherichia coli	PRJNA561589	SAMN12687670	SRR10063756
Ec_5_50_Ec5	Escherichia coli	PRJNA561589	SAMN12687671	SRR10063757
Ec_Self_50_Ec6	Escherichia coli	PRJNA561589	SAMN12687672	SRR10063758
Ec_5_20_Ec7	Escherichia coli	PRJNA561589	SAMN12687676	SRR10063759
Ec_Lm_10_Ec4	Escherichia coli	PRJNA561589	SAMN12687674	SRR10063760
Ec_5_30_Ec4	Escherichia coli	PRJNA561589	SAMN12687675	SRR10063761
Ec_Lm_10_Ec5	Escherichia coli	PRJNA561589	SAMN12687673	SRR10063762
Lm_0.05_20_Lm8	Listeria	PRJNA561589	SAMN12687680	SRR10063763
Lm_0.05_20_Lm3	Listeria	PRJNA561589	SAMN12687681	SRR10063764
Lm_0.05_40_Lm2	Listeria	PRJNA561589	SAMN12687678	SRR10063765
Ec_5_20_Ec8	Escherichia coli	PRJNA561589	SAMN12687677	SRR10063766
Lm_0.05_40_Lm1	Listeria	PRJNA561589	SAMN12687682	SRR10063767
Ec_Lm_10_Ec3	Escherichia coli	PRJNA561589	SAMN12687679	SRR10063768
Ec_5_30_Ec3	Escherichia coli	PRJNA561589	SAMN12687685	SRR10063769
Ec_Lm_10_Ec1	Escherichia coli	PRJNA561589	SAMN12687683	SRR10063770
Ec_Lm_50_Ec5	Escherichia coli	PRJNA561589	SAMN12687684	SRR10063771
Ec_5_50_Ec4	Escherichia coli	PRJNA561589	SAMN12687686	SRR10063772
Ec_Lm_10_Ec2	Escherichia coli	PRJNA561589	SAMN12687689	SRR10063773
Ec_5_50_Ec8	Escherichia coli	PRJNA561589	SAMN12687687	SRR10063774
Ec_5_50_Ec7	Escherichia coli	PRJNA561589	SAMN12687688	SRR10063775
Ec_Lm_10_Ec8	Escherichia coli	PRJNA561589	SAMN12687692	SRR10063776
Lm_0.05_30_Lm8	Listeria	PRJNA561589	SAMN12687691	SRR10063777
Ec_5_50_Ec3	Escherichia coli	PRJNA561589	SAMN12687690	SRR10063778
Ec_5_50_Ec6	Escherichia coli	PRJNA561589	SAMN12687693	SRR10063779
Ec_Lm_20_Ec6	Escherichia coli	PRJNA561589	SAMN12687695	SRR10063780
Ec_5_30_Ec2	Escherichia coli	PRJNA561589	SAMN12687694	SRR10063781
Ec_Se_50_Ec1	Escherichia coli	PRJNA561589	SAMN12687697	SRR10063782
Lm_0.05_30_Lm7	Listeria	PRJNA561589	SAMN12687696	SRR10063783
Ec_5_30_Ec1	Escherichia coli	PRJNA561589	SAMN12687699	SRR10063784
Ec_Lm_10_Ec7	Escherichia coli	PRJNA561589	SAMN12687701	SRR10063785
Ec_Lm_50_Ec8	Escherichia coli	PRJNA561589	SAMN12687698	SRR10063786
Ec_Se_10_Ec1	Escherichia coli	PRJNA561589	SAMN12687700	SRR10063787
Ec_Lm_50_Ec7	Escherichia coli	PRJNA561589	SAMN12687703	SRR10063835
Ec_Se_50_Ec2	Escherichia coli	PRJNA561589	SAMN12687702	SRR10063836
Ec_Se_50_Ec3	Escherichia coli	PRJNA561589	SAMN12687704	SRR10063837
Ec_Lm_20_Ec3	Escherichia coli	PRJNA561589	SAMN12687706	SRR10063838
Ec_Self_40_Ec4	Escherichia coli	PRJNA561589	SAMN12687708	SRR10063839
Ec_Lm_20_Ec4	Escherichia coli	PRJNA561589	SAMN12687705	SRR10063840
Ec_Lm_20_Ec5	Escherichia coli	PRJNA561589	SAMN12687707	SRR10063841
Ec_5_20_Ec6	Escherichia coli	PRJNA561589	SAMN12687710	SRR10063842
Ec_Lm_50_Ec6	Escherichia coli	PRJNA561589	SAMN12687709	SRR10063843
Ec_Lm_20_Ec2	Escherichia coli	PRJNA561589	SAMN12687711	SRR10063844
Ec_Lm_20_Ec1	Escherichia coli	PRJNA561589	SAMN12687712	SRR10063845
Ec_Lm_10_Ec6	Escherichia coli	PRJNA561589	SAMN12687715	SRR10063846
Lm_0.05_30_Lm1	Listeria	PRJNA561589	SAMN12687714	SRR10063847
Ec_Self_40_Ec5	Escherichia coli	PRJNA561589	SAMN12687713	SRR10063848
Ec_5_40_Ec7	Escherichia coli	PRJNA561589	SAMN12687716	SRR10063849
Ec_5_50_Ec2	Escherichia coli	PRJNA561589	SAMN12687718	SRR10063850
Ec_5_50_Ec1	Escherichia coli	PRJNA561589	SAMN12687719	SRR10063851
Ec_Lm_50_Ec4	Escherichia coli	PRJNA561589	SAMN12687717	SRR10063852
Ec_5_40_Ec8	Escherichia coli	PRJNA561589	SAMN12687723	SRR10063853
Ec_5_40_Ec5	Escherichia coli	PRJNA561589	SAMN12687722	SRR10063854
Ec_5_40_Ec6	Escherichia coli	PRJNA561589	SAMN12687721	SRR10063855
Ec_Se_10_Ec2	Escherichia coli	PRJNA561589	SAMN12687720	SRR10063856
Ec_5_40_Ec4	Escherichia coli	PRJNA561589	SAMN12687726	SRR10063857
Ec_Lm_40_Ec2	Escherichia coli	PRJNA561589	SAMN12687724	SRR10063858
Ec_Se_50_Ec4	Escherichia coli	PRJNA561589	SAMN12687725	SRR10063859
Lm_0.05_10_Lm1	Listeria	PRJNA561589	SAMN12687729	SRR10063860
Lm_0.05_10_Lm2	Listeria	PRJNA561589	SAMN12687731	SRR10063861
Ec_Se_10_Ec5	Escherichia coli	PRJNA561589	SAMN12687728	SRR10063862
Ec_Lm_40_Ec1	Escherichia coli	PRJNA561589	SAMN12687727	SRR10063863
Ec_Self_50_Ec8	Escherichia coli	PRJNA561589	SAMN12687730	SRR10063864
Ec_Se_50_Ec6	Escherichia coli	PRJNA561589	SAMN12687733	SRR10063865
Ec_Se_10_Ec3	Escherichia coli	PRJNA561589	SAMN12687732	SRR10063866
Ec_Se_50_Ec7	Escherichia coli	PRJNA561589	SAMN12687734	SRR10063867
Ec_Se_10_Ec4	Escherichia coli	PRJNA561589	SAMN12687735	SRR10063868
Lm_0.05_10_Lm4	Listeria	PRJNA561589	SAMN12687738	SRR10063869
Lm_0.05_10_Lm3	Listeria	PRJNA561589	SAMN12687737	SRR10063870
Lm_0.05_10_Lm5	Listeria	PRJNA561589	SAMN12687740	SRR10063902
Ec_Lm_40_Ec4	Escherichia coli	PRJNA561589	SAMN12687739	SRR10063903
Ec_Se_50_Ec5	Escherichia coli	PRJNA561589	SAMN12687743	SRR10063904
Ec_Lm_40_Ec3	Escherichia coli	PRJNA561589	SAMN12687741	SRR10063905
Ec_5_10_Ec3	Escherichia coli	PRJNA561589	SAMN12687742	SRR10063906
Lm_0.05_10_Lm7	Listeria	PRJNA561589	SAMN12687744	SRR10063907
Ec_5_10_Ec2	Escherichia coli	PRJNA561589	SAMN12687747	SRR10063908
Ec_Lm_40_Ec7	Escherichia coli	PRJNA561589	SAMN12687745	SRR10063909
Lm_0.05_10_Lm8	Listeria	PRJNA561589	SAMN12687746	SRR10063910
Ec_Lm_40_Ec6	Escherichia coli	PRJNA561589	SAMN12687748	SRR10063911
Lm_0.05_10_Lm6	Listeria	PRJNA561589	SAMN12687749	SRR10063912
Ec_5_10_Ec1	Escherichia coli	PRJNA561589	SAMN12687750	SRR10063913
Ec_Self_40_Ec3	Escherichia coli	PRJNA561589	SAMN12717914	SRR10088477
Ec_Self_40_Ec1	Escherichia coli	PRJNA561589	SAMN12717912	SRR10088483
Lm_5_40_Lm6	Listeria	PRJNA561589	SAMN12717913	SRR10088484
Ec_Self_40_Ec2	Escherichia coli	PRJNA561589	SAMN12717922	SRR10088540
Lm_5_40_Lm7	Listeria	PRJNA561589	SAMN12717972	SRR10088541
Lm_Ec_10_Lm2	Listeria	PRJNA561589	SAMN12717973	SRR10088542
Se_Self_40_Se1	Salmonella enterica	PRJNA561589	SAMN12717993	SRR10088555
Lm_5_50_Lm5	Listeria	PRJNA561589	SAMN12717989	SRR10088556
Se_Self_30_Se8	Salmonella enterica	PRJNA561589	SAMN12717992	SRR10088557
Se_Self_30_Se7	Salmonella enterica	PRJNA561589	SAMN12717991	SRR10088561
Lm_5_50_Lm6	Listeria	PRJNA561589	SAMN12717994	SRR10088563
Lm_5_50_Lm4	Listeria	PRJNA561589	SAMN12717998	SRR10088570
Se_Self_30_Se6	Salmonella enterica	PRJNA561589	SAMN12717996	SRR10088572
Lm_Ec_10_Lm1	Listeria	PRJNA561589	SAMN12717997	SRR10088573
Se_Self_40_Se4	Salmonella enterica	PRJNA561589	SAMN12717995	SRR10088574
Se_Self_30_Se5	Salmonella enterica	PRJNA561589	SAMN12718022	SRR10088590
Se_Self_30_Se4	Salmonella enterica	PRJNA561589	SAMN12718018	SRR10088591
Se_Self_40_Se3	Salmonella enterica	PRJNA561589	SAMN12718020	SRR10088592
Lm_5_50_Lm3	Listeria	PRJNA561589	SAMN12718019	SRR10088593
Lm_5_50_Lm8	Listeria	PRJNA561589	SAMN12718021	SRR10088594
Lm_5_50_Lm7	Listeria	PRJNA561589	SAMN12718017	SRR10088595
Se_Self_40_Se2	Salmonella enterica	PRJNA561589	SAMN12718033	SRR10088655
Ec_Self_30_Ec8	Escherichia coli	PRJNA561589	SAMN12718030	SRR10088657
Lm_5_50_Lm2	Listeria	PRJNA561589	SAMN12718032	SRR10088659
Ec_Self_30_Ec7	Escherichia coli	PRJNA561589	SAMN12718031	SRR10088660
Ec_Self_30_Ec6	Escherichia coli	PRJNA561589	SAMN12718051	SRR10088667
Ec_Self_10_Ec1	Escherichia coli	PRJNA561589	SAMN12718046	SRR10088668
Lm_5_20_Lm7	Listeria	PRJNA561589	SAMN12718049	SRR10088669
Ec_Se_40_Ec6	Escherichia coli	PRJNA561589	SAMN12718044	SRR10088670
Lm_5_20_Lm6	Listeria	PRJNA561589	SAMN12718041	SRR10088671
Lm_5_20_Lm5	Listeria	PRJNA561589	SAMN12718075	SRR10088676
Ec_Self_20_Ec4	Escherichia coli	PRJNA561589	SAMN12718078	SRR10088677
Ec_Se_40_Ec5	Escherichia coli	PRJNA561589	SAMN12718076	SRR10088678
Ec_Se_30_Ec3	Escherichia coli	PRJNA561589	SAMN12718077	SRR10088679
Ec_Self_20_Ec3	Escherichia coli	PRJNA561589	SAMN12718087	SRR10088685
Ec_Se_40_Ec4	Escherichia coli	PRJNA561589	SAMN12718086	SRR10088686
Ec_Self_0_Ec8	Escherichia coli	PRJNA561589	SAMN12718088	SRR10088687
Ec_Self_20_Ec2	Escherichia coli	PRJNA561589	SAMN12718084	SRR10088688
Lm_5_20_Lm4	Listeria	PRJNA561589	SAMN12718085	SRR10088689
Ec_Se_40_Ec8	Escherichia coli	PRJNA561589	SAMN12718097	SRR10088697
Ec_Self_10_Ec4	Escherichia coli	PRJNA561589	SAMN12718098	SRR10088698
Ec_Se_30_Ec2	Escherichia coli	PRJNA561589	SAMN12718099	SRR10088699
Ec_Self_20_Ec6	Escherichia coli	PRJNA561589	SAMN12718096	SRR10088700
Lm_5_40_Lm8	Listeria	PRJNA561589	SAMN12718107	SRR10088708
Ec_Self_20_Ec5	Escherichia coli	PRJNA561589	SAMN12718108	SRR10088709
Lm_5_20_Lm3	Listeria	PRJNA561589	SAMN12718110	SRR10088710
Ec_Se_40_Ec7	Escherichia coli	PRJNA561589	SAMN12718109	SRR10088712
Ec_Self_10_Ec3	Escherichia coli	PRJNA561589	SAMN12718117	SRR10088713
Lm_5_30_Lm4	Listeria	PRJNA561589	SAMN12718114	SRR10088714
Ec_Self_30_Ec3	Escherichia coli	PRJNA561589	SAMN12718113	SRR10088715
Lm_5_50_Lm1	Listeria	PRJNA561589	SAMN12718115	SRR10088716
Ec_Self_30_Ec5	Escherichia coli	PRJNA561589	SAMN12718116	SRR10088717
Lm_5_30_Lm3	Listeria	PRJNA561589	SAMN12718123	SRR10088720
Ec_Self_30_Ec2	Escherichia coli	PRJNA561589	SAMN12718120	SRR10088721
Ec_Self_30_Ec1	Escherichia coli	PRJNA561589	SAMN12718121	SRR10088722
Ec_Self_30_Ec4	Escherichia coli	PRJNA561589	SAMN12718122	SRR10088723
Ec_Se_30_Ec6	Escherichia coli	PRJNA561589	SAMN12718213	SRR10088730
Ec_Self_20_Ec7	Escherichia coli	PRJNA561589	SAMN12718215	SRR10088731
Ec_Self_20_Ec8	Escherichia coli	PRJNA561589	SAMN12718214	SRR10088732
Lm_5_20_Lm8	Listeria	PRJNA561589	SAMN12718211	SRR10088733
Ec_5_40_Ec2	Escherichia coli	PRJNA561589	SAMN12718212	SRR10088734
Lm_5_40_Lm1	Listeria	PRJNA561589	SAMN12718219	SRR10088735
Ec_Self_0_Ec7	Escherichia coli	PRJNA561589	SAMN12718218	SRR10088736
Ec_Self_10_Ec6	Escherichia coli	PRJNA561589	SAMN12718217	SRR10088737
Lm_0.5_30_Lm5	Listeria	PRJNA561589	SAMN12718220	SRR10088738
Lm_5_10_Lm1	Listeria	PRJNA561589	SAMN12718216	SRR10088739
Lm_5_30_Lm8	Listeria	PRJNA561589	SAMN12718224	SRR10088740
Lm_5_20_Lm2	Listeria	PRJNA561589	SAMN12718225	SRR10088741
Lm_5_30_Lm7	Listeria	PRJNA561589	SAMN12718221	SRR10088742
Ec_Se_30_Ec1	Escherichia coli	PRJNA561589	SAMN12718223	SRR10088743
Ec_Se_30_Ec8	Escherichia coli	PRJNA561589	SAMN12718222	SRR10088744
Lm_5_10_Lm4	Listeria	PRJNA561589	SAMN12718226	SRR10088745
Lm_5_30_Lm6	Listeria	PRJNA561589	SAMN12718228	SRR10088746
Ec_Self_10_Ec5	Escherichia coli	PRJNA561589	SAMN12718229	SRR10088747
Ec_Se_30_Ec7	Escherichia coli	PRJNA561589	SAMN12718227	SRR10088748
Lm_5_10_Lm2	Listeria	PRJNA561589	SAMN12718230	SRR10088759
Lm_5_40_Lm2	Listeria	PRJNA561589	SAMN12718231	SRR10088760
Ec_Self_0_Ec3	Escherichia coli	PRJNA561589	SAMN12718232	SRR10088761
Ec_Se_20_Ec6	Escherichia coli	PRJNA561589	SAMN12718234	SRR10088762
Lm_5_10_Lm3	Listeria	PRJNA561589	SAMN12718233	SRR10088763
Ec_Se_40_Ec1	Escherichia coli	PRJNA561589	SAMN12718238	SRR10088788
Ec_Se_20_Ec5	Escherichia coli	PRJNA561589	SAMN12718237	SRR10088789
Lm_5_40_Lm5	Listeria	PRJNA561589	SAMN12718236	SRR10088790
Ec_Self_0_Ec4	Escherichia coli	PRJNA561589	SAMN12718235	SRR10088791
Lm_5_40_Lm4	Listeria	PRJNA561589	SAMN12718247	SRR10088792
Lm_5_10_Lm5	Listeria	PRJNA561589	SAMN12718245	SRR10088793
Lm_5_10_Lm6	Listeria	PRJNA561589	SAMN12718251	SRR10088794
Ec_Se_40_Ec2	Escherichia coli	PRJNA561589	SAMN12718243	SRR10088795
Ec_Self_10_Ec8	Escherichia coli	PRJNA561589	SAMN12718249	SRR10088796
Ec_Self_10_Ec7	Escherichia coli	PRJNA561589	SAMN12718371	SRR10088798
Ec_5_40_Ec3	Escherichia coli	PRJNA561589	SAMN12718369	SRR10088799
Lm_5_40_Lm3	Listeria	PRJNA561589	SAMN12718373	SRR10088800
Ec_Self_0_Ec6	Escherichia coli	PRJNA561589	SAMN12718367	SRR10088801
Ec_Self_0_Ec5	Escherichia coli	PRJNA561589	SAMN12718436	SRR10088802
Lm_5_30_Lm5	Listeria	PRJNA561589	SAMN12718434	SRR10088803
Lm_5_10_Lm8	Listeria	PRJNA561589	SAMN12718432	SRR10088804
Ec_Se_20_Ec8	Escherichia coli	PRJNA561589	SAMN12718440	SRR10088805
Lm_5_20_Lm1	Listeria	PRJNA561589	SAMN12718437	SRR10088806
Ec_Self_20_Ec1	Escherichia coli	PRJNA561589	SAMN12718554	SRR10088807
Ec_Se_40_Ec3	Escherichia coli	PRJNA561589	SAMN12718559	SRR10088808
Lm_5_10_Lm7	Listeria	PRJNA561589	SAMN12718555	SRR10088809
Ec_Se_20_Ec7	Escherichia coli	PRJNA561589	SAMN12718557	SRR10088810
Ec_Se_20_Ec4	Escherichia coli	PRJNA561589	SAMN12718552	SRR10088811
Lm_0.05_50_Lm8	Listeria	PRJNA561589	SAMN12718621	SRR10088812
Lm_0.5_50_Lm8	Listeria	PRJNA561589	SAMN12718617	SRR10088813
Ec_Self_0_Ec2	Escherichia coli	PRJNA561589	SAMN12718623	SRR10088814
Lm_0.05_50_Lm7	Listeria	PRJNA561589	SAMN12718613	SRR10088815
Lm_0.5_20_Lm3	Listeria	PRJNA561589	SAMN12718619	SRR10088816
Lm_0.5_30_Lm3	Listeria	PRJNA561589	SAMN12718615	SRR10088817
Ec_5_30_Ec5	Escherichia coli	PRJNA561589	SAMN12718701	SRR10088818
Lm_0.5_50_Lm7	Listeria	PRJNA561589	SAMN12718707	SRR10088819
Lm_0.5_30_Lm2	Listeria	PRJNA561589	SAMN12718703	SRR10088820
Lm_0.05_50_Lm4	Listeria	PRJNA561589	SAMN12718700	SRR10088821
Lm_0.05_40_Lm4	Listeria	PRJNA561589	SAMN12718705	SRR10088822
Lm_0.5_30_Lm1	Listeria	PRJNA561589	SAMN12718757	SRR10088823
Lm_0.5_40_Lm8	Listeria	PRJNA561589	SAMN12718758	SRR10088824
Lm_0.5_50_Lm1	Listeria	PRJNA561589	SAMN12718760	SRR10088825
Lm_0.05_40_Lm3	Listeria	PRJNA561589	SAMN12718756	SRR10088826
Ec_Se_20_Ec2	Escherichia coli	PRJNA561589	SAMN12718765	SRR10088827
Lm_0.5_40_Lm7	Listeria	PRJNA561589	SAMN12718759	SRR10088828
Lm_0.5_50_Lm3	Listeria	PRJNA561589	SAMN12718766	SRR10088829
Lm_0.05_50_Lm5	Listeria	PRJNA561589	SAMN12718769	SRR10088830
Lm_0.5_50_Lm2	Listeria	PRJNA561589	SAMN12718770	SRR10088831
Ec_Self_10_Ec2	Escherichia coli	PRJNA561589	SAMN12718768	SRR10088832
Ec_Se_30_Ec5	Escherichia coli	PRJNA561589	SAMN12718767	SRR10088833
Ec_Se_30_Ec4	Escherichia coli	PRJNA561589	SAMN12718774	SRR10088838
Lm_0.5_50_Lm6	Listeria	PRJNA561589	SAMN12718772	SRR10088839
Lm_0.05_50_Lm3	Listeria	PRJNA561589	SAMN12718775	SRR10088840
Lm_0.5_50_Lm5	Listeria	PRJNA561589	SAMN12718771	SRR10088841
Lm_5_30_Lm2	Listeria	PRJNA561589	SAMN12718776	SRR10088842
Lm_5_30_Lm1	Listeria	PRJNA561589	SAMN12718773	SRR10088843
Lm_0.5_40_Lm6	Listeria	PRJNA561589	SAMN12718781	SRR10088844
Lm_0.05_50_Lm6	Listeria	PRJNA561589	SAMN12718777	SRR10088845
Lm_0.05_50_Lm2	Listeria	PRJNA561589	SAMN12718782	SRR10088846
Lm_0.5_50_Lm4	Listeria	PRJNA561589	SAMN12718778	SRR10088847
Ec_Se_20_Ec3	Escherichia coli	PRJNA561589	SAMN12718779	SRR10088848
Lm_0.5_40_Lm2	Listeria	PRJNA561589	SAMN12718787	SRR10089164
Lm_0.5_40_Lm5	Listeria	PRJNA561589	SAMN12718792	SRR10089165
Ec_5_40_Ec1	Escherichia coli	PRJNA561589	SAMN12718788	SRR10089166
Lm_0.05_50_Lm1	Listeria	PRJNA561589	SAMN12718790	SRR10089167
Lm_0.5_40_Lm4	Listeria	PRJNA561589	SAMN12718791	SRR10089168
Lm_0.5_40_Lm3	Listeria	PRJNA561589	SAMN12718789	SRR10089169
Lm_0.05_40_Lm8	Listeria	PRJNA561589	SAMN12718807	SRR10089692
Ec_5_30_Ec8	Escherichia coli	PRJNA561589	SAMN12718805	SRR10089693
Lm_0.5_40_Lm1	Listeria	PRJNA561589	SAMN12718806	SRR10089694
Lm_0.05_40_Lm7	Listeria	PRJNA561589	SAMN12718804	SRR10089695
Lm_0.5_30_Lm8	Listeria	PRJNA561589	SAMN12718809	SRR10089696
Ec_5_30_Ec7	Escherichia coli	PRJNA561589	SAMN12718810	SRR10089697
Lm_0.05_40_Lm5	Listeria	PRJNA561589	SAMN12718808	SRR10089698
Ec_5_30_Ec6	Escherichia coli	PRJNA561589	SAMN12718823	SRR10089699
Lm_0.5_30_Lm4	Listeria	PRJNA561589	SAMN12718824	SRR10089700
Lm_0.5_30_Lm7	Listeria	PRJNA561589	SAMN12718825	SRR10089701
Lm_Ec_20_Lm1	Listeria	PRJNA561589	SAMN12718836	SRR10089702
Lm_Ec_10_Lm7	Listeria	PRJNA561589	SAMN12718835	SRR10089703
Se_Lm_30_Se5	Salmonella enterica	PRJNA561589	SAMN12718826	SRR10089704
Lm_Ec_20_Lm2	Listeria	PRJNA561589	SAMN12718827	SRR10089705
Se_Lm_30_Se6	Salmonella enterica	PRJNA561589	SAMN12718828	SRR10089706
Se_Lm_30_Se3	Salmonella enterica	PRJNA561589	SAMN12718838	SRR10089707
Lm_Ec_10_Lm6	Listeria	PRJNA561589	SAMN12718839	SRR10089708
Lm_Ec_10_Lm5	Listeria	PRJNA561589	SAMN12718837	SRR10089709
Se_Lm_30_Se4	Salmonella enterica	PRJNA561589	SAMN12718840	SRR10089710

Table S6: NCBI accession numbers for data generated during this study. (continued)

Strain Name	Organism	BioProject	NCBI BioSample	SRA Run
Lm_Ec_10_Lm4	Listeria	PRJNA561589	SAMN12718937	SRR10089717
Se_Lm_30_Se1	Salmonella enterica	PRJNA561589	SAMN12718936	SRR10089718
Se_Lm_30_Se2	Salmonella enterica	PRJNA561589	SAMN12718938	SRR10089719
Se_Self_50_Se1	Salmonella enterica	PRJNA561589	SAMN12718934	SRR10089720
Lm_Ec_10_Lm8	Listeria	PRJNA561589	SAMN12718935	SRR10089721
Se_Self_40_Se7	Salmonella enterica	PRJNA561589	SAMN12718950	SRR10089722
Se_Self_40_Se6	Salmonella enterica	PRJNA561589	SAMN12718948	SRR10089723
Lm_0.5_10_Lm2	Listeria	PRJNA561589	SAMN12718949	SRR10089724
Lm_0.5_10_Lm1	Listeria	PRJNA561589	SAMN12718951	SRR10089725
Lm_0.5_10_Lm3	Listeria	PRJNA561589	SAMN12718983	SRR10089726
Lm_0.5_10_Lm8	Listeria	PRJNA561589	SAMN12718985	SRR10089727
Se_Self_40_Se5	Salmonella enterica	PRJNA561589	SAMN12718982	SRR10089728
Lm_Ec_10_Lm3	Listeria	PRJNA561589	SAMN12718981	SRR10089729
Se_Lm_40_Se1	Salmonella enterica	PRJNA561589	SAMN12718984	SRR10089730
Lm_0.5_10_Lm4	Listeria	PRJNA561589	SAMN12718990	SRR10089731
Lm_Ec_20_Lm7	Listeria	PRJNA561589	SAMN12718989	SRR10089732
Lm_Ec_20_Lm8	Listeria	PRJNA561589	SAMN12718991	SRR10089733
Se_Self_40_Se8	Salmonella enterica	PRJNA561589	SAMN12718986	SRR10089734
Lm_Se_30_Lm8	Listeria	PRJNA561589	SAMN12718987	SRR10089735
Se_Self_50_Se4	Salmonella enterica	PRJNA561589	SAMN12718988	SRR10089736
Lm_0.5_10_Lm7	Listeria	PRJNA561589	SAMN12718992	SRR10089737
Se_Self_50_Se3	Salmonella enterica	PRJNA561589	SAMN12718996	SRR10089738
Lm_Self_40_Lm6	Listeria	PRJNA561589	SAMN12718993	SRR10089739
Lm_Ec_20_Lm6	Listeria	PRJNA561589	SAMN12718998	SRR10089740
Lm_Self_40_Lm7	Listeria	PRJNA561589	SAMN12718997	SRR10089741
Se_Self_50_Se2	Salmonella enterica	PRJNA561589	SAMN12718994	SRR10089742
Se_0.5_20_Se3	Salmonella enterica	PRJNA561589	SAMN12718995	SRR10089743
Lm_0.5_10_Lm5	Listeria	PRJNA561589	SAMN12719002	SRR10089744
Lm_Se_30_Lm7	Listeria	PRJNA561589	SAMN12718999	SRR10089745
Lm_Self_40_Lm5	Listeria	PRJNA561589	SAMN12719000	SRR10089746
Lm_0.5_10_Lm6	Listeria	PRJNA561589	SAMN12719001	SRR10089747
Se_0.5_20_Se2	Salmonella enterica	PRJNA561589	SAMN12719003	SRR10089748
Se_Ec_50_Se3	Salmonella enterica	PRJNA561589	SAMN12719006	SRR10089749
Se_Ec_50_Se4	Salmonella enterica	PRJNA561589	SAMN12719005	SRR10089750
Se_Ec_50_Se5	Salmonella enterica	PRJNA561589	SAMN12719004	SRR10089751
Se_Lm_30_Se7	Salmonella enterica	PRJNA561589	SAMN12719010	SRR10089776
Lm_Se_30_Lm2	Listeria	PRJNA561589	SAMN12719009	SRR10089777
Lm_Se_30_Lm3	Listeria	PRJNA561589	SAMN12719011	SRR10089778
Se_Ec_50_Se2	Salmonella enterica	PRJNA561589	SAMN12719008	SRR10089779
Se_0.5_10_Se7	Salmonella enterica	PRJNA561589	SAMN12719007	SRR10089780
Lm_Se_30_Lm1	Listeria	PRJNA561589	SAMN12719025	SRR10089781
Lm_Ec_20_Lm4	Listeria	PRJNA561589	SAMN12719022	SRR10089782
Lm_Self_50_Lm6	Listeria	PRJNA561589	SAMN12719023	SRR10089783
Lm_Ec_20_Lm5	Listeria	PRJNA561589	SAMN12719021	SRR10089784
Se_0.5_10_Se6	Salmonella enterica	PRJNA561589	SAMN12719024	SRR10089785
Lm_Ec_20_Lm3	Listeria	PRJNA561589	SAMN12719020	SRR10089786
Se_Lm_30_Se8	Salmonella enterica	PRJNA561589	SAMN12719029	SRR10089787
Se_Self_30_Se3	Salmonella enterica	PRJNA561589	SAMN12719026	SRR10089788
Lm_Se_30_Lm5	Listeria	PRJNA561589	SAMN12719027	SRR10089789
Se_Ec_30_Se6	Salmonella enterica	PRJNA561589	SAMN12719028	SRR10089790
Se_0.5_20_Se1	Salmonella enterica	PRJNA561589	SAMN12719039	SRR10089791
Se_Ec_30_Se5	Salmonella enterica	PRJNA561589	SAMN12719038	SRR10089792
Se_Ec_30_Se8	Salmonella enterica	PRJNA561589	SAMN12719036	SRR10089793
Se_Ec_40_Se2	Salmonella enterica	PRJNA561589	SAMN12719037	SRR10089794
Se_Ec_40_Se1	Salmonella enterica	PRJNA561589	SAMN12719044	SRR10089795
Lm_Se_30_Lm6	Listeria	PRJNA561589	SAMN12719043	SRR10089796
Se_Ec_30_Se7	Salmonella enterica	PRJNA561589	SAMN12719042	SRR10089797
Lm_0.5_20_Lm7	Listeria	PRJNA561589	SAMN12719041	SRR10089798
Lm_0.5_20_Lm8	Listeria	PRJNA561589	SAMN12719040	SRR10089799
Lm_Se_40_Lm1	Listeria	PRJNA561589	SAMN12719048	SRR10089800
Lm_Self_40_Lm8	Listeria	PRJNA561589	SAMN12719045	SRR10089801
Lm_Self_50_Lm1	Listeria	PRJNA561589	SAMN12719047	SRR10089802
Lm_0.5_20_Lm4	Listeria	PRJNA561589	SAMN12719046	SRR10089803
Lm_0.5_20_Lm5	Listeria	PRJNA561589	SAMN12719050	SRR10089804
Lm_0.5_20_Lm6	Listeria	PRJNA561589	SAMN12719049	SRR10089805
Lm_0.5_20_Lm2	Listeria	PRJNA561589	SAMN12719052	SRR10089806
Lm_0.5_20_Lm1	Listeria	PRJNA561589	SAMN12719053	SRR10089807
Se_0.5_10_Se8	Salmonella enterica	PRJNA561589	SAMN12719055	SRR10089808
Se_0.5_20_Se4	Salmonella enterica	PRJNA561589	SAMN12719054	SRR10089809
Lm_0.5_30_Lm6	Listeria	PRJNA561589	SAMN12719051	SRR10089810
Lm_0.05_40_Lm6	Listeria	PRJNA561589	SAMN12719056	SRR10089811
Se_Ec_30_Se4	Salmonella enterica	PRJNA561589	SAMN12719059	SRR10089812
Se_Self_20_Se8	Salmonella enterica	PRJNA561589	SAMN12719057	SRR10089813
Lm_Se_30_Lm4	Listeria	PRJNA561589	SAMN12719060	SRR10089814
Se_Self_30_Se2	Salmonella enterica	PRJNA561589	SAMN12719058	SRR10089815
Lm_Self_10_Lm8	Listeria	PRJNA561589	SAMN12719061	SRR10089816
Lm_Ec_40_Lm5	Listeria	PRJNA561589	SAMN12719063	SRR10089817
Se_0.5_10_Se3	Salmonella enterica	PRJNA561589	SAMN12719064	SRR10089818
Se_Self_10_Se5	Salmonella enterica	PRJNA561589	SAMN12719062	SRR10089819
Se_Self_30_Se1	Salmonella enterica	PRJNA561589	SAMN12719065	SRR10089820
Lm_Se_20_Lm3	Listeria	PRJNA561589	SAMN12719068	SRR10089821
Se_0.5_40_Se3	Salmonella enterica	PRJNA561589	SAMN12719066	SRR10089822
Se_0.5_10_Se2	Salmonella enterica	PRJNA561589	SAMN12719078	SRR10089823
Lm_Se_20_Lm4	Listeria	PRJNA561589	SAMN12719069	SRR10089824
Se_Lm_10_Se3	Salmonella enterica	PRJNA561589	SAMN12719067	SRR10089825
Se_Ec_30_Se3	Salmonella enterica	PRJNA561589	SAMN12719080	SRR10089826
Lm_Self_30_Lm7	Listeria	PRJNA561589	SAMN12719081	SRR10089827
Se_Self_20_Se7	Salmonella enterica	PRJNA561589	SAMN12719082	SRR10089828
Se_5_30_Se7	Salmonella enterica	PRJNA561589	SAMN12719079	SRR10089829
Lm_Se_20_Lm8	Listeria	PRJNA561589	SAMN12719086	SRR10089830
Se_0.05_20_Se5	Salmonella enterica	PRJNA561589	SAMN12719087	SRR10089831
Se_Self_50_Se8	Salmonella enterica	PRJNA561589	SAMN12719084	SRR10089832
Se_0.5_10_Se5	Salmonella enterica	PRJNA561589	SAMN12719083	SRR10089833
Se_Ec_30_Se2	Salmonella enterica	PRJNA561589	SAMN12719085	SRR10089834
Se_Self_20_Se6	Salmonella enterica	PRJNA561589	SAMN12719091	SRR10089835
Se_Lm_10_Se2	Salmonella enterica	PRJNA561589	SAMN12719090	SRR10089836
Lm_Self_0_Lm3	Listeria	PRJNA561589	SAMN12719088	SRR10089837
Se_0.5_50_Se5	Salmonella enterica	PRJNA561589	SAMN12719089	SRR10089838
Se_Ec_30_Se1	Salmonella enterica	PRJNA561589	SAMN12719096	SRR10089855
Se_5_30_Se3	Salmonella enterica	PRJNA561589	SAMN12719094	SRR10089856
Lm_Se_20_Lm7	Listeria	PRJNA561589	SAMN12719095	SRR10089857
Se_0.05_20_Se4	Salmonella enterica	PRJNA561589	SAMN12719092	SRR10089858
Lm_Se_20_Lm6	Listeria	PRJNA561589	SAMN12719093	SRR10089859
Lm_Self_30_Lm5	Listeria	PRJNA561589	SAMN12719101	SRR10089864
Lm_Self_0_Lm1	Listeria	PRJNA561589	SAMN12719102	SRR10089865
Se_Ec_20_Se8	Salmonella enterica	PRJNA561589	SAMN12719103	SRR10089866
Lm_Self_0_Lm2	Listeria	PRJNA561589	SAMN12719104	SRR10089867
Se_Self_20_Se5	Salmonella enterica	PRJNA561589	SAMN12719105	SRR10089868
Se_0.05_20_Se3	Salmonella enterica	PRJNA561589	SAMN12719110	SRR10089871
Se_Self_20_Se4	Salmonella enterica	PRJNA561589	SAMN12719109	SRR10089872
Lm_Se_20_Lm5	Listeria	PRJNA561589	SAMN12719108	SRR10089873
Se_Ec_20_Se7	Salmonella enterica	PRJNA561589	SAMN12719111	SRR10089874
Se_0.5_40_Se1	Salmonella enterica	PRJNA561589	SAMN12719112	SRR10089875
Se_0.5_40_Se2	Salmonella enterica	PRJNA561589	SAMN12719113	SRR10089876
Lm_Se_50_Lm8	Listeria	PRJNA561589	SAMN12719114	SRR10089877
Se_Ec_20_Se6	Salmonella enterica	PRJNA561589	SAMN12719117	SRR10089878
Lm_Self_30_Lm4	Listeria	PRJNA561589	SAMN12719116	SRR10089879
Se_Self_20_Se3	Salmonella enterica	PRJNA561589	SAMN12719115	SRR10089880
Se_Self_0_Se7	Salmonella enterica	PRJNA561589	SAMN12719124	SRR10089884
Se_Self_10_Se4	Salmonella enterica	PRJNA561589	SAMN12719123	SRR10089885
Se_0.5_10_Se4	Salmonella enterica	PRJNA561589	SAMN12719126	SRR10089886
Lm_Self_30_Lm6	Listeria	PRJNA561589	SAMN12719125	SRR10089887
Se_Lm_20_Se2	Salmonella enterica	PRJNA561589	SAMN12719122	SRR10089888
Se_Ec_10_Se8	Salmonella enterica	PRJNA561589	SAMN12719121	SRR10089889
Se_Lm_40_Se5	Salmonella enterica	PRJNA561589	SAMN12719130	SRR10089890
Se_Ec_10_Se7	Salmonella enterica	PRJNA561589	SAMN12719129	SRR10089891
Se_5_40_Se2	Salmonella enterica	PRJNA561589	SAMN12719128	SRR10089892
Se_Lm_20_Se1	Salmonella enterica	PRJNA561589	SAMN12719127	SRR10089893
Se_Lm_10_Se8	Salmonella enterica	PRJNA561589	SAMN12719132	SRR10089894
Se_Ec_10_Se6	Salmonella enterica	PRJNA561589	SAMN12719131	SRR10089895
Se_Self_10_Se3	Salmonella enterica	PRJNA561589	SAMN12719133	SRR10089896
Se_Self_10_Se2	Salmonella enterica	PRJNA561589	SAMN12719145	SRR10089897
Se_Ec_10_Se5	Salmonella enterica	PRJNA561589	SAMN12719144	SRR10089898
Se_Lm_40_Se4	Salmonella enterica	PRJNA561589	SAMN12719146	SRR10089899
Se_5_40_Se1	Salmonella enterica	PRJNA561589	SAMN12719143	SRR10089900
Lm_Self_30_Lm3	Listeria	PRJNA561589	SAMN12719150	SRR10089901
Se_Self_0_Se6	Salmonella enterica	PRJNA561589	SAMN12719147	SRR10089902
Se_5_40_Se3	Salmonella enterica	PRJNA561589	SAMN12719149	SRR10089903
Se_Self_10_Se1	Salmonella enterica	PRJNA561589	SAMN12719151	SRR10089904
Se_Ec_10_Se2	Salmonella enterica	PRJNA561589	SAMN12719148	SRR10089905
Lm_Se_20_Lm2	Listeria	PRJNA561589	SAMN12719155	SRR10089906
Se_0.5_30_Se8	Salmonella enterica	PRJNA561589	SAMN12719152	SRR10089907
Se_5_30_Se8	Salmonella enterica	PRJNA561589	SAMN12719153	SRR10089908
Se_Lm_10_Se5	Salmonella enterica	PRJNA561589	SAMN12719154	SRR10089909
Se_0.05_50_Se8	Salmonella enterica	PRJNA561589	SAMN12719157	SRR10089916
Lm_Se_20_Lm1	Listeria	PRJNA561589	SAMN12719159	SRR10089917
Se_0.05_20_Se6	Salmonella enterica	PRJNA561589	SAMN12719158	SRR10089918
Se_0.5_10_Se1	Salmonella enterica	PRJNA561589	SAMN12719160	SRR10089919
Se_Lm_10_Se4	Salmonella enterica	PRJNA561589	SAMN12719156	SRR10089920
Se_0.05_50_Se7	Salmonella enterica	PRJNA561589	SAMN12719163	SRR10089921
Lm_Self_0_Lm4	Listeria	PRJNA561589	SAMN12719164	SRR10089922
Se_Lm_40_Se2	Salmonella enterica	PRJNA561589	SAMN12719162	SRR10089923
Lm_Se_10_Lm8	Listeria	PRJNA561589	SAMN12719161	SRR10089924
Se_Ec_10_Se4	Salmonella enterica	PRJNA561589	SAMN12719174	SRR10089932
Lm_Se_10_Lm7	Listeria	PRJNA561589	SAMN12719175	SRR10089933
Se_Lm_10_Se6	Salmonella enterica	PRJNA561589	SAMN12719173	SRR10089934
Se_Lm_10_Se7	Salmonella enterica	PRJNA561589	SAMN12719172	SRR10089935
Lm_Self_30_Lm8	Listeria	PRJNA561589	SAMN12719179	SRR10089936
Se_Lm_40_Se3	Salmonella enterica	PRJNA561589	SAMN12719177	SRR10089937
Se_Ec_10_Se3	Salmonella enterica	PRJNA561589	SAMN12719180	SRR10089938
Se_0.5_50_Se3	Salmonella enterica	PRJNA561589	SAMN12719176	SRR10089939
Se_Self_0_Se8	Salmonella enterica	PRJNA561589	SAMN12719178	SRR10089940
Se_0.05_30_Se5	Salmonella enterica	PRJNA561589	SAMN12719185	SRR10089941
Lm_Ec_40_Lm2	Listeria	PRJNA561589	SAMN12719186	SRR10089942
Lm_Self_10_Lm5	Listeria	PRJNA561589	SAMN12719184	SRR10089943
Se_0.05_30_Se4	Salmonella enterica	PRJNA561589	SAMN12719181	SRR10089944
Lm_Ec_40_Lm1	Listeria	PRJNA561589	SAMN12719182	SRR10089945
Se_0.5_50_Se2	Salmonella enterica	PRJNA561589	SAMN12719183	SRR10089946
Lm_Self_10_Lm4	Listeria	PRJNA561589	SAMN12719190	SRR10089949
Se_Lm_50_Se8	Salmonella enterica	PRJNA561589	SAMN12719191	SRR10089950
Se_Lm_50_Se7	Salmonella enterica	PRJNA561589	SAMN12719189	SRR10089951
Se_Lm_50_Se6	Salmonella enterica	PRJNA561589	SAMN12719193	SRR10089960
Se_Lm_50_Se5	Salmonella enterica	PRJNA561589	SAMN12719196	SRR10089961
Lm_Self_10_Lm3	Listeria	PRJNA561589	SAMN12719197	SRR10089962
Se_5_50_Se3	Salmonella enterica	PRJNA561589	SAMN12719192	SRR10089963
Se_5_50_Se4	Salmonella enterica	PRJNA561589	SAMN12719194	SRR10089964
Se_0.5_20_Se6	Salmonella enterica	PRJNA561589	SAMN12719198	SRR10089966
Se_0.05_30_Se3	Salmonella enterica	PRJNA561589	SAMN12719199	SRR10089967
Lm_Ec_30_Lm8	Listeria	PRJNA561589	SAMN12719201	SRR10089968
Se_0.5_50_Se1	Salmonella enterica	PRJNA561589	SAMN12719200	SRR10089969
Se_Ec_50_Se1	Salmonella enterica	PRJNA561589	SAMN12719202	SRR10089970
Lm_Self_50_Lm5	Listeria	PRJNA561589	SAMN12719205	SRR10089971
Lm_Self_10_Lm2	Listeria	PRJNA561589	SAMN12719206	SRR10089972
Se_Self_0_Se2	Salmonella enterica	PRJNA561589	SAMN12719203	SRR10089973
Lm_Ec_50_Lm2	Listeria	PRJNA561589	SAMN12719204	SRR10089974
Se_5_50_Se7	Salmonella enterica	PRJNA561589	SAMN12719207	SRR10089975
Lm_Self_50_Lm4	Listeria	PRJNA561589	SAMN12719211	SRR10089976
Se_5_50_Se6	Salmonella enterica	PRJNA561589	SAMN12719208	SRR10089977
Lm_Se_40_Lm3	Listeria	PRJNA561589	SAMN12719209	SRR10090170
Se_0.05_40_Se3	Salmonella enterica	PRJNA561589	SAMN12719210	SRR10090172
Lm_Se_40_Lm2	Listeria	PRJNA561589	SAMN12719226	SRR10090182
Lm_Ec_50_Lm1	Listeria	PRJNA561589	SAMN12719224	SRR10090183
Se_Self_0_Se1	Salmonella enterica	PRJNA561589	SAMN12719227	SRR10090184
Se_Ec_40_Se8	Salmonella enterica	PRJNA561589	SAMN12719225	SRR10090185
Se_0.5_50_Se8	Salmonella enterica	PRJNA561589	SAMN12719241	SRR10090194
Se_Ec_40_Se7	Salmonella enterica	PRJNA561589	SAMN12719240	SRR10090195
Lm_Self_50_Lm3	Listeria	PRJNA561589	SAMN12719236	SRR10090196
Lm_Self_50_Lm2	Listeria	PRJNA561589	SAMN12719237	SRR10090197
Se_5_50_Se5	Salmonella enterica	PRJNA561589	SAMN12719238	SRR10090198
Se_0.05_40_Se2	Salmonella enterica	PRJNA561589	SAMN12719239	SRR10090199
Se_Ec_40_Se6	Salmonella enterica	PRJNA561589	SAMN12719250	SRR10090207
Lm_Self_20_Lm3	Listeria	PRJNA561589	SAMN12719249	SRR10090208
Se_0.5_20_Se5	Salmonella enterica	PRJNA561589	SAMN12719252	SRR10090209
Lm_Ec_40_Lm8	Listeria	PRJNA561589	SAMN12719251	SRR10090210
Se_Ec_40_Se4	Salmonella enterica	PRJNA561589	SAMN12719261	SRR10090211
Se_Self_20_Se2	Salmonella enterica	PRJNA561589	SAMN12719262	SRR10090212
Lm_Ec_40_Lm7	Listeria	PRJNA561589	SAMN12719263	SRR10090213
Lm_Self_20_Lm2	Listeria	PRJNA561589	SAMN12719260	SRR10090214
Se_Ec_40_Se5	Salmonella enterica	PRJNA561589	SAMN12719264	SRR10090215
Se_0.05_40_Se1	Salmonella enterica	PRJNA561589	SAMN12719271	SRR10090264
Se_Ec_40_Se3	Salmonella enterica	PRJNA561589	SAMN12719268	SRR10090265
Se_0.5_50_Se7	Salmonella enterica	PRJNA561589	SAMN12719273	SRR10090266
Se_Self_20_Se1	Salmonella enterica	PRJNA561589	SAMN12719266	SRR10090267
Se_Ec_20_Se4	Salmonella enterica	PRJNA561589	SAMN12719349	SRR10090268
Se_Self_0_Se5	Salmonella enterica	PRJNA561589	SAMN12719350	SRR10090269
Se_Self_0_Se4	Salmonella enterica	PRJNA561589	SAMN12719348	SRR10090270
Se_Ec_20_Se5	Salmonella enterica	PRJNA561589	SAMN12719351	SRR10090271
Se_Self_0_Se3	Salmonella enterica	PRJNA561589	SAMN12719636	SRR10090281
Se_5_50_Se8	Salmonella enterica	PRJNA561589	SAMN12719634	SRR10090282
Se_Ec_20_Se3	Salmonella enterica	PRJNA561589	SAMN12719635	SRR10090283
Se_Ec_10_Se1	Salmonella enterica	PRJNA561589	SAMN12719637	SRR10090284
Lm_Ec_40_Lm6	Listeria	PRJNA561589	SAMN12719645	SRR10090286
Se_Self_10_Se8	Salmonella enterica	PRJNA561589	SAMN12719646	SRR10090288
Se_Self_10_Se7	Salmonella enterica	PRJNA561589	SAMN12719642	SRR10090289
Se_Ec_20_Se2	Salmonella enterica	PRJNA561589	SAMN12719644	SRR10090290
Lm_Self_20_Lm1	Listeria	PRJNA561589	SAMN12719643	SRR10090291
Se_Ec_20_Se1	Salmonella enterica	PRJNA561589	SAMN12719663	SRR10090305
Se_Self_10_Se6	Salmonella enterica	PRJNA561589	SAMN12719661	SRR10090306
Se_0.5_50_Se6	Salmonella enterica	PRJNA561589	SAMN12719662	SRR10090307
Se_0.05_30_Se8	Salmonella enterica	PRJNA561589	SAMN12719660	SRR10090308
Lm_Se_50_Lm7	Listeria	PRJNA561589	SAMN12719659	SRR10090309
Lm_Ec_30_Lm6	Listeria	PRJNA561589	SAMN12719665	SRR10090310
Se_0.5_40_Se8	Salmonella enterica	PRJNA561589	SAMN12719666	SRR10090311
Se_Lm_20_Se6	Salmonella enterica	PRJNA561589	SAMN12719664	SRR10090312
Se_Self_50_Se7	Salmonella enterica	PRJNA561589	SAMN12719668	SRR10090313
Se_Lm_40_Se7	Salmonella enterica	PRJNA561589	SAMN12719667	SRR10090314
Lm_Self_10_Lm1	Listeria	PRJNA561589	SAMN12719670	SRR10090315
Se_5_40_Se4	Salmonella enterica	PRJNA561589	SAMN12719671	SRR10090316
Se_0.05_30_Se2	Salmonella enterica	PRJNA561589	SAMN12719673	SRR10090317
Se_Lm_20_Se5	Salmonella enterica	PRJNA561589	SAMN12719672	SRR10090318
Se_Lm_40_Se6	Salmonella enterica	PRJNA561589	SAMN12719694	SRR10090319
Se_0.5_40_Se7	Salmonella enterica	PRJNA561589	SAMN12719691	SRR10090320
Se_Lm_20_Se4	Salmonella enterica	PRJNA561589	SAMN12719695	SRR10090321
Lm_Se_10_Lm1	Listeria	PRJNA561589	SAMN12719689	SRR10090322
Lm_Ec_50_Lm8	Listeria	PRJNA561589	SAMN12719785	SRR10090323
Se_0.05_30_Se7	Salmonella enterica	PRJNA561589	SAMN12719824	SRR10090324
Se_5_50_Se2	Salmonella enterica	PRJNA561589	SAMN12719821	SRR10090325
Se_0.05_50_Se1	Salmonella enterica	PRJNA561589	SAMN12719826	SRR10090326
Se_Lm_50_Se4	Salmonella enterica	PRJNA561589	SAMN12719850	SRR10090394
Lm_Self_10_Lm7	Listeria	PRJNA561589	SAMN12719852	SRR10090395
Se_0.05_30_Se6	Salmonella enterica	PRJNA561589	SAMN12719851	SRR10090396
Lm_Ec_40_Lm4	Listeria	PRJNA561589	SAMN12719849	SRR10090397
Se_0.5_50_Se4	Salmonella enterica	PRJNA561589	SAMN12719853	SRR10090404
Se_Lm_50_Se3	Salmonella enterica	PRJNA561589	SAMN12719856	SRR10090405
Lm_Self_10_Lm6	Listeria	PRJNA561589	SAMN12719857	SRR10090406
Lm_Ec_40_Lm3	Listeria	PRJNA561589	SAMN12719854	SRR10090407
Se_5_50_Se1	Salmonella enterica	PRJNA561589	SAMN12719858	SRR10090408
Se_0.05_40_Se7	Salmonella enterica	PRJNA561589	SAMN12719855	SRR10090409
Lm_Ec_50_Lm7	Listeria	PRJNA561589	SAMN12719863	SRR10090410
Se_0.05_40_Se6	Salmonella enterica	PRJNA561589	SAMN12719859	SRR10090411
Lm_Ec_50_Lm6	Listeria	PRJNA561589	SAMN12719861	SRR10090412
Lm_Ec_50_Lm4	Listeria	PRJNA561589	SAMN12719860	SRR10090413
Lm_Ec_50_Lm5	Listeria	PRJNA561589	SAMN12719862	SRR10090414
Lm_Se_10_Lm3	Listeria	PRJNA561589	SAMN12719882	SRR10090542
Se_0.05_50_Se6	Salmonella enterica	PRJNA561589	SAMN12719867	SRR10090543
Lm_Se_10_Lm6	Listeria	PRJNA561589	SAMN12719868	SRR10090544
Lm_Se_10_Lm4	Listeria	PRJNA561589	SAMN12719881	SRR10090545
Se_0.05_40_Se8	Salmonella enterica	PRJNA561589	SAMN12719884	SRR10090546
Se_0.05_50_Se5	Salmonella enterica	PRJNA561589	SAMN12719883	SRR10090547
Se_0.05_50_Se3	Salmonella enterica	PRJNA561589	SAMN12719903	SRR10090673
Lm_Se_10_Lm2	Listeria	PRJNA561589	SAMN12719904	SRR10090674
Se_0.05_50_Se2	Salmonella enterica	PRJNA561589	SAMN12719902	SRR10090675
Se_0.05_40_Se5	Salmonella enterica	PRJNA561589	SAMN12719901	SRR10090676
Se_0.05_40_Se4	Salmonella enterica	PRJNA561589	SAMN12719909	SRR10090692
Lm_Se_10_Lm5	Listeria	PRJNA561589	SAMN12719906	SRR10090693
Se_0.05_50_Se4	Salmonella enterica	PRJNA561589	SAMN12719907	SRR10090694
Lm_Ec_50_Lm3	Listeria	PRJNA561589	SAMN12719908	SRR10090695
Se_0.5_30_Se5	Salmonella enterica	PRJNA561589	SAMN12719905	SRR10090696
Lm_Self_20_Lm8	Listeria	PRJNA561589	SAMN12719915	SRR10090697
Lm_Se_50_Lm2	Listeria	PRJNA561589	SAMN12719912	SRR10090698
Lm_Se_50_Lm3	Listeria	PRJNA561589	SAMN12719911	SRR10090699
Lm_Self_20_Lm7	Listeria	PRJNA561589	SAMN12719914	SRR10090700
Se_0.05_10_Se7	Salmonella enterica	PRJNA561589	SAMN12719913	SRR10090701
Se_0.5_30_Se4	Salmonella enterica	PRJNA561589	SAMN12719910	SRR10090702
Se_5_20_Se8	Salmonella enterica	PRJNA561589	SAMN12719956	SRR10090709
Se_0.5_30_Se3	Salmonella enterica	PRJNA561589	SAMN12719916	SRR10090710
Se_0.05_10_Se6	Salmonella enterica	PRJNA561589	SAMN12719959	SRR10090711
Lm_Se_50_Lm4	Listeria	PRJNA561589	SAMN12719958	SRR10090712
Lm_Self_30_Lm1	Listeria	PRJNA561589	SAMN12719957	SRR10090713
Se_5_30_Se5	Salmonella enterica	PRJNA561589	SAMN12719964	SRR10090795
Se_Self_50_Se5	Salmonella enterica	PRJNA561589	SAMN12719965	SRR10090796
Se_5_30_Se4	Salmonella enterica	PRJNA561589	SAMN12719963	SRR10090797
Se_5_30_Se1	Salmonella enterica	PRJNA561589	SAMN12719960	SRR10090798
Se_5_20_Se7	Salmonella enterica	PRJNA561589	SAMN12719969	SRR10090799
Se_5_20_Se6	Salmonella enterica	PRJNA561589	SAMN12719968	SRR10090800
Se_Self_50_Se6	Salmonella enterica	PRJNA561589	SAMN12719966	SRR10090801
Se_5_30_Se6	Salmonella enterica	PRJNA561589	SAMN12719967	SRR10090802
Se_Lm_20_Se3	Salmonella enterica	PRJNA561589	SAMN12719974	SRR10090803
Se_5_30_Se2	Salmonella enterica	PRJNA561589	SAMN12719973	SRR10090804
Se_0.05_30_Se1	Salmonella enterica	PRJNA561589	SAMN12719970	SRR10090805
Lm_Ec_30_Lm5	Listeria	PRJNA561589	SAMN12719971	SRR10090806
Lm_Self_0_Lm8	Listeria	PRJNA561589	SAMN12719972	SRR10090807
Se_0.05_20_Se8	Salmonella enterica	PRJNA561589	SAMN12719976	SRR10090808
Lm_Ec_30_Lm4	Listeria	PRJNA561589	SAMN12719977	SRR10090809
Se_0.5_40_Se6	Salmonella enterica	PRJNA561589	SAMN12719978	SRR10090810
Se_0.5_40_Se5	Salmonella enterica	PRJNA561589	SAMN12719975	SRR10090811
Lm_Ec_30_Lm2	Listeria	PRJNA561589	SAMN12719982	SRR10090812
Lm_Self_0_Lm7	Listeria	PRJNA561589	SAMN12719983	SRR10090813
Se_5_40_Se8	Salmonella enterica	PRJNA561589	SAMN12719980	SRR10090814
Lm_Self_0_Lm6	Listeria	PRJNA561589	SAMN12719979	SRR10090815
Lm_Ec_30_Lm3	Listeria	PRJNA561589	SAMN12719981	SRR10090816
Lm_Self_40_Lm4	Listeria	PRJNA561589	SAMN12719988	SRR10090817
Lm_Self_40_Lm3	Listeria	PRJNA561589	SAMN12719987	SRR10090818
Se_5_40_Se7	Salmonella enterica	PRJNA561589	SAMN12719986	SRR10090819
Se_Lm_50_Se1	Salmonella enterica	PRJNA561589	SAMN12719985	SRR10090820
Se_Lm_50_Se2	Salmonella enterica	PRJNA561589	SAMN12719989	SRR10090821
Lm_Self_40_Lm1	Listeria	PRJNA561589	SAMN12719984	SRR10090822
Se_Lm_20_Se8	Salmonella enterica	PRJNA561589	SAMN12719994	SRR10090823
Se_Lm_40_Se8	Salmonella enterica	PRJNA561589	SAMN12719991	SRR10090824
Se_5_40_Se5	Salmonella enterica	PRJNA561589	SAMN12719993	SRR10090825
Se_Lm_20_Se7	Salmonella enterica	PRJNA561589	SAMN12719995	SRR10090826
Lm_Self_40_Lm2	Listeria	PRJNA561589	SAMN12719992	SRR10090827
Se_5_40_Se6	Salmonella enterica	PRJNA561589	SAMN12719990	SRR10090828
Se_0.5_40_Se4	Salmonella enterica	PRJNA561589	SAMN12719997	SRR10090829
Lm_Self_0_Lm5	Listeria	PRJNA561589	SAMN12719998	SRR10090830
Lm_Ec_30_Lm7	Listeria	PRJNA561589	SAMN12719999	SRR10090831
Se_0.05_20_Se7	Salmonella enterica	PRJNA561589	SAMN12719996	SRR10090832
Se_0.05_10_Se3	Salmonella enterica	PRJNA561589	SAMN12720005	SRR10090833
Se_0.5_30_Se1	Salmonella enterica	PRJNA561589	SAMN12720001	SRR10090834
Se_0.05_10_Se2	Salmonella enterica	PRJNA561589	SAMN12720006	SRR10090835
Lm_Se_40_Lm7	Listeria	PRJNA561589	SAMN12720000	SRR10090836
Lm_Self_20_Lm4	Listeria	PRJNA561589	SAMN12720003	SRR10090837
Se_Ec_50_Se8	Salmonella enterica	PRJNA561589	SAMN12720002	SRR10090838
Lm_Se_40_Lm6	Listeria	PRJNA561589	SAMN12720007	SRR10090839
Se_0.05_10_Se1	Salmonella enterica	PRJNA561589	SAMN12720008	SRR10090840
Se_0.5_20_Se8	Salmonella enterica	PRJNA561589	SAMN12720011	SRR10090841
Lm_Se_40_Lm5	Listeria	PRJNA561589	SAMN12720010	SRR10090842
Lm_Self_50_Lm8	Listeria	PRJNA561589	SAMN12720009	SRR10090843
Se_0.5_20_Se7	Salmonella enterica	PRJNA561589	SAMN12720017	SRR10090844
Lm_Self_50_Lm7	Listeria	PRJNA561589	SAMN12720016	SRR10090845
Se_5_10_Se5	Salmonella enterica	PRJNA561589	SAMN12720013	SRR10090846
Lm_Se_40_Lm4	Listeria	PRJNA561589	SAMN12720014	SRR10090847
Se_5_10_Se7	Salmonella enterica	PRJNA561589	SAMN12720015	SRR10090848
Se_5_10_Se6	Salmonella enterica	PRJNA561589	SAMN12720021	SRR10090849
Lm_Self_20_Lm6	Listeria	PRJNA561589	SAMN12720018	SRR10090850
Se_5_10_Se4	Salmonella enterica	PRJNA561589	SAMN12720020	SRR10090851
Se_5_10_Se3	Salmonella enterica	PRJNA561589	SAMN12720019	SRR10090852
Se_0.5_30_Se2	Salmonella enterica	PRJNA561589	SAMN12720023	SRR10090895
Se_0.05_10_Se5	Salmonella enterica	PRJNA561589	SAMN12720027	SRR10090896
Lm_Se_50_Lm1	Listeria	PRJNA561589	SAMN12720024	SRR10090897
Lm_Self_20_Lm5	Listeria	PRJNA561589	SAMN12720025	SRR10090898
Se_5_10_Se2	Salmonella enterica	PRJNA561589	SAMN12720026	SRR10090899
Se_5_10_Se1	Salmonella enterica	PRJNA561589	SAMN12720301	SRR10090900
Se_0.5_30_Se7	Salmonella enterica	PRJNA561589	SAMN12720299	SRR10090901
Se_5_20_Se5	Salmonella enterica	PRJNA561589	SAMN12720302	SRR10090902
Lm_Se_40_Lm8	Listeria	PRJNA561589	SAMN12720300	SRR10090903
Se_5_20_Se4	Salmonella enterica	PRJNA561589	SAMN12720303	SRR10090904
Se_0.05_10_Se4	Salmonella enterica	PRJNA561589	SAMN12720305	SRR10090905
Lm_Se_50_Lm6	Listeria	PRJNA561589	SAMN12720307	SRR10090906
Se_0.05_20_Se2	Salmonella enterica	PRJNA561589	SAMN12720306	SRR10090907
Lm_Self_30_Lm2	Listeria	PRJNA561589	SAMN12720304	SRR10090908
Se_5_10_Se8	Salmonella enterica	PRJNA561589	SAMN12720312	SRR10090909
Se_0.05_20_Se1	Salmonella enterica	PRJNA561589	SAMN12720308	SRR10090910
Se_5_20_Se3	Salmonella enterica	PRJNA561589	SAMN12720311	SRR10090911
Se_Ec_50_Se6	Salmonella enterica	PRJNA561589	SAMN12720310	SRR10090912
Lm_Ec_30_Lm1	Listeria	PRJNA561589	SAMN12720405	SRR10091171
Se_0.5_30_Se6	Salmonella enterica	PRJNA561589	SAMN12720402	SRR10091172
Se_0.05_10_Se8	Salmonella enterica	PRJNA561589	SAMN12720403	SRR10091173
Se_5_20_Se2	Salmonella enterica	PRJNA561589	SAMN12720404	SRR10091174
Se_Ec_50_Se7	Salmonella enterica	PRJNA561589	SAMN12720406	SRR10091175
Se_Lm_10_Se1	Salmonella enterica	PRJNA561589	SAMN12720407	SRR10091176
Se_5_20_Se1	Salmonella enterica	PRJNA561589	SAMN12720409	SRR10091177
Lm_Se_50_Lm5	Listeria	PRJNA561589	SAMN12720408	SRR10091178
