# Supplementary material for: Within-species contamination of bacterial whole-genome sequence data has a greater influence on clustering analyses than between-species contamination
Source: Genome Biol. 2019 Dec 18;20:286. doi: 10.1186/s13059-019-1914-x (PMC6918607; doi:10.1186/s13059-019-1914-x)
Supplement: Supplementary file 3 — Additional file 3. Review history. [file 13059_2019_1914_MOESM3_ESM.docx]

Review History

**First round of review**

**Reviewer 1**

**Are you able to assess all statistics in the manuscript, including the appropriateness of statistical tests used?** Yes, and I have assessed the statistics in my report.

**Comments to author:**

The manuscript uses simulated reads to address the important question of the affect of contamination on two major types of analysis performed to assess the relatedness of pathogen samples using whole genome sequences, that of de novo assembly and cgMLST calling and reference-based mapping and SNP calling. Since contamination of samples sequence by WGS is a common occurrence this of interest to the field and helps to quantify the affect of contamination on the results reported from such samples.
The methods are well described and provide sufficient detail to be reproduced using the data and scripts provide.

Methodology
--------------
The methodology is appropriate in that it uses 'real' genomes that diverge from reference genomes by either 0.05, 0.5 and 5% rather than introducing mutations by simulation. In this way the affect of contamination on variation can be readily assessed using variation that is found within the genuine bacterial populations.
Questions and comments arising:
1) Line 116: "For most subjects, within-species contamination represents i) closely related genomes of the same serotype (0.05%), ii) distantly related genomes of the same serotype (0.5%), and iii) genomes of a different serotype (5%; Supplemental Table 1)."
Using serotype to define similarity boundaries is useful since it links genomic analyses to legacy typing methodologies. However it would also be helpful to determine how these genomic differences are reflected by the common molecular technique of MLST and state what 0.05, 0.5 and 5% mean in terms of MLST sequence types including SLV and DLVs and clonal complexes.

2) Line 120: "We generated simulated reads for closed subject assemblies, within-species draft contaminant assemblies, and between-species draft contaminant assemblies with ART_Illumina v2.5.8"
Why were reads simulated from assemblies given that in many cases the reads were first used to make the same assemblies? Was this to ensure that all reads had the same characteristics?

3) Line 134: "SNP matrices were phylogenetically analyzed with GARLI v2.01.1067"
GARLI takes nucleotide or amino acid data as an input. Can you clarify if by SNP matrix you mean a WGS alignment generated as pseudo genomes versus the reference?

4) Line 141: "Partial alleles are those loci whose lengths are less than 60% of the predicted lengths, and missing alleles are those loci that are less than 60% of predicted lengths and less than 80% identical to the reference."
On what basis were these thresholds chosen? In a cgMLST scheme an allele of 60% the length of the reference gene is most likely not a ortholog.


Results
--------

The results show clearly that the affect of intra-species contamination is more marked that in contamination between species both on SNPs and cgMLST alleles.

Questions and comments arising:
5) Although the differences are at face value different, a simple analysis of statistical significance would give the results more strength.
One aspect that is not drawn out clearly enough is that contamination has an impact on cgMLST at lower levels than found with a reference mapping based approach as seen in supplemental figures 3 and 4. This would be expected since a good reference-based mapping pipeline will exclude SNPS where the minority variant is seen at a low percentage within the pileup. It would be helpful to describe at what point the affect of contamination causes a *significant* difference in either the cgMLST or SNP results. In this way the relative affects can be more easily assessed.
6) In many cases the results of all species appear to be lumped together rather than separating them out by species based on the majority species in the simulated contamination. For instance in Figure 1 and 2, it is not clear what 'self' refers to in these figures. Presumably Self could be one of the 3 species in which case if Self was Se - the metrics when mixed with Se should be excluded. It would be best to separate out these figures by species. The values would then be easier to interpret (e.g Length of genome should be tighter for the self category)

Discussion
-------------
Line 87: "We calculated thresholds that indicate the presence of contamination for L. monocytogenes (50 contigs), S. enterica (100 contigs), and E. coli (400 contigs; Figure 2b red dashed lines). Like N50 thresholds, contig counts below these values do not exclude the possibility of contamination."
It would be worth emphasising that these are thresholds based on both the sequencing technology used and the assembler. Longer reads and or a different assembly software would likely result in different thresholds.

Conclusion
------------
Line 98: "This study shows that studies should include steps (such as assessment of assembly metrics) to reduce the influences of within-species contamination."
This study shows that contamination at high levels can significantly impact the results of both cgMLST and SNP analysis. Assembly metrics will not reduce the influence but rather identify samples in which this could be a problem. Other methods of detecting contamination may be more sensitive e.g read taxonomic classification. This should be mentioned as another alternative.

**Reviewer 2**

**Are you able to assess all statistics in the manuscript, including the appropriateness of statistical tests used?** No, I do not feel adequately qualified to assess the statistics.

**Comments to author:**

The authors have generated and analyzed a range of simulated sets of Illumina sequencing reads to investigate the impact of contamination on the accuracy of SNP discovery, multilocus sequence typing and phylogenetic analysis. They report that contamination with closely related (i.e. within-species) sequences may result in considerable sequencing error, while contamination with more distantly related sequences (from different genera) generally causes fewer errors.

The authors also suggest that assembly metrics (N50, number of contigs) may predict the presence of contamination and even propose some species-specific thresholds.


The results are interesting and potentially important. However, it is not clear, how widespread within-species contamination may be in real-life datasets, and particularly in data from bacterial 'pure' cultures that are meant to represent mono-clonal isolates. In fact, it is a weakness of this manuscript that it does not cite any evidence for the existence of a contamination problem.

Contamination with closely related sequences will be particularly difficult to detect, and it would be useful to know if any appropriate tools exist for this purpose. While the authors cite a range of tools for contamination detection (l. 26-27), I wonder why they do not apply and test any of them on their own simulated data.

The authors propose to apply the N50 and the number of assembled contigs to predict contamination (lines 15, 39, 85-89). However, these metrics will depend on many other parameters (e. g., sequencing depth, read length, read quality etc.) and hence are unlikely to provide specific information on contamination in real-life datasets.

If the authors are indeed able to detect within-species contamination in real datasets, however, I suggest they should use their method for screening publicly available datasets for contamination to illustrate the scale of the problem.

Legends to Figures 1 and 2 are insufficient and should be expanded. It remains unclear what exactly was done here and what is illustrated until one reads the legends to Supplementary Figures, which are structured in a similar way.

**Authors Response**

**Point-by-point responses to the reviewers’ comments:**

Reviewer #1: The manuscript uses simulated reads to address the important question of the affect of contamination on two major types of analysis performed to assess the relatedness of pathogen samples using whole genome sequences, that of de novo assembly and cgMLST calling and reference-based mapping and SNP calling. Since contamination of samples sequence by WGS is a common occurrence this of interest to the field and helps to quantify the affect of contamination on the results reported from such samples.
The methods are well described and provide sufficient detail to be reproduced using the data and scripts provide.

Methodology
--------------
The methodology is appropriate in that it uses 'real' genomes that diverge from reference genomes by either 0.05, 0.5 and 5% rather than introducing mutations by simulation. In this way the affect of contamination on variation can be readily assessed using variation that is found within the genuine bacterial populations.
Questions and comments arising:
1) Line 116: "For most subjects, within-species contamination represents i) closely related genomes of the same serotype (0.05%), ii) distantly related genomes of the same serotype (0.5%), and iii) genomes of a different serotype (5%; Supplemental Table 1)."
Using serotype to define similarity boundaries is useful since it links genomic analyses to legacy typing methodologies. However it would also be helpful to determine how these genomic differences are reflected by the common molecular technique of MLST and state what 0.05, 0.5 and 5% mean in terms of MLST sequence types including SLV and DLVs and clonal complexes.

Response: We defined the sequence types for each subject and within-species contaminant using the “mlst” program and PubMLST databases. The sequence types, clonal complexes, and variant counts are included in Supplemental Table 1. In addition, we edited the text (line 188) to incorporate these metrics.

2) Line 120: "We generated simulated reads for closed subject assemblies, within-species draft contaminant assemblies, and between-species draft contaminant assemblies with ART_Illumina v2.5.8"
Why were reads simulated from assemblies given that in many cases the reads were first used to make the same assemblies? Was this to ensure that all reads had the same characteristics?

Response: At this step, we generated simulated reads from assemblies of real sequencing data. We edited the text to clarify this point (line 200).

3) Line 134: "SNP matrices were phylogenetically analyzed with GARLI v2.01.1067"
GARLI takes nucleotide or amino acid data as an input. Can you clarify if by SNP matrix you mean a WGS alignment generated as pseudo genomes versus the reference?

Response: It is an alignment of SNPs that was generated by comparing pseudo-genomes to the reference. We edited the text to clarify this point (line 213).

4) Line 141: "Partial alleles are those loci whose lengths are less than 60% of the predicted lengths, and missing alleles are those loci that are less than 60% of predicted lengths and less than 80% identical to the reference."
On what basis were these thresholds chosen? In a cgMLST scheme an allele of 60% the length of the reference gene is most likely not a ortholog.

Response: The thresholds were chosen in conjunction with the selection of loci to develop a modified core (called a “high-confidence” core) that ensures that only the intended targets are retrieved during BLAST searches. A full description and validation of the *Listeria monocytogenes* version is provided in: <https://bmcmicrobiol.biomedcentral.com/articles/10.1186/s12866-015-0526-1>. In addition, we tested the *Salmonella enterica* version and found it to be highly accurate (<https://journals.plos.org/plosone/article?id=10.1371/journal.pone.0166162>).

Results
--------
The results show clearly that the affect of intra-species contamination is more marked that in contamination between species both on SNPs and cgMLST alleles.

Questions and comments arising:
5) Although the differences are at face value different, a simple analysis of statistical significance would give the results more strength.
One aspect that is not drawn out clearly enough is that contamination has an impact on cgMLST at lower levels than found with a reference mapping based approach as seen in supplemental figures 3 and 4. This would be expected since a good reference-based mapping pipeline will exclude SNPS where the minority variant is seen at a low percentage within the pileup. It would be helpful to describe at what point the affect of contamination causes a *significant* difference in either the cgMLST or SNP results. In this way the relative affects can be more easily assessed.

Response: We have included tests for significance (Additional file 4) and incorporated the information into the text. We also included a line to make the point that errors at lower levels might be expected from an MLST that lacks the filtering that a SNP pipeline has (line 75).

6) In many cases the results of all species appear to be lumped together rather than separating them out by species based on the majority species in the simulated contamination. For instance in Figure 1 and 2, it is not clear what 'self' refers to in these figures. Presumably Self could be one of the 3 species in which case if Self was Se - the metrics when mixed with Se should be excluded. It would be best to separate out these figures by species. The values would then be easier to interpret (e.g Length of genome should be tighter for the self category)

Response: We replaced Figure 1 with Figures 1 and 2, which show the data separated by species. We also edited the text accordingly.

Discussion
-------------
Line 87: "We calculated thresholds that indicate the presence of contamination for L. monocytogenes (50 contigs), S. enterica (100 contigs), and E. coli (400 contigs; Figure 2b red dashed lines). Like N50 thresholds, contig counts below these values do not exclude the possibility of contamination."
It would be worth emphasising that these are thresholds based on both the sequencing technology used and the assembler. Longer reads and or a different assembly software would likely result in different thresholds.

Response: We removed the section about using assembly metrics to predict contamination as there is no correlation with the NCBI sequence data (please see scatter plots below).

Conclusion
------------
Line 98: "This study shows that studies should include steps (such as assessment of assembly metrics) to reduce the influences of within-species contamination."
This study shows that contamination at high levels can significantly impact the results of both cgMLST and SNP analysis. Assembly metrics will not reduce the influence but rather identify samples in which this could be a problem. Other methods of detecting contamination may be more sensitive e.g read taxonomic classification. This should be mentioned as another alternative.

Response: changed to ”Therefore, it is important that studies include steps to detect within-species contamination.” (line 170).

Reviewer #2: The authors have generated and analyzed a range of simulated sets of Illumina sequencing reads to investigate the impact of contamination on the accuracy of SNP discovery, multilocus sequence typing and phylogenetic analysis. They report that contamination with closely related (i.e. within-species) sequences may result in considerable sequencing error, while contamination with more distantly related sequences (from different genera) generally causes fewer errors.

The authors also suggest that assembly metrics (N50, number of contigs) may predict the presence of contamination and even propose some species-specific thresholds.


The results are interesting and potentially important. However, it is not clear, how widespread within-species contamination may be in real-life datasets, and particularly in data from bacterial 'pure' cultures that are meant to represent mono-clonal isolates. In fact, it is a weakness of this manuscript that it does not cite any evidence for the existence of a contamination problem.

Contamination with closely related sequences will be particularly difficult to detect, and it would be useful to know if any appropriate tools exist for this purpose. While the authors cite a range of tools for contamination detection (l. 26-27), I wonder why they do not apply and test any of them on their own simulated data.

Response: As part of the analysis of data submitted to NCBI as mono-clonal isolates, we analyzed the simulated data generated during this study (described beginning with line 131) using the program ConFindr (<https://peerj.com/articles/6995/>). ConFindr detects within-species contamination by using an rMLST (made up of single-copy loci). The presence of SNPs in the reads indicates that more than one copy of a locus is present. The developers compared ConFindr to other read-based contamination detection tools. Rather than replicate this work, we decided to build upon it by using the simulated dataset to estimate the amounts of contamination in public databases that are likely to cause errors in SNP and MLST analyses.

The authors propose to apply the N50 and the number of assembled contigs to predict contamination (lines 15, 39, 85-89). However, these metrics will depend on many other parameters (e. g., sequencing depth, read length, read quality etc.) and hence are unlikely to provide specific information on contamination in real-life datasets.

Response: We have removed the section about using assembly metrics to predict contamination as analysis of NCBI sequence data indicates that there is no correlation between numbers of contigs and levels of contamination (please see scatter plots below).

Ec

| ConFindr SNPs | Contigs |
| --- | --- |
| 3 | 90 |
| 1 | 90 |
| 0 | 242 |
| 0 | 242 |
| 0 | 235 |
| 0 | 235 |
| 1 | 139 |
| 2 | 139 |
| 0 | 151 |
| 0 | 151 |
| 0 | 240 |
| 0 | 240 |
| 0 | 148 |
| 1 | 148 |
| 1 | 183 |
| 0 | 183 |
| 0 | 155 |
| 2 | 155 |
| 0 | 163 |
| 0 | 163 |
| 0 | 205 |
| 0 | 205 |
| 1 | 232 |
| 0 | 232 |
| 0 | 194 |
| 1 | 194 |
| 0 | 103 |
| 0 | 103 |
| 0 | 109 |
| 0 | 109 |
| 0 | 109 |
| 0 | 109 |
| 0 | 165 |
| 0 | 165 |
| 0 | 2 |
| 0 | 2 |
| 0 | 2 |
| 0 | 2 |
| 1 | 4 |
| 0 | 174 |
| 0 | 174 |
| 1 | 117 |
| 3 | 117 |
| 0 | 218 |
| 0 | 218 |
| 0 | 3 |
| 0 | 3 |
| 0 | 313 |
| 0 | 313 |
| 0 | 4 |
| 0 | 4 |
| 0 | 4 |
| 0 | 4 |
| 0 | 6 |
| 0 | 6 |
| 0 | 2 |
| 0 | 2 |
| 0 | 1 |
| 0 | 4 |
| 0 | 4 |
| 0 | 296 |
| 0 | 296 |
| 0 | 179 |
| 1 | 179 |
| 0 | 5 |
| 0 | 5 |
| 0 | 1 |
| 0 | 1 |
| 0 | 2 |
| 0 | 2 |
| 0 | 89 |
| 0 | 89 |
| 2 | 56 |
| 0 | 56 |
| 0 | 211 |
| 0 | 211 |
| 1 | 107 |
| 1 | 107 |
| 0 | 122 |
| 0 | 122 |
| 0 | 34 |
| 0 | 34 |
| 0 | 124 |
| 0 | 124 |
| 0 | 105 |
| 0 | 105 |
| 0 | 157 |
| 0 | 157 |
| 0 | 110 |
| 0 | 110 |
| 0 | 75 |
| 0 | 75 |
| 0 | 117 |
| 0 | 117 |
| 0 | 114 |
| 0 | 114 |
| 0 | 186 |
| 0 | 186 |
| 2 | 126 |
| 0 | 126 |
| 0 | 81 |
| 0 | 81 |
| 0 | 87 |
| 0 | 87 |
| 0 | 71 |
| 0 | 71 |
| 0 | 51 |
| 0 | 51 |
| 0 | 75 |
| 0 | 75 |
| 0 | 108 |
| 0 | 108 |
| 0 | 217 |
| 0 | 142 |
| 0 | 142 |
| 0 | 86 |
| 0 | 86 |
| 0 | 93 |
| 0 | 93 |
| 3 | 208 |
| 0 | 208 |
| 0 | 80 |
| 0 | 80 |
| 0 | 174 |
| 0 | 174 |
| 0 | 206 |
| 0 | 206 |
| 0 | 78 |
| 0 | 78 |
| 1 | 197 |
| 0 | 197 |
| 1 | 108 |
| 0 | 108 |
| 0 | 101 |
| 0 | 101 |
| 2 | 135 |
| 0 | 135 |
| 0 | 89 |
| 0 | 89 |
| 0 | 137 |
| 0 | 137 |
| 0 | 64 |
| 2 | 64 |
| 0 | 71 |
| 0 | 71 |
| 0 | 133 |
| 0 | 133 |
| 0 | 71 |
| 0 | 71 |
| 1 | 94 |
| 0 | 94 |
| 0 | 90 |
| 0 | 90 |
| 0 | 106 |
| 0 | 106 |
| 1 | 147 |
| 1 | 147 |
| 0 | 130 |
| 0 | 130 |
| 0 | 111 |
| 0 | 111 |
| 0 | 251 |
| 1 | 251 |
| 0 | 181 |
| 0 | 181 |
| 0 | 283 |
| 0 | 283 |
| 0 | 94 |
| 0 | 94 |
| 0 | 288 |
| 1 | 288 |
| 2 | 118 |
| 0 | 118 |
| 0 | 111 |
| 0 | 111 |
| 0 | 204 |
| 0 | 204 |
| 0 | 316 |
| 0 | 316 |
| 0 | 175 |
| 0 | 175 |
| 0 | 96 |
| 0 | 96 |
| 0 | 221 |
| 0 | 221 |
| 0 | 272 |
| 0 | 272 |
| 0 | 140 |
| 0 | 140 |
| 0 | 82 |
| 0 | 82 |
| 0 | 112 |
| 0 | 112 |
| 0 | 138 |
| 1 | 138 |
| 0 | 174 |
| 1 | 174 |
| 0 | 141 |

Lm

| ConFindr SNPs | Contigs |
| --- | --- |
| 2 | 58 |
| 1 | 167 |
| 0 | 167 |
| 0 | 45 |
| 0 | 45 |
| 0 | 39 |
| 0 | 39 |
| 0 | 20 |
| 0 | 20 |
| 0 | 30 |
| 0 | 30 |
| 0 | 77 |
| 0 | 77 |
| 0 | 80 |
| 2 | 80 |
| 0 | 44 |
| 0 | 44 |
| 0 | 36 |
| 0 | 36 |
| 0 | 22 |
| 0 | 22 |
| 0 | 37 |
| 0 | 37 |
| 0 | 18 |
| 0 | 18 |
| 0 | 35 |
| 0 | 35 |
| 0 | 58 |
| 0 | 58 |
| 0 | 156 |
| 0 | 156 |
| 0 | 36 |
| 0 | 36 |
| 0 | 17 |
| 0 | 17 |
| 101 | 278 |
| 96 | 278 |
| 0 | 31 |
| 0 | 31 |
| 0 | 65 |
| 0 | 65 |
| 0 | 69 |
| 0 | 69 |
| 43 | 361 |
| 47 | 361 |
| 0 | 45 |
| 1 | 45 |
| 0 | 31 |
| 0 | 31 |
| 1 | 33 |
| 2 | 33 |
| 0 | 21 |
| 0 | 21 |
| 7 | 226 |
| 3 | 226 |
| 0 | 34 |
| 0 | 34 |
| 0 | 36 |
| 0 | 36 |
| 0 | 41 |
| 0 | 41 |
| 0 | 34 |
| 0 | 34 |
| 0 | 28 |
| 0 | 28 |
| 0 | 13 |
| 0 | 13 |
| 0 | 78 |
| 0 | 78 |
| 0 | 20 |
| 0 | 20 |
| 0 | 33 |
| 1 | 33 |
| 0 | 33 |
| 0 | 33 |
| 0 | 42 |
| 1 | 42 |
| 0 | 38 |
| 0 | 38 |
| 0 | 64 |
| 0 | 64 |
| 1 | 52 |
| 5 | 46 |
| 0 | 46 |
| 0 | 24 |
| 0 | 24 |
| 2 | 99 |
| 1 | 99 |
| 0 | 161 |
| 0 | 161 |
| 0 | 48 |
| 0 | 48 |
| 0 | 27 |
| 0 | 27 |
| 0 | 26 |
| 0 | 26 |
| 0 | 121 |
| 0 | 121 |
| 0 | 27 |
| 1 | 27 |
| 0 | 102 |
| 0 | 102 |
| 0 | 32 |
| 1 | 32 |
| 0 | 25 |
| 0 | 25 |
| 0 | 23 |
| 0 | 23 |
| 0 | 49 |
| 0 | 49 |
| 0 | 15 |
| 0 | 15 |
| 0 | 28 |
| 0 | 28 |
| 0 | 24 |
| 0 | 24 |
| 0 | 27 |
| 0 | 27 |
| 0 | 88 |
| 0 | 88 |
| 0 | 74 |
| 1 | 74 |
| 0 | 32 |
| 0 | 32 |
| 0 | 110 |
| 0 | 110 |
| 0 | 12 |
| 1 | 12 |
| 0 | 88 |
| 1 | 88 |
| 1 | 13 |
| 0 | 13 |
| 0 | 77 |
| 0 | 77 |
| 1 | 38 |
| 2 | 38 |
| 0 | 16 |
| 0 | 16 |
| 0 | 43 |
| 0 | 43 |
| 0 | 51 |
| 0 | 51 |
| 0 | 59 |
| 1 | 59 |
| 0 | 34 |
| 0 | 34 |
| 1 | 80 |
| 0 | 80 |
| 0 | 30 |
| 0 | 30 |
| 0 | 52 |
| 0 | 52 |
| 1 | 18 |
| 1 | 18 |
| 4 | 22 |
| 1 | 22 |
| 0 | 21 |
| 0 | 21 |
| 0 | 23 |
| 0 | 23 |
| 0 | 14 |
| 0 | 14 |
| 0 | 19 |
| 0 | 19 |
| 0 | 55 |
| 0 | 55 |
| 0 | 22 |
| 1 | 22 |
| 0 | 103 |
| 0 | 103 |
| 0 | 25 |
| 0 | 25 |
| 0 | 18 |
| 0 | 18 |
| 0 | 46 |
| 0 | 46 |
| 1 | 51 |
| 0 | 51 |
| 0 | 16 |
| 0 | 16 |
| 0 | 83 |
| 0 | 83 |
| 0 | 77 |
| 0 | 77 |
| 0 | 56 |
| 0 | 56 |
| 0 | 68 |
| 0 | 68 |
| 0 | 14 |
| 0 | 14 |
| 0 | 17 |
| 0 | 17 |
| 0 | 154 |
| 0 | 154 |
| 0 | 23 |
| 0 | 23 |
| 0 | 26 |
| 0 | 26 |
| 1 | 19 |
| 0 | 19 |

Se

| ConFindr SNPs | Contigs |
| --- | --- |
| 0 | 72 |
| 0 | 60 |
| 0 | 60 |
| 0 | 51 |
| 0 | 38 |
| 0 | 39 |
| 0 | 65 |
| 0 | 58 |
| 0 | 50 |
| 0 | 73 |
| 0 | 73 |
| 0 | 83 |
| 0 | 83 |
| 0 | 83 |
| 0 | 88 |
| 0 | 88 |
| 0 | 94 |
| 0 | 107 |
| 0 | 91 |
| 0 | 91 |
| 0 | 88 |
| 0 | 88 |
| 0 | 91 |
| 0 | 91 |
| 0 | 87 |
| 0 | 87 |
| 0 | 85 |
| 0 | 91 |
| 0 | 91 |
| 0 | 83 |
| 0 | 84 |
| 0 | 84 |
| 0 | 94 |
| 0 | 104 |
| 0 | 69 |
| 0 | 95 |
| 0 | 92 |
| 0 | 92 |
| 0 | 99 |
| 0 | 72 |
| 0 | 60 |
| 0 | 78 |
| 0 | 105 |
| 0 | 77 |
| 0 | 77 |
| 0 | 105 |
| 0 | 105 |
| 0 | 76 |
| 0 | 76 |
| 0 | 170 |
| 0 | 170 |
| 0 | 85 |
| 0 | 88 |
| 0 | 92 |
| 0 | 92 |
| 0 | 56 |
| 0 | 56 |
| 0 | 69 |
| 0 | 88 |
| 0 | 93 |
| 0 | 86 |
| 0 | 89 |
| 0 | 91 |
| 0 | 91 |
| 0 | 84 |
| 0 | 84 |
| 0 | 91 |
| 0 | 89 |
| 0 | 83 |
| 0 | 87 |
| 0 | 76 |
| 0 | 87 |
| 0 | 98 |
| 0 | 98 |
| 0 | 99 |
| 0 | 94 |
| 0 | 85 |
| 0 | 85 |
| 0 | 90 |
| 0 | 65 |
| 0 | 60 |
| 0 | 57 |
| 0 | 59 |
| 0 | 59 |
| 0 | 63 |
| 0 | 64 |
| 0 | 85 |
| 0 | 64 |
| 0 | 2 |
| 0 | 51 |
| 0 | 51 |
| 0 | 98 |
| 0 | 75 |
| 0 | 75 |
| 0 | 60 |
| 0 | 60 |
| 0 | 60 |
| 0 | 88 |
| 0 | 88 |
| 0 | 99 |
| 0 | 247 |
| 0 | 296 |
| 0 | 86 |
| 0 | 34 |
| 0 | 34 |
| 0 | 39 |
| 0 | 198 |
| 0 | 198 |
| 0 | 112 |
| 0 | 112 |
| 0 | 127 |
| 0 | 124 |
| 0 | 1722 |
| 0 | 1722 |
| 0 | 1095 |
| 0 | 1095 |
| 0 | 1164 |
| 0 | 1164 |
| 0 | 1478 |
| 0 | 1478 |
| 0 | 2131 |
| 0 | 2131 |
| 0 | 1686 |
| 0 | 1686 |
| 0 | 2120 |
| 0 | 2120 |
| 0 | 2175 |
| 0 | 2175 |
| 0 | 2047 |
| 0 | 2047 |
| 0 | 2197 |
| 0 | 86 |
| 0 | 86 |
| 0 | 100 |
| 0 | 100 |
| 0 | 96 |
| 0 | 96 |
| 0 | 77 |
| 0 | 77 |
| 0 | 88 |
| 0 | 88 |
| 0 | 79 |
| 0 | 79 |
| 0 | 96 |
| 0 | 96 |
| 0 | 28 |
| 0 | 45 |
| 0 | 45 |
| 0 | 36 |
| 0 | 36 |
| 0 | 38 |
| 0 | 33 |
| 0 | 33 |
| 0 | 77 |
| 0 | 77 |
| 0 | 38 |
| 0 | 117 |
| 0 | 117 |
| 0 | 55 |
| 0 | 55 |
| 0 | 41 |
| 0 | 58 |
| 0 | 49 |
| 0 | 49 |
| 0 | 31 |
| 0 | 44 |
| 0 | 52 |
| 0 | 52 |
| 0 | 53 |
| 0 | 52 |
| 0 | 55 |
| 0 | 55 |
| 0 | 69 |
| 0 | 69 |
| 0 | 128 |
| 0 | 42 |
| 0 | 42 |
| 0 | 47 |
| 0 | 47 |
| 0 | 51 |
| 0 | 51 |
| 0 | 99 |
| 0 | 99 |
| 0 | 43 |
| 0 | 43 |
| 0 | 63 |
| 0 | 50 |
| 0 | 1840 |
| 0 | 60 |
| 0 | 71 |
| 0 | 71 |
| 0 | 66 |
| 0 | 81 |
| 0 | 117 |
| 0 | 30 |
| 0 | 30 |
| 0 | 54 |
| 0 | 54 |
| 0 | 65 |
| 0 | 46 |

If the authors are indeed able to detect within-species contamination in real datasets, however, I suggest they should use their method for screening publicly available datasets for contamination to illustrate the scale of the problem.

Response: We analyzed 10,000 fastq datasets obtained from NCBI for each species. We found that there is significant contamination in the data (Additional file 5 and Supplemental table 1; line 126).

Legends to Figures 1 and 2 are insufficient and should be expanded. It remains unclear what exactly was done here and what is illustrated until one reads the legends to Supplementary Figures, which are structured in a similar way.

Response: The legends have been expanded to include more details about the method (lines 337 and 343).

**Second round of review**

**Reviewer 2**

All my comments on the previous version of the manuscript have been considered sufficiently and changes were made where appropriate. I have no further comments.
